# Structural analysis of carbohydrate binding by the macrophage mannose receptor CD206

**DOI:** 10.1016/j.jbc.2021.100368

**Published:** 2021-02-02

**Authors:** Hadar Feinberg, Sabine A.F. Jégouzo, Yi Lasanajak, David F. Smith, Kurt Drickamer, William I. Weis, Maureen E. Taylor

**Affiliations:** 1 Departments of Structural Biology and Molecular and Cellular Physiology, Stanford University School of Medicine, Stanford, California, USA; 2 Department of Life Sciences, Imperial College London, London, United Kingdom; 3 Emory Comprehensive Glycomics Core, Emory University School of Medicine, Atlanta, Georgia, USA

**Keywords:** CD206, carbohydrate-binding protein, scavenger receptor, crystal structure, C-type lectin, glycobiology, glycoprotein, CTLD, C-type lectin-like domain, CRD, carbohydrate-recognition domain, DC-SIGN, dendritic-cell-specific ICAM-3-grabbing nonintegrin

## Abstract

The human mannose receptor expressed on macrophages and hepatic endothelial cells scavenges released lysosomal enzymes, glycopeptide fragments of collagen, and pathogenic microorganisms and thus reduces damage following tissue injury. The receptor binds mannose, fucose, or N-acetylglucosamine (GlcNAc) residues on these targets. C-type carbohydrate-recognition domain 4 (CRD4) of the receptor contains the site for Ca^2+^-dependent interaction with sugars. To investigate the details of CRD4 binding, glycan array screening was used to identify oligosaccharide ligands. The strongest signals were for glycans that contain either Manα1-2Man constituents or fucose in various linkages. The mechanisms of binding to monosaccharides and oligosaccharide substructures present in many of these ligands were examined in multiple crystal structures of CRD4. Binding of mannose residues to CRD4 results primarily from interaction of the equatorial 3- and 4-OH groups with a conserved principal Ca^2+^ common to almost all sugar-binding C-type CRDs. In the Manα1-2Man complex, supplementary interactions with the reducing mannose residue explain the enhanced affinity for this disaccharide. Bound GlcNAc also interacts with the principal Ca^2+^ through equatorial 3- and 4-OH groups, whereas fucose residues can bind in several orientations, through either the 2- and 3-OH groups or the 3- and 4-OH groups. Secondary contacts with additional sugars in fucose-containing oligosaccharides, such as the Lewis-a trisaccharide, provide enhanced affinity for these glycans. These results explain many of the biologically important interactions of the mannose receptor with both mammalian glycoproteins and microbes such as yeast and suggest additional classes of ligands that have not been previously identified.

The mannose receptor (CD206) is expressed on macrophages, dendritic cells, and sinusoidal endothelial cells of the liver (1, 2). It is a scavenger receptor that binds both endogenous mammalian glycans and sugars on the surfaces of microorganisms (3, 4, 5). Endogenous ligands include released lysosomal enzymes, fragments of collagen and other proteins generated at sites of tissue damage, and pituitary hormones (6, 7). Binding to the receptor leads to endocytosis of these ligands, resulting in rapid and efficient clearance of glycoproteins from circulation. The ability of the mannose receptor to facilitate uptake of glycoproteins bearing high-mannose oligosaccharides is used to target therapeutic glycoproteins to macrophages, particularly in the treatment of lysosomal storage diseases (8, 9). The mannose receptor also functions as part of the innate immune system. Sugar structures on microorganisms targeted by the receptor include mannans on the surface of yeasts, mannose-capped lipo-arabinomannans on mycobacteria, and high-mannose oligosaccharides on the surfaces of viruses (10, 11, 12).

These diverse ligands are bound to three different types of protein domains in the extracellular portion of the mannose receptor (Fig. 1). The N-terminal domain is an R-type carbohydrate-recognition domain, related in sequence and structure to the sugar-binding portion of ricin (14, 15). This domain binds to 4-sulfo-GalNAc residues that are displayed at the nonreducing ends of glycans on specific glycoproteins, including luteinizing hormone (18). The adjacent fibronectin type II domain binds to fragments of collagens that contain triple helical regions (16, 19). The remainder of the extracellular portion of the receptor consists of eight C-type lectin-like domains (CTLDs^2^). CTLDs 4 and 5 account for most of the sugar-binding activity, and CTLD4 is the only one of the CTLDs that binds sugars in the absence of other domains (13, 20). These two domains are therefore usually designated carbohydrate-recognition domains (CRDs) 4 and 5. Interaction of these domains with mannose-containing oligosaccharides accounts for binding of the receptor to lysosomal enzymes, nonhelical fragments of collagen, and viral glycoproteins and can also account for binding to mannose residues on the surfaces of other pathogens. Internalization of ligands is mediated by clathrin-coated vesicles for trafficking to endosomes and lysosomes.

**Figure 1:**
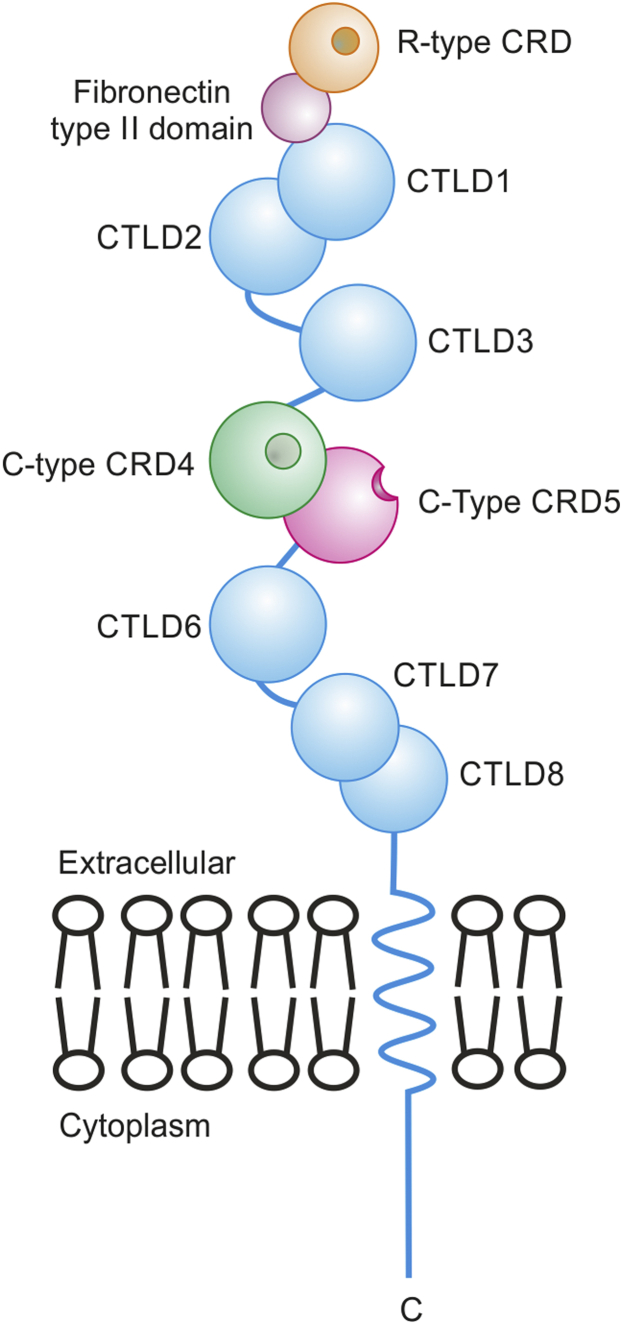
**Domain organization of the mannose receptor.** Type I transmembrane orientation of the receptor and three types of ligand-binding domains are indicated. The receptor consists of an N-terminal R-type carbohydrate-recognition domain (CRD), a fibronectin type II domain, and eight C-type lectin-like domains (CTLDs). Binding of neutral oligosaccharides is mediated by the central two CTLDs, which are therefore labeled as CRD4 and CRD5; only CRD4 can bind monosaccharide ligands on its own (13). Crystal structures of the R-type CRD interacting with sulfated oligosaccharides and of the fibronectin type II domain of the related receptor Endo180 interacting with collagen have been described (14, 15, 16). A crystal structure of the first five domains, including the R-type CRD, the fibronectin type II repeat, and CTLDs 1–3 of the mannose receptor has been obtained (PDB entry 6INN) (17), but no structure of a C-type CRD with bound ligand has been described previously.

The mannose receptor is a prototype for a family of proteins that have similar domain organization. The other three members of the family are Endo180, also known as urokinase plasminogen activator receptor-associated protein or uPARAP, the phospholipase A_2_ receptor, and DEC-205 (21). All four proteins are conserved throughout mammalian evolution. The endothelial receptor Endo180 is the only other member of the family with sugar-binding activity, but in this case it is CTLD2 that binds selectively to GlcNAc residues (22).

Previous binding studies of the interactions of mannose receptor CRD4, performed by competition binding assays and by NMR titration, revealed binding to a range of monosaccharides including mannose, GlcNAc, and glucose (23, 24, 25). These sugars all have adjacent, equatorial 3- and 4-OH groups that can interact with Ca^2+^ in C-type CRDs, as originally described for serum mannose-binding protein (26). Like binding sites in many C-type CRDs that bind these sugars, CRD4 also binds to fucose but not to galactose.

Studies of the overall conformation of the extracellular domain of the mannose receptor have been undertaken by electron microscopy and the structure of the N-terminal R-type CRD, fibronectin type II repeat, and first three CTLDs has been determined by X-ray crystallography (17, 27, 28). These results have provided information about the relative positions of the different domains, but ligands were not present so there was no information about how sugars are bound. Crystal structures of the R-type CRD with 4-sulfo-GalNAc and other sulfated sugars have been obtained (14, 15). The interactions observed are consistent with the observed affinities for different ligands. In crystals of a fragment containing CRD4, a domain swap occurred between two copies of the CRD, which precluded ligand binding (29). NMR analysis of ligand binding, combined with extensive mutagenesis of CRD4 and molecular modeling, has provided insights into the likely arrangement of key residues near to the binding site (23, 25). However, up to now there has been no high-resolution structural information about the mode of sugar binding to this key domain.

In the present studies, binding competition experiments, glycan array analysis, and X-ray crystallography have been used to define the binding selectivity of CRD4 and the molecular mechanisms by which this selectivity is achieved.

## Results

### Expression of CRD4 with N- and C-terminal modifications

In an effort to increase yields and generate novel crystal forms of CRD4 that avoid formation of domain-swapped dimers seen in previous experiments, the expression construct was modified by changing the expression system and removing potentially flexible regions. Interdomain linkers on either side of CRD4 were retained in the CRD4 expression system employed previously, in which the ompA signal sequence was used to direct CRD4 to the periplasm of *Escherichia coli* to facilitate disulfide bond formation (24). In these earlier experiments, folded CRD4 was extracted from the periplasm and purified by affinity chromatography. To increase the yield of protein in the present studies, CRD4 was expressed in a T7-based system without a signal sequence. In this system, placing the sequence MetAla at the N terminus leads to cleavage of the methionine residue within the bacteria to yield a free N-terminal alanine residue, which represents a minimal modification of the natural protein sequence.

**Figure 2:**
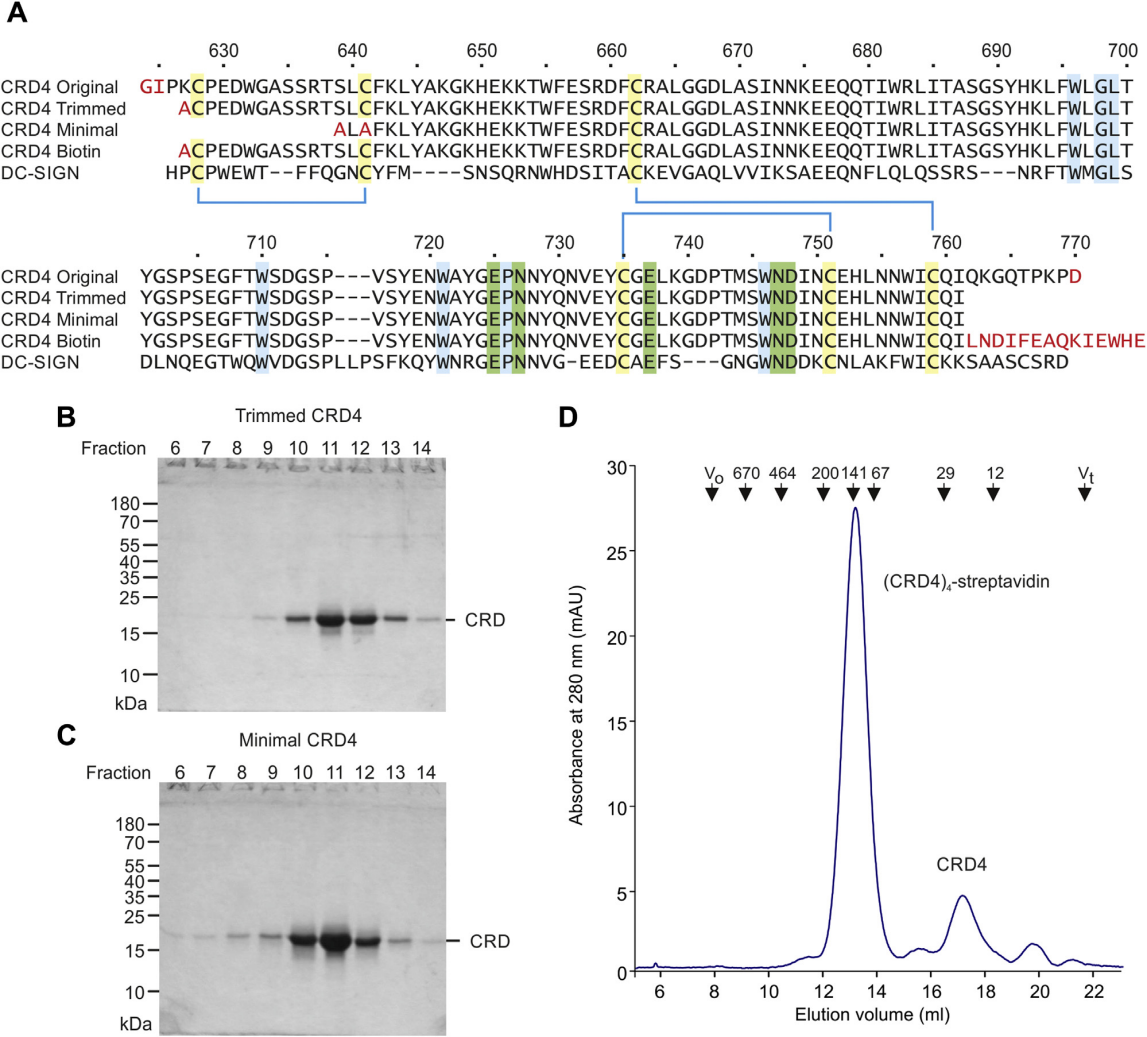
**Expression of modified CRD4 for binding studies and crystallization.***A*, Sequences of the previously expressed version of the CRD4, the current version trimmed at each end, a further N-terminal truncated, minimal CRD, and a biotin-tagged construct. The sequence of the CRD from DC-SIGN is also shown for comparison. Residues introduced in the expression vectors are shown in *red* type. Cysteine residues linked in disulfide bonds are highlighted in *yellow* and are connected with *blue lines*. Amino acids that are ligands for the conserved principal Ca^2+^ are highlighted in *green* and other commonly conserved residues in C-type CRDs are highlighted in *blue*. *B/C*, purification of trimmed and minimal CRD4 by affinity chromatography on mannose-Sepharose. SDS–polyacrylamide gel electrophoresis of aliquots from 1-ml fractions eluted from a 10-ml column with EDTA. Gels were stained with Coomassie blue. *D*, gel filtration chromatography of biotinylated CRD4 complexed with fluorescently-labeled streptavidin on a Superdex S200 column.

The connecting sequences at either end of CRD4, which are proline-rich and contain potential O-linked glycosylation sites, may be quite flexible in conformation (30). Therefore, the length of the expression construct was reduced based on the structure deduced in the earlier studies (29), with the goal of facilitating crystallization in alternative lattice arrangements while retaining an active CRD. Initially, residues at the N and C termini were removed if they were not seen in all copies of the protomer in the earlier crystal structures (Fig. 2*A*). Thus, the N-terminal sequence of the purified, modified protein is AlaCys. This cysteine residue is linked in a disulfide bond, so the new version represents the minimum N-terminal sequence compatible with correct formation of all of the disulfide bonds. At the C terminus, residues up to the sequence CysGlnIleGln are visible in all copies in the earlier crystal structures. However, while the side chain of the isoleucine residue points inward, the C-terminal glutamine residue points mostly away from the protein surface. Therefore, the expression construct, designated “trimmed CRD4,” was terminated with the sequence CysGlnIle. A minimal CRD4, generated by further N-terminal truncation, lacks most of the residues up to the first β strand seen in the original crystals. As part of this truncation, a cysteine residue was deleted, so the cysteine residue that would normally be paired with this residue was mutated to alanine.

Expression of these modified CRD4 constructs without a signal sequence resulted in formation of inclusion bodies that were dissolved in guanidine and dialyzed to allow renaturation and disulfide bond formation. The folded proteins were purified by affinity chromatography on mannose-Sepharose (Fig. 2, *B* and *C*). The fact that the minimal CRD4 can be purified by affinity chromatography on a sugar ligand confirms that it retains a functional ligand-binding site. A biotin-tagged version of the trimmed CRD4 was also generated by appending a biotinylation target sequence onto the C terminus (Fig. 2*A*). Biotinylation of the single lysine residue in this sequence can be catalyzed by biotin ligase in the bacterial cytoplasm. The biotinylated CRD, purified by affinity chromatography, was immobilized on streptavidin-coated plates for binding assays. In addition, the biotinylated CRD was incubated with fluorescently labeled streptavidin to form a tetrameric complex, which was repurified by affinity chromatography for use in screening a glycan array (Fig. 2*D*).

### Identification of oligosaccharide ligands for CRD4 by screening of glycan array

**Figure 3:**
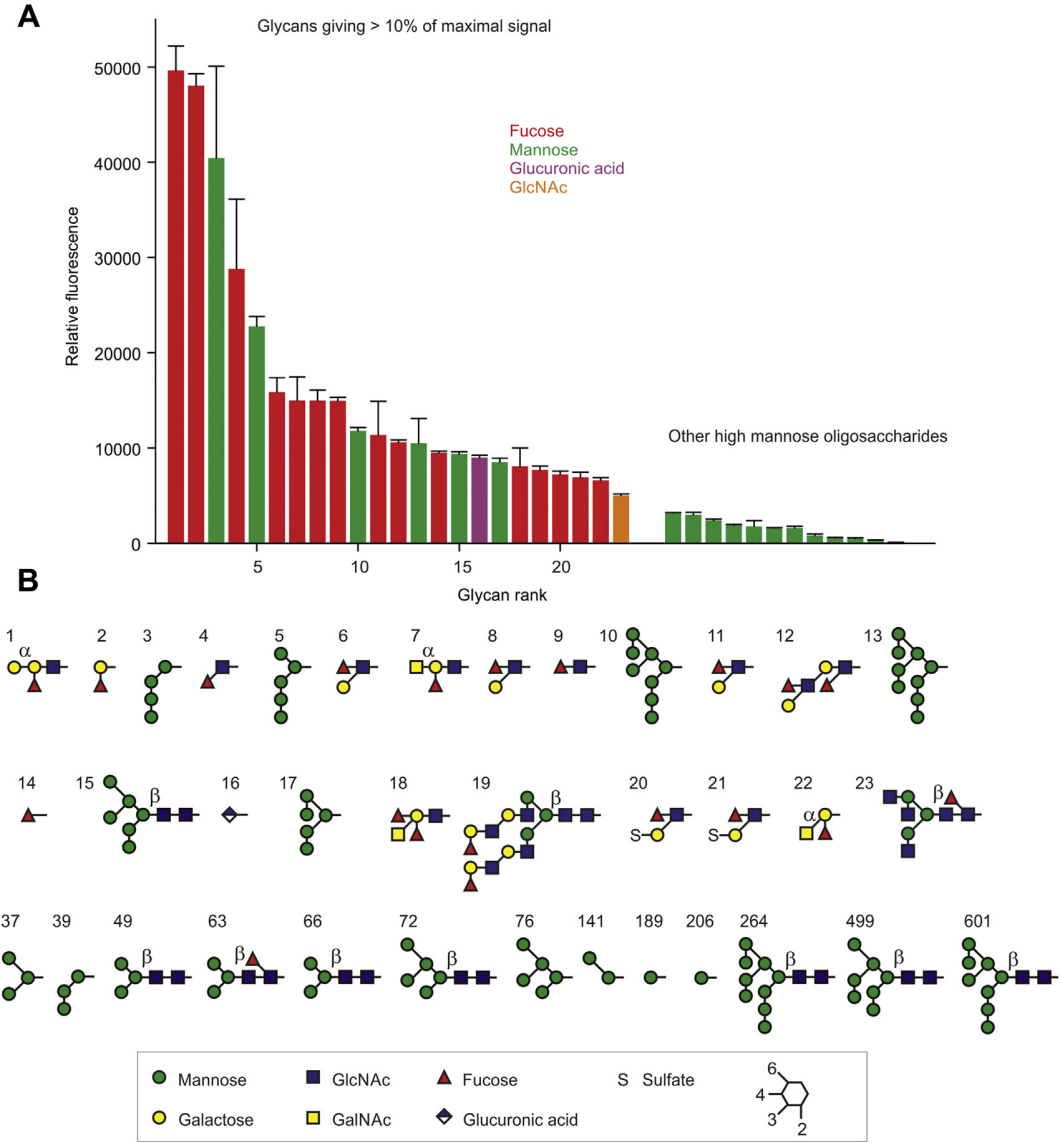
**Screening of glycan array with CRD4.***A*, summary of results for screening of version 6.2 of the Consortium for Functional Glycomics synthetic glycan array with a fluorescent CRD4-streptavidin complex. *Left*, data for the 23 glycans with at least 10% of the fluorescence level of the most intense signal. *Right*, data for the remaining high-mannose oligosaccharides on the array. Results are color-coded based on the monosaccharide, that is suggested to be the main binding epitope. *B*, structures of oligosaccharides corresponding to the binding results in *A*, shown in symbol nomenclature and are numbered based on their rank in the array results. All mannose and fucose linkages are in the α configuration and all other linkages are in the β configuration except as indicated. A complete list of the glycans on the array and the screening results at ∼2.5 mM CaCl_2_, which were used to create this figure, are presented in Table S1 along with results for screening at ∼7.5 mM CaCl_2_ in Table S2.

Two independent screenings of the glycan array developed by the Consortium for Functional Glycomics (31), which contains 601 oligosaccharides, gave very similar results, with the same ligands giving the strongest signals, although the relative intensities showed small variations in a few cases (Tables S1 and S2). Almost all of the oligosaccharides giving strong signals contain either mannose or fucose residues at the nonreducing termini (Fig. 3). All of the mannose-containing ligands are substructures of a Man_9_ high-mannose oligosaccharide. For comparison, the relative rank and structures of the remaining high-mannose oligosaccharides on the array are also shown in Figure 3. The six high-mannose glycans with the strongest signals all contain terminal Manα1-2Man linkages, and only one glycan with Manα1-2Man linkage is ranked further down, at position 39.

In general, the signals for mannose-containing oligosaccharides decrease with increasing size. This trend may reflect preferential binding to the Manα1-2Man epitope, which might become less accessible on larger glycans as a result of conformational differences or steric clashes with additional substituents. The epitopes may also become less abundant as space restricts the density of larger glycans on the array. The mannose receptor can be targeted with a range of mammalian N-linked high-mannose oligosaccharides on glycoproteins, and experiments in knock-out mice confirm its role in clearance of such glycoproteins (7, 9). It also binds to high-mannose oligosaccharides on viruses (11, 12). The array results are also consistent with binding of CRD4 to glycans on the surfaces of microorganisms, such as yeast mannans and mannose-capped lipo-arabinomannans of mycobacteria, which contain Manα1-2Man units (32, 33).

Fucose is likely to be the main binding determinant in most of the remaining ligands, based on evidence that fucose competes for binding to the receptor and to a CRD4-5 fragment, while other sugars in some of the oligosaccharides, such as galactose, are poor ligands (13, 34, 35). There is no simple pattern to the fucose-containing ligands. Ligands that bind strongly include fucose in α1-2, α1-3, and α1-4 linkage, although the α1-4 linkage, as in the Lewis-a trisaccharide in glycans 6, 8, 11, 12, 20, and 21, is somewhat more common. Glycan 4, which lacks galactose, binds better than any of the trisaccharides.

These results suggest that, as in the case of the mannose-containing ligands, the binding epitope is likely to be relatively simple. Other larger glycans that contain terminal fucose with these linkages give considerably weaker signals. Comparison of N-linked glycans in which the chitobiose core is fucosylated or not fucosylated also does not reveal any significant contribution to binding by the core fucose in α1-6 linkage. As with the mannose-containing ligands, these results may indicate that larger glycans decrease the ability of the binding fucose residue to access the binding site, perhaps because of steric occlusion by the rest of the glycan.

Some additional glycans that do not have exposed mannose or fucose residues show binding to CRD4. Glycan 23 contains three terminal GlcNAc residues and other glycans containing one or more terminal GlcNAc residues also rank relatively high at 30, 32–34, 38, 41, and 51–52, with more in the top 100 ligands. These results, showing some binding but generally less than for mannose- and fucose-terminated glycans, are consistent with weaker binding to GlcNAc seen in binding competition assays (23, 25). Glycan 16 is simply glucuronic acid in β linkage to the spacer. The α anomer is ranked at position 593, and several disaccharides that contain β-linked glucuronic acid residues do not show binding above background. Thus, it is not clear whether the binding to glycan 16 represents a biologically meaningful interaction.

Two of the ligands for CRD4 identified in the array screening contain sulfated sugars, although binding of ligands 20 and 21 (Fig. 3*B*) can be explained by the presence of exposed fucose residues in the Lewis-a configuration. In contrast to these results, screening of an earlier version of the array with an Fc chimera that contained the entire extracellular portion of the mannose receptor showed binding to many different sulfated oligosaccharides (36). Binding to these additional sulfated ligands is probably mediated by the N-terminal R-type CRD, which is known to bind the physiological 4-sulfo-GalNAc ligand (18). The present results confirm that binding to most sulfated oligosaccharides is not through CRD4.

### Analysis of CRD4 binding to mannose by X-ray crystallography of trimmed CRD4

In order to investigate the mechanism of binding of sugars to CRD4, the trimmed version of CRD4 (Fig. 2*A*) was used to screen for suitable crystallization conditions in the presence of various ligands. A total of eight different crystal forms with different unit cells and/or different space groups were obtained with different combinations of ligands and crystallization conditions (Table S3). In the following descriptions, each of the different crystal forms, distinguished by the space group symmetry and unit cell parameters (Table S4), is indicated by the number appended to the ligand name. Thus, αMeMan/1 and αMeMan/2 are two crystal forms of CRD4 bound to αMeMan, whereas αMeMan/3 and Lewis-a/3 are different ligand complexes that crystallized in the same crystal form and thus have the same unit cell and symmetry. Data were collected at 1.2–1.75 Ǻ for the various crystals forms (Table S4), and the final refined structures had R_cryst_ values of 13.4–18.9 and R_free_ values of 15.7–22.0 (Table S5).

**Figure 4:**
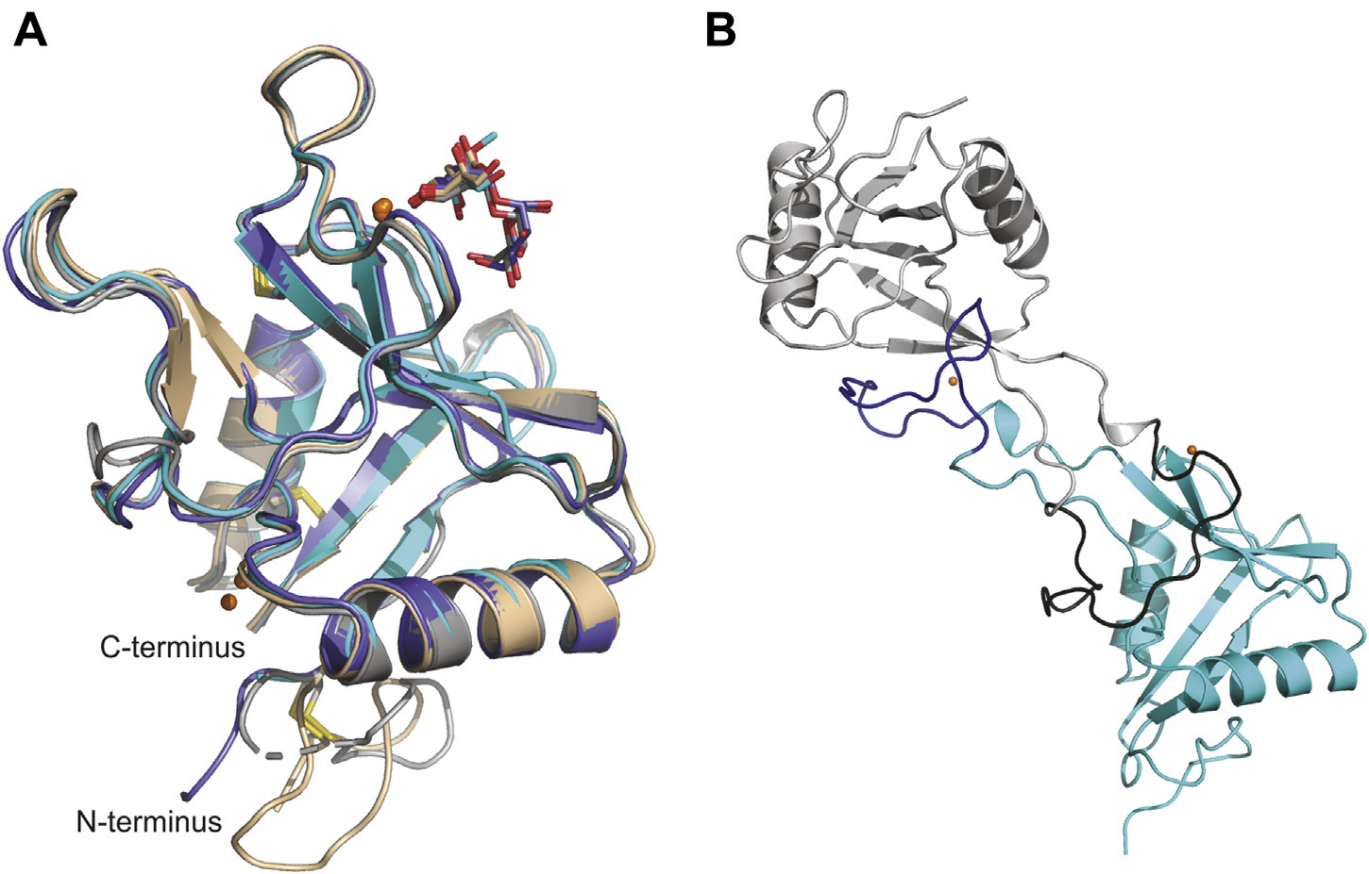
**Overall structure of CRD4**. *A*, interactions of CRD4 with sugar ligands shown as a superposition of complexes αMeMan/3, Manα1-2Man/4, and Manα1-6Man/2. αMeMan/3 is shown in *cyan*, Manα1-2Man/4 monomer A in *slate*, Manα1-2Man/4 monomer B in *gray*, and Manα1-6Man/2 in *light brown*. Proteins are shown in cartoon representation. Ca^2+^ are represented as *orange spheres*. Sugar ligands are shown as sticks, with carbon atoms in the same color as the corresponding protein, oxygen atoms are *red*, and nitrogen atoms are *blue*. *B*, arrangement of CRDs in domain-swapped dimer of CRD4 (PDB ID 1egg). One CRD is shown in *cyan* except for residues 707–727, which are *blue*. The domain swap partner of this copy is shown in *light gray* except for residues 707–727, which are *dark gray*.

In all of the structures, residues from 641 to residue 761 at the C terminus are visible and this portion of the CRD adopts a similar conformation in all of the structures (Fig. 4*A*). This result is consistent with the demonstration that the truncated, minimal CRD starting at residue 640 is able to fold and bind sugars independently from the N-terminal region. The fold in the C-terminal region from residue 641 onward is similar to that of other C-type CRDs, with two helices flanking a central region of β structure. The N and C termini are paired with each other. In contrast to the results with the more extended CRD analyzed previously (29) (Fig. 4*B*), no domain swapping between CRDs in the same asymmetric unit or between symmetry-related molecules was observed in any of the crystal forms.

**Figure 5:**
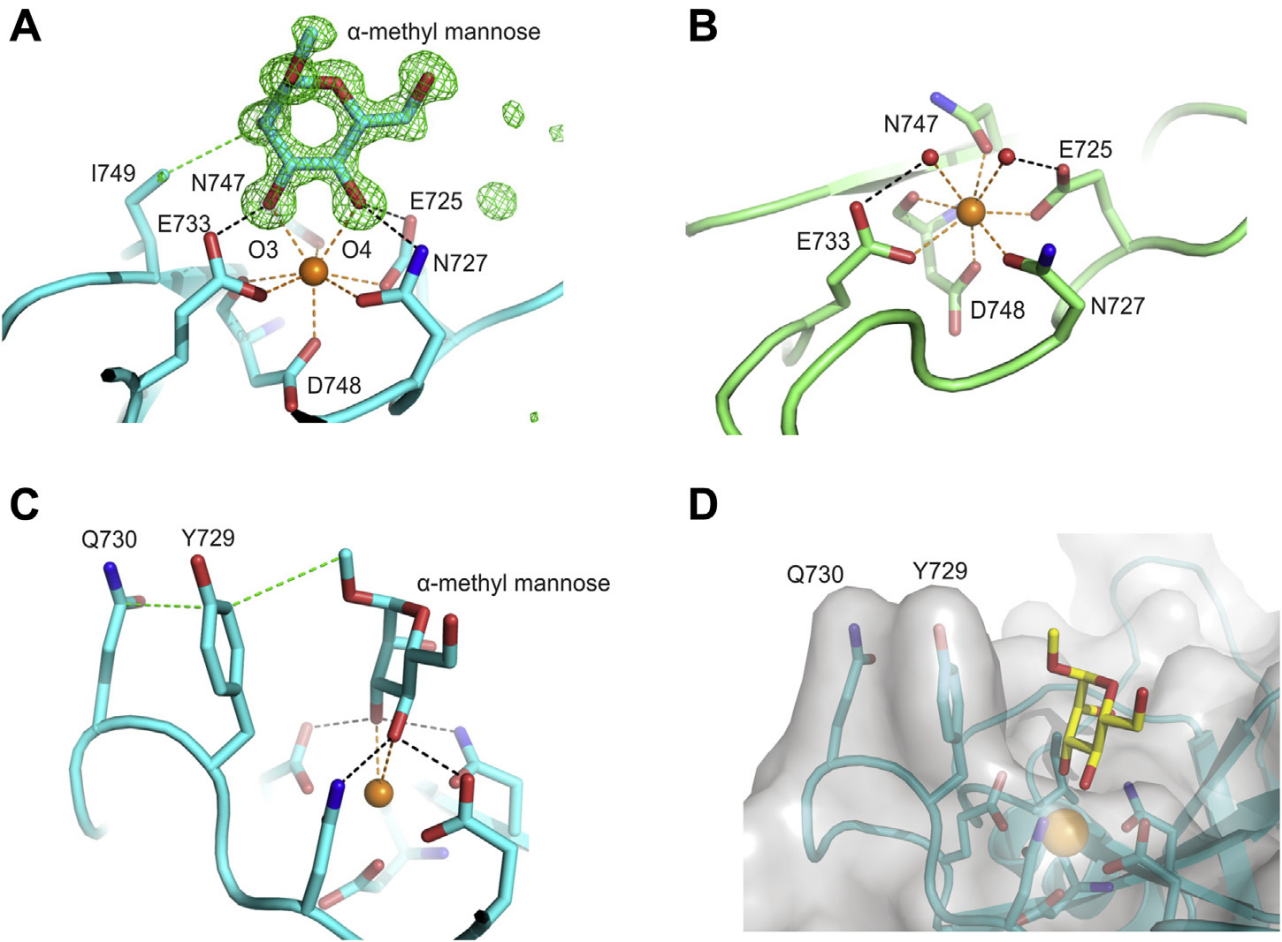
**Close-up view of the principal Ca**^**2+**^**and primary monosaccharide-binding sites in CRD4 in the presence and absence of α-methyl mannose**. *A*, primary monosaccharide-binding site of CRD4 complexed with α-methyl mannose. Fo−Fc electron density map calculated by omitting the sugar from the model, contoured at 3.0 σ, and shown as *green mesh*. *B*, principal Ca^2+^-binding site in the absence of sugar ligand as seen in native/8 copy B. *C,* Side view of the primary monosaccharide-binding site for CRD4 complexed with α-methyl mannose. *D,* Surface representation (*gray*) of the view in (C). In *panelsA*, *C*, and *D*, the model shown is taken from structure αMeMan/3. Oxygen atoms are *red*, nitrogen atoms are *blue*, and Ca^2+^ are *orange*. The protein backbone is shown in cartoon representation, and carbons of the protein or carbohydrate that are shown in stick representation are in *cyan* for αMeMan/3 and *green* for native/8 monomer B, except for *panel D*, where the carbon atoms of the carbohydrate are in *yellow*. In all figures, the black dashed lines indicate hydrogen bonds with distances <3.2 Å, the orange dashed lines represent Ca^2+^ coordination bonds <2.6 Å, and the green dashed lines represent nonpolar contacts <4.1 Å.

The binding of α-methyl mannose to CRD4 is revealed by four independent copies of this complex observed in three different crystal forms: two copies of the CRD in form αMeMan/1 and one copy of the CRD in αMeMan/2 and αMeMan/3 (Tables S3–S5). Each structure shows the same orientation of the bound sugar (Fig. 5*A*). As in other C-type CRDs, the main determinants of monosaccharide binding to CRD4 are a conserved principal Ca^2+^ and four of the five amino acid residues that surround it (Fig. 5*A*). The presence of glutamic acid and asparagine residues at positions 725 and 727 in CRD4 determines specificity for mannose, GlcNAc, and other sugars with equatorial 3- and 4-OH groups (37, 38). In a crystal form, which did not have bound sugar ligand (Native/8), the five amino acids ligating the principal Ca^2+^ had essentially identical conformations and were bonded to two water molecules that occupy the positions of the 3- and 4-OH groups of mannose (Fig. 5*B*). Comparison of these structures indicates that the binding site is largely preformed in the absence of sugar.

In addition to the Ca^2+^ ligands, the side chain of Tyr729 makes van der Waals contacts with the bound sugar (Fig. 5*C*). The position of the aromatic ring of Tyr729 is established in part by interactions with the side chain of Gln730 (Fig. 5, *C* and *D*). The protrusion of the tyrosine and adjacent glutamine residue creates a barrier adjacent to the sugar residue. In some of the crystal forms, the conformation of the side chain of Ile749 produces an additional contact with the bound sugar (Fig. 5*A*).

### Defining binding epitopes for mannose-containing oligosaccharides with competition assays

A solid-phase binding competition assay was previously used to compare affinities of CRD4 for monosaccharide ligands (23). To examine binding to oligosaccharide ligands, a modified version of the assay was developed in which the biotin-tagged CRD4 was immobilized on streptavidin-coated plates. Compared with previous studies, in which CRD4 was used directly to coat polystyrene wells, this approach gives higher binding activity because the CRDs are oriented uniformly with the sugar-binding site on the opposite site of the CRD from the biotin tether, pointing away from the surface of the well. Also, horseradish peroxidase, with its large complement of high-mannose oligosaccharides, was used as a nonradioactive alternative to ^125^I-labeled mannose-conjugated bovine serum albumin neoglycoprotein. As a test of the new assay, binding of α-methyl mannose and α-methyl fucose was compared (Table 1). The K_I_ value for the fucoside was eightfold lower than the value for the mannoside, which is consistent with the previously published values (23).

**Table 1:** Oligosaccharide binding to CRD4 determined in binding competition assays

Competing ligand	K_I_
	(*mM*)
α-Methyl mannose	0.90 ± 0.10
Manα1-2Man	0.48 ± 0.04
Manα1-3Man	0.86 ± 0.05
Manα1-4Man	0.73 ± 0.07
Manα1-6Man	0.38 ± 0.03
Manα1-6(Manα1-3)Man	0.30 ± 0.08
Man_9_	0.023 ± 0.0004
αMeFuc	0.11 ± 0.02

Results represent the average ± standard deviation for three separate assays, each performed in duplicate.

In agreement with the glycan array analysis, the competition assay indicates that the Man_9_ oligosaccharide binds with more than 40-fold higher affinity than a simple mannose residue. Examination of the binding of constituent di- and trisaccharides was undertaken to define which portions of high-mannose oligosaccharides would be likely to interact with the CRD (Table 1). The results show that the α1-3 and α1-4 linked oligosaccharides bind only slightly better than α-methyl mannose. Binding to the adjacent 3- and 4-OH groups of the reducing-end sugar in these disaccharides would be blocked, so the observed affinities suggest that interaction with the nonreducing sugar in the primary monosaccharide-binding site is not enhanced very much by further secondary interactions of the reducing-end sugar. In contrast, both the α1-2 and α1-6 linked disaccharides show more than twofold enhanced affinity. While this effect is small, it is similar to that seen for other receptors that bind high-mannose oligosaccharides (39). The enhanced affinity for the α1-2 linked oligosaccharide is consistent with the preference for glycans containing this linkage in the glycan array experiment. The affinity of the branched trisaccharide can largely be explained by the enhancement seen for the α1-6 linked disaccharide.

The enhanced affinity for the α1-2 and α1-6 linked dimannosides could reflect the fact that in these oligosaccharides both sugar residues have free 3- and 4-OH groups, suggesting that either one could bind in the primary monosaccharide-binding site. Binding could also be enhanced by the second residue making additional contacts with the CRD in an extended binding site.

### X-ray crystallography of trimmed CRD4 bound to mannose disaccharides

**Figure 6:**
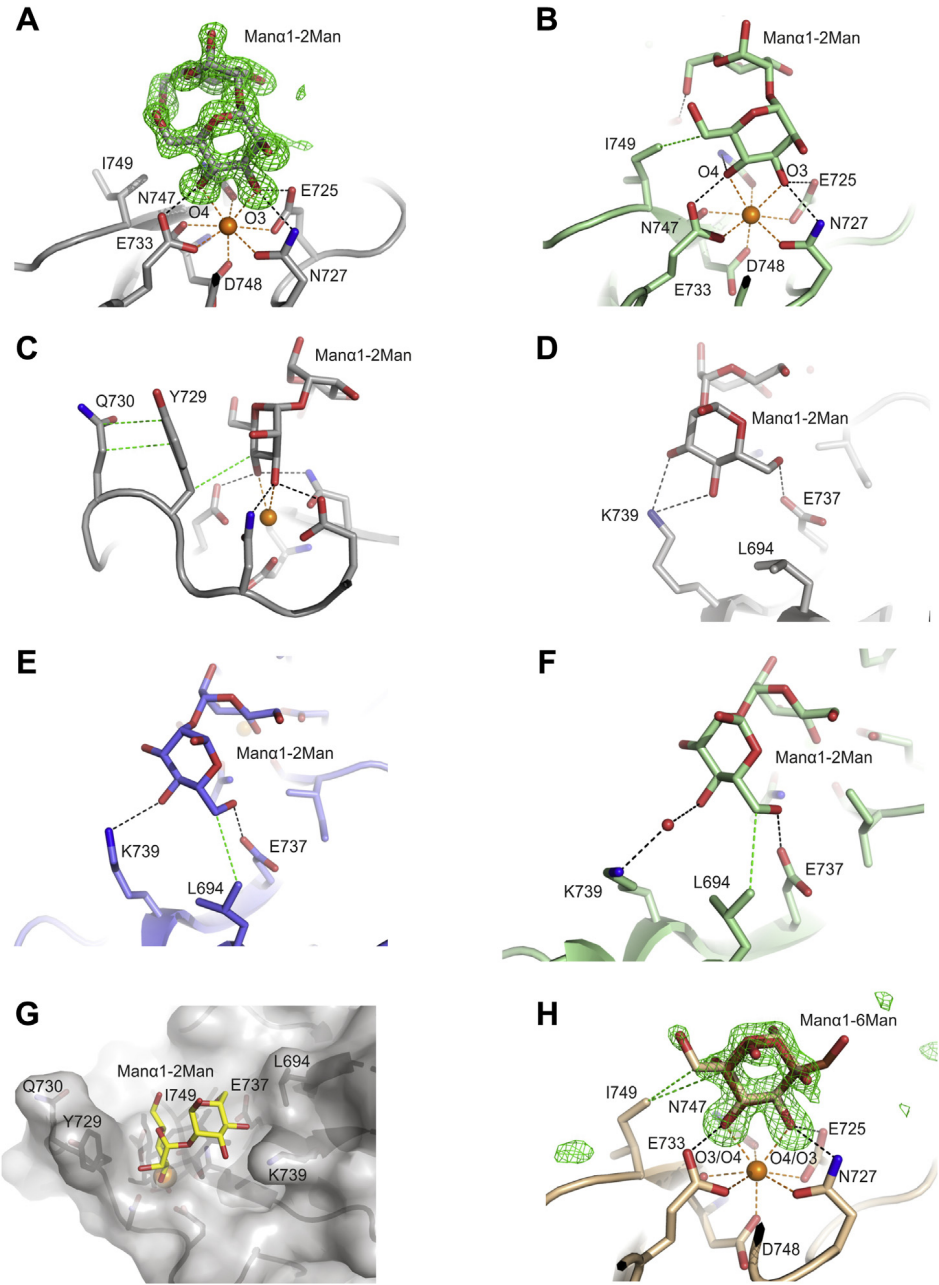
**Dimannoside binding to CRD4**. *A*, primary monosaccharide-binding site of copy B of CRD4 complexed with Manα1-2Man from structure Manα1-2Man/4. The F_o_ − F_c_ electron density map calculated by omitting the sugar from the model, contoured at 3.0 σ is shown on the model as *green mesh*. *B,* primary monosaccharide-binding site of Manα1-2Man/2. *C*, side view of the primary monosaccharide-binding site for Manα1-2Man/4, monomer B. *D*, close-up view of the interactions of the second mannose residue in the extended carbohydrate binding site of Manα1-2Man/4 monomer B. *E*, interactions of the second sugar residue in Manα1-2Man/4 monomer A. *F,* interactions of the second sugar residue in the Manα1-2Man/2 complex. *G*, Protein surface representation of the Manα1-2Man/4 complex. *H*, primary monosaccharide-binding site of CRD4 complexed with Manα1-6Man, structure Manα1-6Man/2. Colors are the same as in Figure 5, except that the carbon atoms are *gray* for copy B of the Manα1-2Man/4 complex, *light brown* for the Manα1-6Man/2 complex, *green* for Manα1-2Man/2 and *Slate* for copy A of the Manα1-2Man/4 complex and in *panel G*, the carbon atoms of the carbohydrate are in *yellow*.

Information about the mechanism of Manα1-2Man disaccharide binding to CRD4 was provided by two crystal forms that were obtained with the Manα1-2Man disaccharide (Tables S2–S5). These crystals provide three independent views of the bound sugar: one in Manα1-2Man/2 and two in Manα1-2Man/4. The interactions are largely similar among the three copies (Fig. 6, *A–C*). The nonreducing-end mannose occupies the primary monosaccharide-binding site, but it is rotated 180º compared with the α-methyl mannose (Fig. 5*A* *versus* Fig. 6, *A–C*). In some copies, the interaction with Ile749 is absent due to a difference in the side chain rotamer (Fig. 6, *A* and *B*).

The reducing-end mannose is accommodated in an extended binding site in which the 4-OH group and in some copies also the 3-OH group form hydrogen bonds with the side-chain amino group of Lys739 (Fig. 6, *D–F*). The 6-OH group forms a hydrogen bond with a carboxylate oxygen of Glu737, and in some of the structures C6 makes van der Waals contact with the side chain of Leu694. As a result of these interactions, the disaccharide is located between Tyr729 on one side and a second protrusion formed by Lys739 and Leu694 on the other (Fig. 6*G*). The results suggest that the enhanced affinity for this disaccharide results from secondary interactions in the extended binding site. The 1-OH group at the reducing end projects away from the CRD, which would allow binding to the disaccharide attached to other sugar residues in a high-mannose oligosaccharide.

In contrast to the crystals with the Manα1-2Man disaccharide, the two crystal forms with the Manα1-6Man disaccharide do not show evidence of secondary interactions. Only a single mannose residue is visible in the Manα1-6Man/2 and Manα1-6Man/5 crystals, and in each case the electron density corresponds to a mixture of two orientations (Fig. 6*H*). It is not possible to determine whether the sugar in the primary monosaccharide-binding site corresponds to the reducing or nonreducing sugar or a mixture of both. However, the enhanced affinity of this disaccharide compared with α-methyl mannose, in spite of the absence of interactions with the second sugar, suggests that either sugar can bind in the primary monosaccharide-binding site. In this case, the enhanced affinity may result from the effect of multiple binding modes (40).

The location of the principal Ca^2+^ binding site and the associated amino acid side chains that ligate the Ca^2+^ and the sugar ligands is the same in all of the structures. The positions of Tyr729 and Gln730, which form the surface projection at one side of the binding site, are also conserved. The results are again consistent with the general observation that sugar-binding sites in most C-type CRDs are preformed and do not undergo sugar-induced conformational changes. However, there are some variations in the positions of some of the nearby residues that make contacts with the sugar ligands. For example, while there is a single conformation of the side chain of Ile749 in the αMeMan/3 crystals, there are multiple conformations in the αMeMan/1 crystals. This difference is also seen in the Manα1-2Man/2 structure, where Ile749 has a different conformation from that in the Manα1-2Man/4 structure (Fig. 6, *A* and *B*). The different conformations would slightly alter the van der Waals interactions that this side chain makes with the sugar bound in the primary monosaccharide-binding site. Also, comparison of the two independent copies in the Manα1-2Man/4 structure, as well as the Manα1-2Man/2 structure, reveals that, in the extended binding site, the side chains of Lys739 and Leu694 are rearranged so that the interactions with the second sugar residue are slightly different in each case (Fig. 6, *D–F*).

### CRD4 bound to fucose-containing oligosaccharides

**Figure 7:**
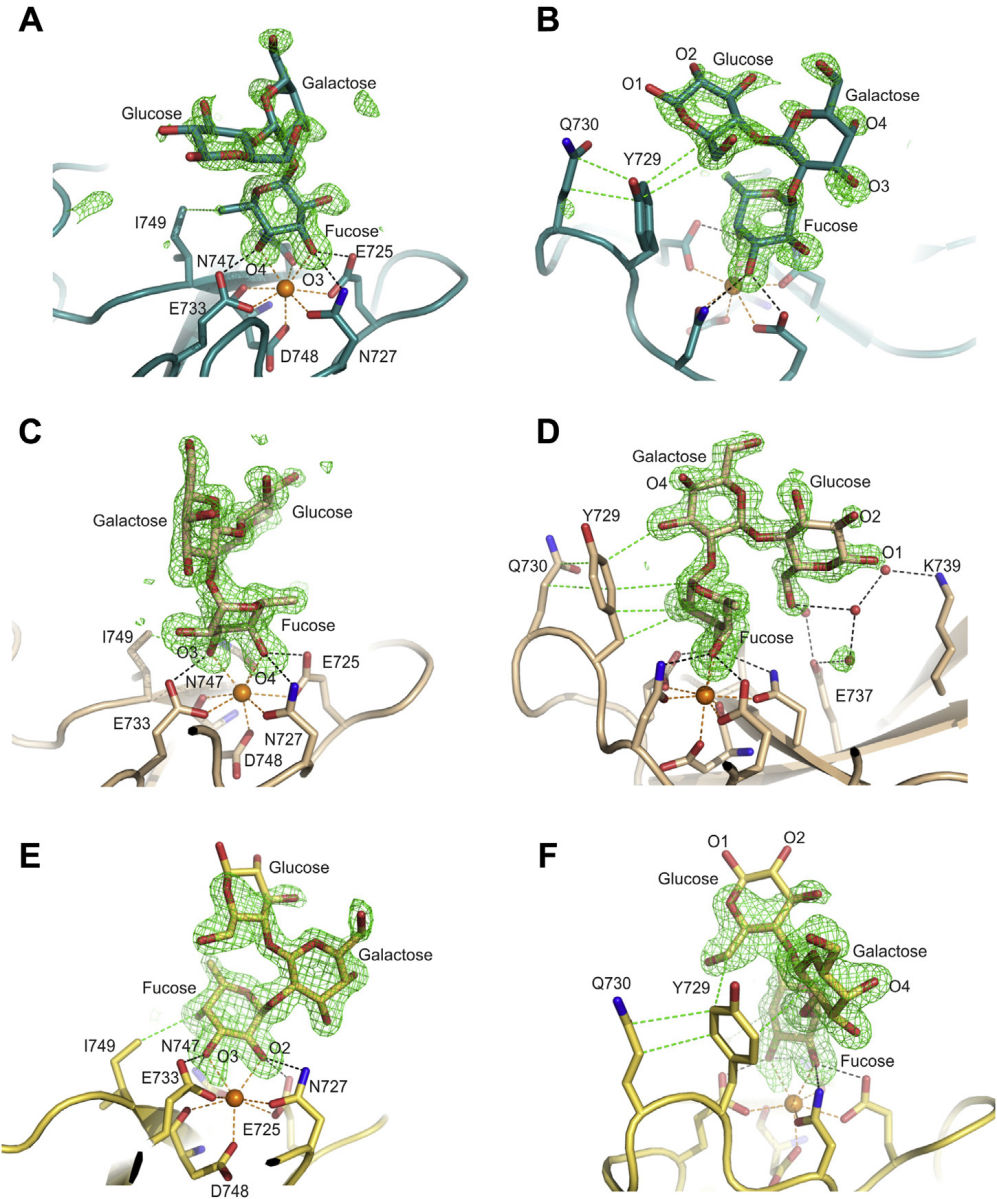
**Fucα1-2Gal binding to CRD4.***A* and *B* are from crystal form Fucα1-2Galß1-4Glc/3; *C* and *D* are based on crystal form Fucα1-2Galß1-4Glc/2; *E* and *F* are from crystal form Fucα1-2Galß1-4Glc/6; *A*, *C*, and *E* show the three different ways in which adjacent OH groups of fucose are ligated to the principal Ca^2+^. *B*, *D*, and *F* highlight interactions with Tyr729 and Gln730 as well as the positions of the 1- and 2-OH groups of glucose. Colors are the same as in Figure 5, except that the carbon atoms are *teal* for the Fucα1-2Galß1-4Glc/3 complex, *light brown* for the Fucα1-2Galß1-4Glc/2 complex, and *yellow* for Fucα1-2Galß1-4Glc/6.

Based on the glycan array results, several fucose-containing oligosaccharides that represent potential binding epitopes were screened in crystallography experiments (Tables S2–S5). Fucose in α1-2 linkage to galactose is found in glycans ranked 1, 2, 7, 18, 19, and 22 in the glycan array experiments. When the trisaccharide Fucα1-2Galβ1-4Glc was incorporated into crystals, the fucose residue was found to ligate to the principal Ca^2+^ in three different arrangements: with the 3- and 4-OH groups ligated to Ca^2+^ in two different orientations, related by a 180º rotation, or with the 2- and 3-OH groups ligated to Ca^2+^ (Fig. 7, *A–F*). In all cases, the fucose residue makes additional interactions with Ile749 and various parts of the oligosaccharide make contact with Tyr729. These contacts are possible because of some flexibility in the conformation of the Fucα1-2 linkage.

In all of the structures, the 2-OH group of the glucose residue is exposed to the solvent (Fig. 7, *B*, *D* and *F*), so that a 2-acetamido group could be accommodated in these structures, allowing binding of glycans such as 1, 7, 18, and 19 on the array, in which the glucose residue is replaced by GlcNAc. The 1-OH group is also exposed, so that CRD4 can bind to ligands displayed on the glycan array through spacers at the reducing end as well as to larger oligosaccharides. In all of the structures, the 4-OH group of galactose is exposed, so that additional sugar substituents at this position in glycans 1, 7, and 18 would be accommodated (Fig. 7, *B*, *D* and *F*). In one of the structures, the 3-OH group of galactose is exposed, potentially allowing interaction with glycans such as 18 and 22, which are extended at this position (Fig. 7*B*).

**Figure 8:**
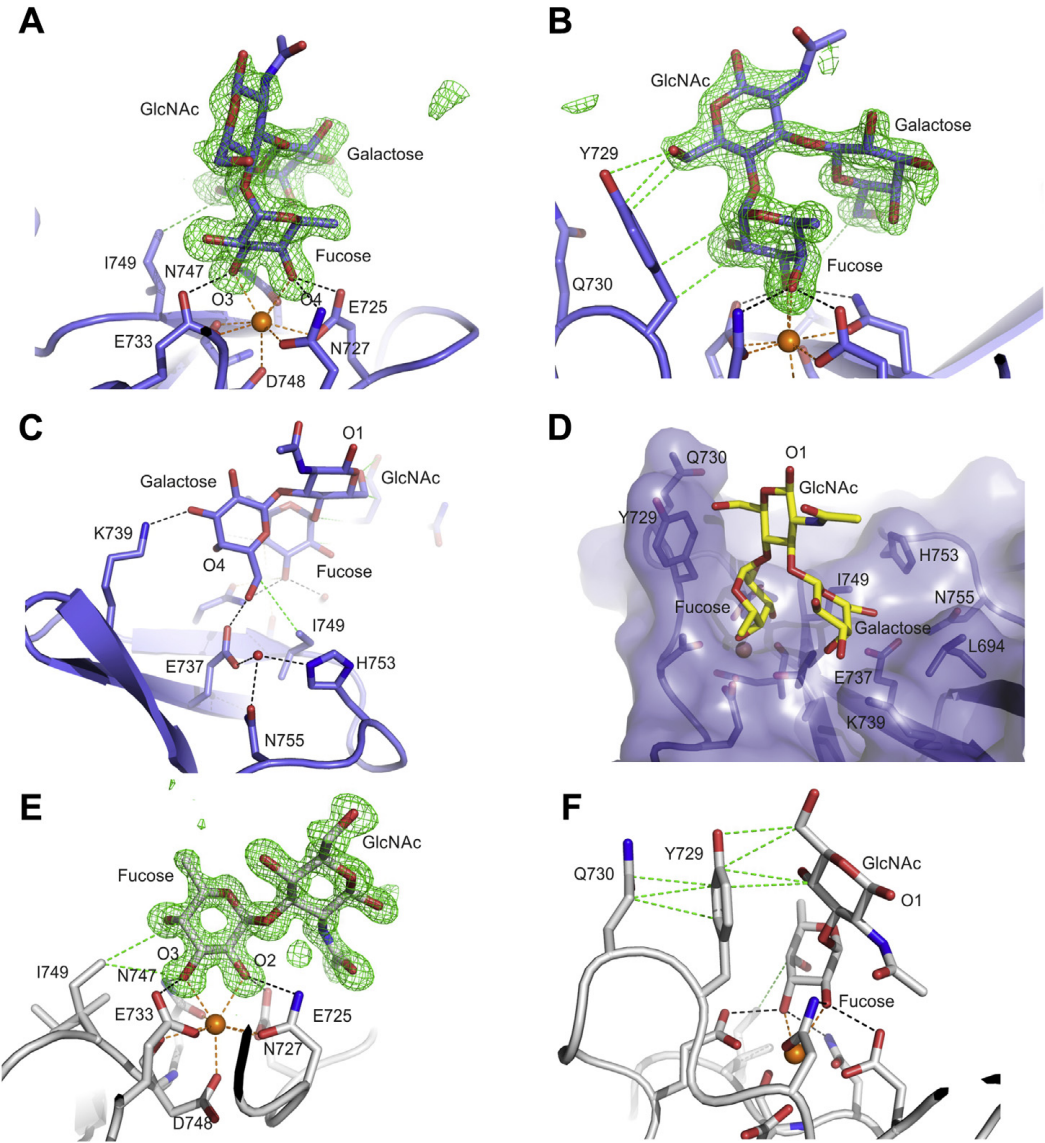
**Fucα1-3 and Fucα1-4 linked oligosaccharides binding to CRD4.***A–D*, Lewis-a trisaccharide bound to CRD4. *E–F*, Fucα1-3GlcNAc disaccharide bound to CRD4. *A* and *E* show that different OH groups in fucose interact with the principal Ca^2+^ in the two complexes. *B* and *F* show interactions with Tyr729. *C* shows interactions of CRD4 with the galactose residue of the Lewis-a trisaccharide and *D* is a surface representation of CRD-4 bound to the Lewis-a trisaccharide. Colors are the same as in Figure 5, except that the carbon atoms and surface representation are *slate* for the Lewis-a complex and *gray* for Fucα1-3GlcNAc and in *panel D*, the carbon atoms of the carbohydrate are in *yellow*.

Several of the ligands identified on the glycan array, such as glycans 6, 8, 11, 12, 20, and 21, contain the Lewis-a epitope, in which fucose is linked α1-4 and galactose in linked β1-3 to GlcNAc. A single crystal form with the Lewis-a trisaccharide was obtained (Fig. 8, *A–D*). The fucose residue in the primary binding site interacts with the principal Ca^2+^ through the 3- and 4-OH groups, as in the Fucα1-2Galß1-4Glc/2 structure with α1-2 linked fucose. There are favorable packing interactions of both the fucose and GlcNAc residues with Tyr729 and a hydrogen bond between the 3-OH group of the galactose residue and Lys739, all of which would contribute to enhanced affinity for this ligand. A 4-sulfate group on the galactose residue of the Lewis-a trisaccharide, as in structures 20 and 21, could also be accommodated. With a single negative charge distributed over three oxygens, the sulfate might form polar hydrogen bonds with Lys739 (Fig. 8*C*).

The structure of the Lewis-a complex also suggests why no oligosaccharides that contain Lewis-x structures give signals at more than 5% of the maximal values in the glycan array screening, in spite of the fact that the fucose and galactose residues in Lewis-a and Lewis-x trisaccharides have nearly identical orientations. The difference between these two structures is that the bridging GlcNAc residue is rotated by 180º. This rotation would cause a steric clash between the 2-acetamido group of GlcNAc and Tyr729 (Fig. 8*B*). The Fucα1-3GlcNAc disaccharide is a substructure of the Lewis-x trisaccharide and gives a strong signal for binding to CRD4, but a structure with the disaccharide shows that the fucose residue is in a completely different orientation (Fig. 8, *E* and *F*). In this case, binding to the principal Ca^2+^ is through the 2- and 3-OH groups of fucose, as in the Fucα1-2Galß1-4Glc/6 structure. Van der Waals interactions of the GlcNAc residue with Tyr729 would stabilize the interaction.

### Binding of GlcNAc to CRD4

**Figure 9:**
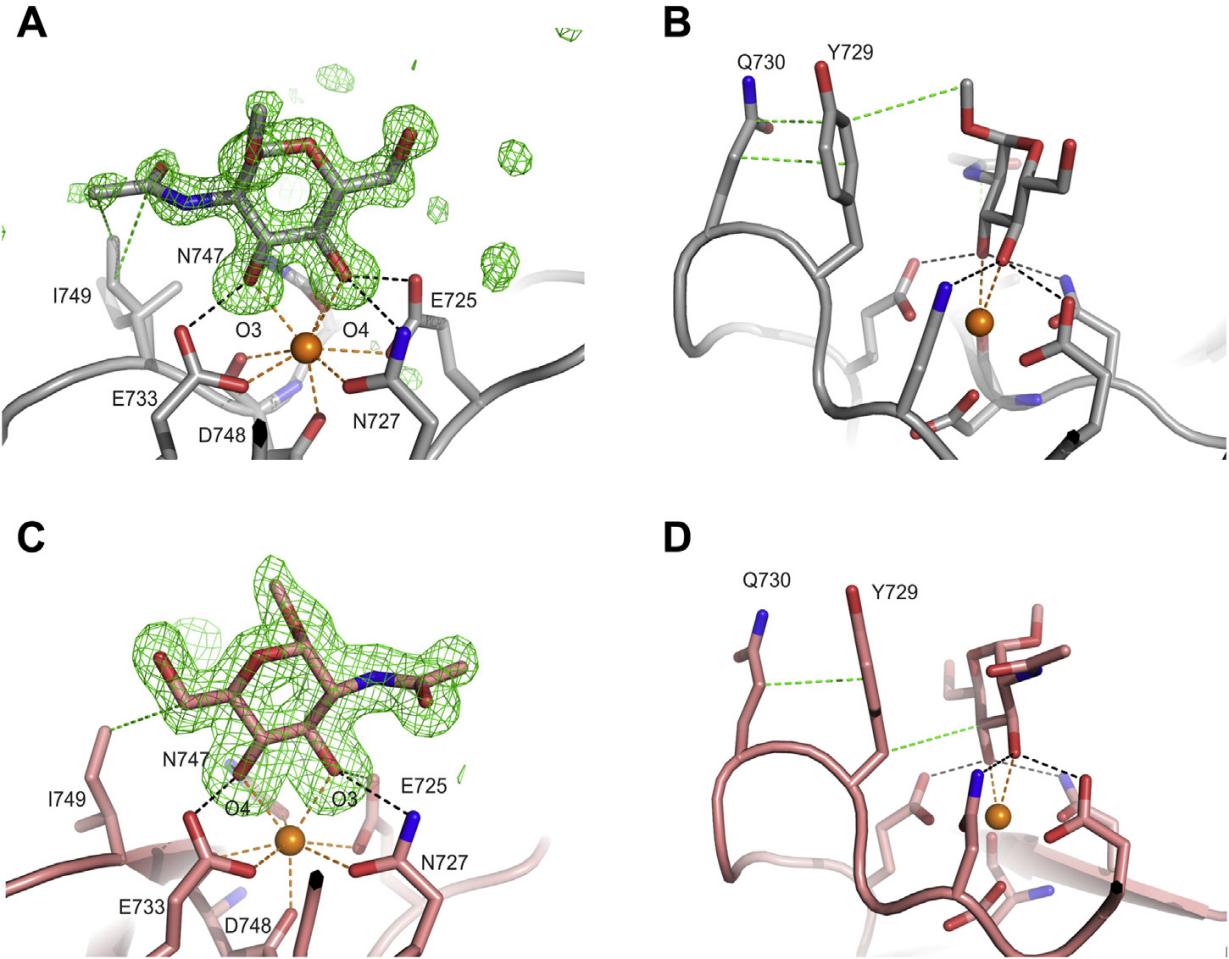
**α-Methyl GlcNAc binding to CRD4.***A* and *B*, orientation of α-methyl GlcNAc seen in crystal αMeGlcNAc/3. *C* and *D,* orientation of α-methyl GlcNAc seen in crystal αMeGlcNAc/7. Colors are the same as in Figure 5, except that the carbon atoms are *gray* for the αMeGlcNAc/3 complex and *pink* for αMeGlcNAc/7.

Competition binding assays and NMR titrations have indicated that GlcNAc binds to CRD4, albeit with weaker affinity than mannose or fucose (13, 23). In the array screening, the highest-ranked glycan with exposed terminal GlcNAc residues, ranked 23, has three terminal GlcNAc residues. Crystals prepared with α-methyl GlcNAc (Tables S2–S5) show that, like mannose, GlcNAc interacts with the principal Ca^2+^ through the 3- and 4-OH groups in two different orientations that differ by 180º (Fig. 9). Depending on the orientation, either the C6 or the acetyl group at position 2 of GlcNAc interacts with Ile749.

### Other features revealed in the crystal structures

**Figure 10:**
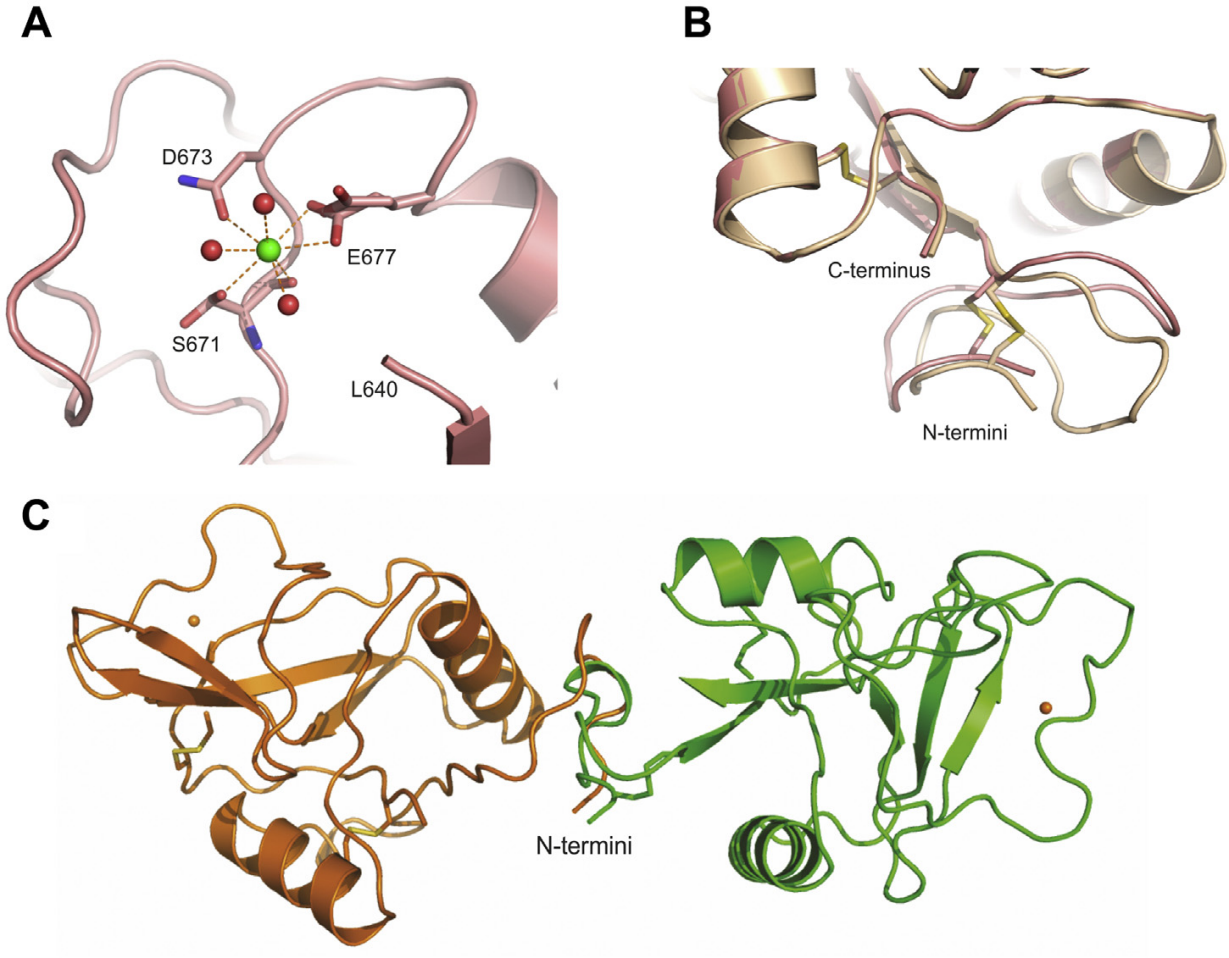
**Additional structural features of CRD4.***A*, Remote Ca^2+^ site in αMeMan/1 copy A. *B*, N-terminal regions of αMeMan/1 copy B and Manα1-6Man/2. *C*, N-terminal regions of copies A and B from the Native/8 structure superpositioned, showing the resulting changes in the orientations of the remainder of the CRDs. Protein in cartoon representation and carbons of the protein that are shown in stick representation are in *pink* for αMeMan/1, *light brown* for Manα1-6Man/2, *brown* for Native/8 copy A, and *green* for Native/8 copy B. Ca^2+^ ion is *green* for the remote site. Other atoms are colored as in Figure 5.

While the principal Ca^2+^ was present in all of the crystal forms, in four forms, αMeMan/1, Manα1-6Man/5 Manα1-2Man/4, and Lewis-a/3, anomalous scattering difference maps indicated that an additional Ca^2+^ was present at low occupancy. The position of this Ca^2+^, which is remote from the primary monosaccharide-binding site, is similar to a remote Ca^2+^ observed in dectin-2, mincle, and several other C-type CRDs (Fig. 10*A*) (41, 42). However, the position of the remote Ca^2+^ in CRD4 varies among the different crystal forms. In the αMeMan/1 crystals, the electron density map shows multiple conformations for Ser671 and Glu677. These side chains, as well as the Ca^2+^, have been modeled at 50% occupancy, with one of the partially occupied chain conformations binding to the Ca^2+^. For copy A in the Manα1-2Man/4 crystals, there appears to be Ca^2+^ at two possible sites, with each modeled at 50% occupancy. In the Manα1-6Man/5 and the Lewis-a/3 crystals, the Ca^2+^ is refined at 75% occupancy. All of the crystals were generated at 5 mM Ca^2+^, and the low occupancy suggests that this site would not have a bound Ca^2+^ under physiological conditions.

In crystal forms 2 (Manα1-6Man/2, Manα1-2Man/2, Fucα1-2Galß1-4Glc/2, and Fucα1-3GlcNAc/2), 7 (MeGlcNAc/7), and 8 (native/8), the structure of the N-terminal region, comprising residues 628–639 from the receptor, is well defined (Fig. 4*A*). This portion of the structure shows the disulfide-bonded loop characteristic of other C-type CRDs, but lacks the paired β strands often seen in this region. However, the conformation of this region differs in some of the independent copies, indicating that the N-terminal portion of the protein is flexible (Fig. 10*B*). This flexibility is also suggested by partially ordered segments that are visible in other crystal forms. For example, in copy B of the αMeMan/1 crystal form, the N-terminal disulfide-bonded loop is present, but it has a different conformation in which the χ1 angle of Cys640 differs from that seen in the Manα1-6Man/2 and Manα1-2Man/2 crystals. Additional conformations of the N-terminal region are seen in the two copies of the CRD in the unliganded crystal structure (Fig. 10*C*).

## Discussion

### Correlation with earlier NMR and mutagenesis data

The structures of CRD4 and its complexes with sugars are consistent with earlier mutagenesis and NMR data. The importance of the interaction of the sugar with Tyr729 was demonstrated by changing this residue to alanine, which caused a fivefold loss in affinity for α-methyl mannose. In the presence of CRD4, resonances for the H6, H6', and H5 protons in the NMR spectrum of α-methyl mannose are shifted and broadened (25). Mutagenesis demonstrated that these changes in the spectrum were caused by the ring current effect from the side chain of Tyr729. These results are consistent with the orientation of mannose in the primary binding site seen in the αMe-Man crystals (Fig. 5*A*), which places C5 and C6 above the tyrosine ring. Thus, in solution this orientation of α-methyl mannose appears to be preferred compared with the orientation seen in the crystals with the Manα1-2Man ligand (Fig. 6*A*).

The NMR studies also showed that α-methyl GlcNAc binding to CRD4 resulted in broadening of the H2 and O-methyl resonances, suggesting a 180º rotation compared with the binding of α-methyl mannose. Thus, the NMR results indicate that the orientation seen in Figure 9, *A* and *B* is preferred, since C2 is directly over Tyr729 in this orientation and the attached proton would be subject to a ring current effect. Perturbation of resonances in the proton NMR spectrum of the protein by titration with α-methyl GlcNAc suggested that two additional residues, Ile749 and His753, are near to the binding site for GlcNAc (23). The crystal structure confirms that the 2-acetamido group lies within 3.7 Ǻ of Ile 749 and 4.3 Ǻ of His753.

Prior studies of mannose-binding protein A and langerin revealed that fucose can bind to the principal Ca^2+^ through the 2- and 3-OH groups (43, 44). In contrast, binding of fucosylated ligands to the selectins and DC-SIGN revealed that the 3- and 4-OH groups of fucose form the coordination bonds with the principal Ca^2+^, which produces a different orientation of the hexose ring relative to mannose (45, 46).

The earlier NMR experiments showed a significant ring current effect from Tyr729 on the proton resonances of the O-methyl group of α-methyl fucose bound to CRD4, with smaller shifts seen for H1 and H5 (25). These shifts are consistent with positioning of these atoms above Tyr729. Binding of α-methyl fucose with fucose in the orientation shown in Figure 7, *E* and *F* places the O-methyl group above Tyr729.

**Figure 11:**
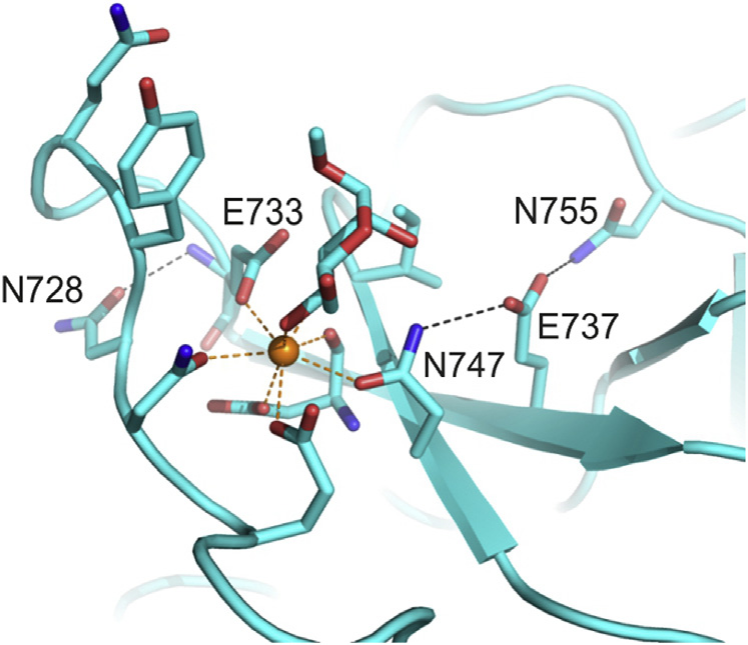
**Residues surrounding the principal Ca**^**2+**^**site in CRD4.** Interactions of additional amino acid residues with the amino acid side chains that ligate the principal Ca^2+^ to form the primary monosaccharide-binding site in αMeMan/3. A hydrogen bond links Asn728 with Ca^2+^ ligand Glu733. A network of hydrogen bonds links Asn755 and Glu737 with Ca^2+^ ligand Asn747. Carbon atoms and protein backbone, shown in cartoon representation, are cyan. Other atoms are colored as in Figure 5.

Binding of Ca^2+^ to CRD4 was investigated previously by mutagenesis studies (25). Among many residues tested for their effect on the affinity for Ca^2+^, changing either Asn728 or Glu737 to alanine decreased the affinity for Ca^2+^ by about tenfold. The side chains of these two residues make hydrogen bonds near the principal Ca^2+^-binding site. The side chain of Glu737 makes a hydrogen bond with the side chain of Asn747, which is one of the ligands for the principal Ca^2+^, and the side chain of Asn728 makes a hydrogen bond with the backbone of Glu733, which is another Ca^2+^ ligand (Fig. 11). The hydrogen bonds must contribute significantly to maintaining the position of the residues that surround the principal Ca^2+^ and bind the sugar. Less dramatic effects are seen for combinations of mutations in Asn731, Asn750, and Asn755, which form hydrogen bonds near the principal Ca^2+^ site, but not directly with the Ca^2+^ ligands. For instance, Asn755 makes a hydrogen bond with Glu737, which in turn interacts with a Ca^2+^ ligand as discussed above.

### Novel features of the sugar-binding site

The mannose-containing ligands detected on the glycan array and investigated structurally represent substructures of known microbial and mammalian ligands for the mannose receptor. A common feature of many of the microbial ligands, including yeast mannan and the capped lipo-arabinomannans on mycobacteria, are α1-2 mannose residues (47, 48). Several other receptors of the innate immune system have binding specificities that overlap with the mannose receptor, including mannose-binding protein (26), DC-SIGN (46), langerin (44, 49), and dectin-2 (41). All of these receptors share the same set of amino acids around the principal Ca^2+^, but differences in the surrounding sequences give the binding sites different shapes in each of these receptors (Fig. S2). Nevertheless, all of these receptors can accommodate the Manα1-2Man disaccharide ligand. An interesting feature of the glycan array results is that some clusters of monosaccharides bind well to CRD4 when they are simple oligosaccharides, but larger glycans that contain these same clusters do not bind as well. In some cases, this difference in binding may result from the protrusions from the surface of CRD4 near to the binding site that cause steric clashes when additional sugars are present.

The binding and structural analysis highlights several distinctive aspects of ligand binding by the mannose receptor that have not been fully appreciated. Although it is well known that the mannose receptor binds to fucose as well as to mannose (23, 24, 25), the current survey of ligands with the glycan array, and the corresponding structures with fucose-containing ligands, represents the first time that specific fucose-containing oligosaccharides have been compared as target ligands for the receptor. Binding of the sulfated forms of Lewis-a epitopes to CRD4 shows that, in addition to the well-documented interaction of the R-type CRD with sulfated sugars including ones containing sulfated Lewis-a epitopes (50), some sulfated ligand also interacts with CRD4.

More generally, the results provide a clear contrast between binding of ligands that bear Lewis-a epitopes and the Lewis-x structure, which is excluded. The absence of Lewis-x binding has been previously noted as a feature that distinguishes the mannose receptor from DC-SIGN, but the ability to bind Lewis-a epitopes has not been noted (35). The difference in binding between DC-SIGN, which preferentially binds to Lewis-x epitopes, and the mannose receptor, which preferentially binds to Lewis-a epitopes, may account for differences in the pathogens that interact with these receptors. Interaction of hosts with Lewis blood group substances on *Helicobacter pylori* has been investigated, but binding to host cells does not correlate specifically with expression of Lewis-a structures on the bacteria (51). The results highlight the need for more systematic screening of microbial pathogens against multiple receptors to identify unexplored host–pathogen interactions. It is also possible that structures such as the Lewis-a epitope may be specific markers for interaction with as yet unidentified endogenous ligands cleared by the mannose receptor.

### Conformational changes involving CRD4

Comparison of the conditions used for crystallization in these studies and in earlier work does not reveal a clear reason for variation in the behavior of CRD4, namely the lack of domain swapping in the structures reported here. The previously reported crystals were obtained at pH 8.0, whereas the crystals reported here were grown near pH 6.0 and frozen with cryoprotectants between pH 6.0 and 7.5 (Table S3) In all cases, the crystals were obtained in the presence of 5 mM CaCl_2_, and the NaCl concentration was lower in the current studies (25 *versus* 100 mM). The finding that trimming of extra sequences at the N- and C-terminal ends of CRD4 resulted in different crystal forms is consistent with the earlier suggestion that the domain swapping observed in the previous crystals was a consequence of lattice formation (29). Nevertheless, the fact that both forms can exist near physiological conditions suggests that domain swapping might occur in the intact receptor at the cell surface. It has been proposed that a monomeric form of the mannose receptor favors binding of mannose-containing ligands, while a dimeric form binds more to sulfated ligands (18). One potential explanation for this observation is that the receptor dimerizes as a consequence of domain swapping between the copies of CRD4 in two polypeptides. Dimerization would then cause both loss of mannose-binding activity and clustering of the R-type CRDs, resulting in increased affinity for sulfated ligands. As expected in this model, dimeric Fc chimeras of the extracellular domain of the receptor bound particularly well to sulfated targets on the glycan array (36). However, potential triggers for the domain swapping have not been identified.

Data from crystallography, electron microscopy, and small-angle X-ray scattering indicate that the mannose receptor and the related receptor Endo180 undergo conformational changes in endosomes correlated with release of bound ligands (17, 27). Interaction between CTLD2 and CTLD3 at acidic pH is stabilized by electrostatic interactions, and it has been suggested that these electrostatic interactions might be a driving force for conformational changes that lead to ligand release at endosomal pH (28). Interactions of CRD4 with adjacent domains may also be affected by pH. Electrostatic potential calculations show that at neutral pH, the face of CRD4 to which the sugar binds displays a net positive charge, whereas the surface on the opposite side of the CRD, including the N terminus, has a net negative charge (Fig. S3). As the pH is reduced to pH 5.0, which would correspond to the state in endosomes, negative charge around the N terminus is significantly reduced. Comparison of the different structures of CRD4 described here shows that flexibility in the N-terminal portion of the domain could accommodate changes in interactions with adjacent domains. Some of the conformation observed would dramatically shift the orientation of CRD4 compared with portions of the receptor attached at the N terminus (Fig. 10*C*).

## Methods

### Expression system engineering

Synthetic oligonucleotides used to replace restriction fragments at each end of the cDNA encoding CRD4 are shown in Fig. S1. The combined fragments were inserted into expression vector pT5T (52). The final plasmids were transfected into *E. coli* strain BL21(DE3) for expression. For the biotin-tagged protein, the bacteria were also transfected with the biotin ligase expression plasmid pBirA (53).

### Expression and purification

Luria–Bertani medium contained 50 μg/ml ampicillin, or 50 μg/ml ampicillin and 20 μg/ml chloramphenicol for the double transformants. A 200-ml overnight culture grown at 25 °C was diluted into 6 L of medium and grown at 37 °C. When the *A*_550_ reached 0.7, isopropyl-β-D-thiogalactoside at a concentration of 100 μg/ml was used for induction of protein expression. Biotin was also added to a final concentration of 12.5 μg/ml for the biotin-tagged protein. After 2.5 h at 37 °C, cells were harvested by centrifugation.

Pelleted cells were sonicated in 200 ml of 10 mM Tris-Cl, pH 7.8, centrifuged at 15,000 × *g* for 15 min, and the pellet was dissolved in 100 ml of 6 M guanidine HCl, 100 mM Tris-Cl, pH 7.0. Following addition of 2-mercaptoethanol to a final concentration of 0.01% and incubation at 4 °C for 30 min, insoluble material was removed by centrifugation at 100,000 × *g* for 30 min, and the supernatant was dialyzed against three changes of 2 L of 0.5 M NaCl, 25 mM Tris-Cl, pH 7.8, 25 mM CaCl_2_. After centrifugation at 50,000 × *g* for 30 min, renatured CRD4 was purified on a 10-ml column of mannose-conjugated Sepharose prepared by divinyl sulfone coupling (54). After washing with 10 ml of 150 mM NaCl, 25 mM Tris-Cl, pH 7.8, 25 mM CaCl_2_, bound protein was eluted with 15 1-ml aliquots of 150 mM NaCl, 25 mM Tris-Cl, pH 7.8, 2.5 mM EDTA. Fractions containing protein were identified by SDS-PAGE.

### Formation of streptavidin complex

Approximately 125 μg of biotin-tagged CRD4 in 0.5 ml of 150 mm NaCl, 25 mm Tris-Cl, pH 7.8, 2.5 mm EDTA was incubated overnight at 4 °C with 75 μg of Alexa647-labeled streptavidin (Invitrogen). CaCl_2_ (1 M) was added to bring the final concentration of Ca^2+^ to 25 mM, and the complex was repurified on a 1-ml column of mannose-Sepharose. After washing with 7 ml of 150 mm NaCl, 25 mm Tris-Cl, pH 7.8, 25 mm CaCl_2_, the column was eluted with 0.25-ml aliquots of 150 mm NaCl, 25 mm Tris-Cl, pH 7.8, 2.5 mm EDTA. Fractions containing CRD4 and streptavidin were identified by SDS–polyacrylamide gel electrophoresis, and complex formation was confirmed by gel filtration analysis on a 1 x 30 cm Superdex S200 column eluted at 0.5 ml/min with 100 mM NaCl, 10 mM Tris-Cl, pH 7.8, 2.5 mM EDTA (Fig. 2*D*).

### Screening of glycan array

Glycan arrays consisted of 601 defined glycans from the Consortium for Functional Glycomics printed on NHS-activated microarray slides using a contact printer in replicates of six (31). This version 6.2 array contains all glycans from version 5.1 except that glycans 230, 252, 473, and 546–550 in version 5.1 of the array are not present. Experiments with uncomplexed streptavidin conducted along with quality control tests with plant lectins verified that there was no background binding with this batch of slides (31). For the initial screen, CRD4 complexed with Alexa647-streptavidin at 0.4 mg/ml in 150 mm NaCl, 25 mm Tris-Cl, pH 7.8, 2.5 mM EDTA and ∼5 mM CaCl_2_ was diluted 1:2 with 150 mm NaCl, 20 mm Tris-Cl, pH 7.4, 0.05% Tween 20, 1% BSA and incubated with the slides. In a second screen, the CaCl_2_ concentration was increased by 5 mM. After binding, washes were performed with 150 mm NaCl, 20 mm Tris-Cl, pH 7.4, 2 mm CaCl_2_, 2 mm MgCl_2_ before scanning with an InnoScan 1100AL scanner. Data were processed using Mapix 8.2.5 software (Innopsys, Chicago, IL). The highest and lowest values were excluded from each set of six replicate spots before the mean and standard deviation were calculated. The glycan microarray analyses were carried out according to the guidelines proposed by the MIRAGE initiative as described in the Supporting Information (55).

### Solid-phase competition binding assays

Individual wells of streptavidin-coated 96-well assay plates (Pierce) were incubated overnight at 4 °C with 100 μl of biotin-tagged CRD4 (∼10 μg/ml) in binding buffer (150 mM NaCl, 25 mM Tris-Cl, pH 7.8, 2.5 mM CaCl_2_) containing 0.1% BSA (Cohn Fraction V, Sigma). The wells were washed three times with binding buffer, and competing sugars were added in binding buffer containing 0.1% BSA and 0.1 μg/ml horseradish peroxidase (Sigma). After the plate was incubated at 4 °C for 2 h and washed five times with binding buffer, assays were developed for 5 min at room temperature with 3,3′,5,5′-tetramethyl benzidine peroxidase substrate (BioLegend) and were quenched by addition of an equal volume of 100 mM H_2_SO_4_. A_450_ was measured in a Victor3 plate reader (PerkinElmer Life Sciences). Methyl glycosides were obtained from Carbosynth, and di- and trisaccharides were from Dextra Laboratories. Isolation of Man_9_GlcNAc_2_ oligosaccharide from soybean agglutinin has been described (41).

### Crystallization, data collection, and structure determination

Crystals of the trimmed version of CRD4 (Fig. 2*A*) were grown by hanging drop vapor diffusion at 22 °C. Diffraction data were measured at 100 K on beamline 12-2 or 12-1 at the Stanford Synchrotron Radiation Laboratory. Diffraction data were integrated with XDS and scaled with AIMLESS (56, 57). Crystallization and freezing conditions are summarized in Table S3.

Crystals were obtained in eight different unit cells, one in space group P1, one in space group P2_1_2_1_2, and six in space group P2_1_. Each data set was obtained from a single crystal, except for the crystals in space group P1, for which two crystals were needed to obtain a complete data set. Crystallographic data statistics are summarized in Table S4.

The structures were solved by molecular replacement. The first structure solved was the P1 form (α-methyl mannose/1, Tables S3–S5). The model used for molecular replacement was constructed from PDB entry 1EGG by taking residues 628–701 and 735–727 from protein chain A and residues 707–727 from the symmetry-related molecule A; the latter peptide represents the dimer-swapped loops and Ca^2+^ of the principal site. A native Patterson synthesis had a large nonorigin peak (49.5% relative to the origin) at (0 0.5 0.5), indicating translational noncrystallographic symmetry, and the molecular replacement solution gave two monomers in the asymmetric unit. A phased anomalous difference map showed peaks >20 σ at the Ca^2+^ in the principal sites of the two monomers. Another peak, at 8 σ, was visible only in one copy (chain A). Further refinement and electron density maps indicated that this remote Ca^2+^ is present at an occupancy of 0.5. The side chains of chain A residues Ser 671 and Glu 677, which coordinate this Ca^2+^, had multiple conformations in which one conformation binds the Ca^2+^.

The two P2_1_ data sets of the α-methyl mannose complexes (αMeMan/2 and αMeMan/3) were solved by molecular replacement using chain B of the partially refined model of the P1 cell. In these data sets there is one monomer in the asymmetric unit. The N-terminal β strand is disordered up to residue Leu 640 in αMeMan/3 and is partially present in αMeMan/2. In this region of αMeMan/2, the gap from residue 635 to residue 638 is too long for two missing residues. One explanation for this long gap is that these residues have multiple conformations, and the final model contains part of one conformation before the gap and part of the other after the gap. We therefore assigned occupancies of 0.5 and distinct alternate conformations to residues 634–635 before the gap and residues 638–640 after it.

The data from the complex of CRD4 with Manα1-2-Man (Manα1-2Man/2) and with Manα1-6Man (Manα1-6Man/2) had the same symmetry and unit cell as one of the complexes of CRD4 with α-methyl mannose (αMeMan/2), so they were initially refined with rigid body using the partially refined structure of the α-methyl mannose complex. The initial model included the protein and the principal Ca^2+^. Similar reflections were chosen for R_free_. There are no missing residues in the model for the protein in both structures. In Manα1-6Man/2, only one mannose residue of the sugar is visible in the primary monosaccharide-binding site, and this mannose residue has an alternate conformation (0.75:0.25) with ∼180° rotation of the sugar, swapping the position of O3 and O4 (Fig. 6*H*).

Data for the Manα1-2Man/4 complex showed translational noncrystallographic symmetry (largest native Patterson peak at 0.5 0.0 0.5; 29.0% relative to the origin) and was solved by molecular replacement using the partially refined high-resolution CRD4 complex with α-methyl mannose (MeMan/3). A phased anomalous difference map indicated the presence of the principal Ca^2+^ site in both monomers (>18 σ) and a low occupancy Ca^2+^ (∼9 σ) in a remote site of monomer A. The peak was slightly elongated, and the final model includes two positions for this remote Ca^2+^, with each Ca^2+^ and the water molecules bound to it modeled as alternate conformations. Manα1-2Man was visible in the two monomers.

The Manα1-6Man/5 complex was solved by molecular replacement using the partially refined structure of the αMeMan/3 complex. The initial model included the protein and the principal Ca^2+^. An anomalous difference map indicated the presence of the principal site Ca^2+^ (>20 σ) and a remote Ca^2+^ (∼10 σ) that was refined at 0.75 occupancy. As seen in the structure of Manα1-6Man/2, the model for Manα1-6Man/5 also includes only one mannose moiety of the sugar, which has alternate conformations (occupancies of 0.75 and 0.25) related by a ∼180° rotation that swaps the positions of O3 and O4.

Lewis-a/3, αMeGlcNAc/3, and Fucα1-2Galß1-4Glc/3 complexes were refined starting from the refined structure of αMeMan/3. The initial model included the protein and the principal Ca^2+^. The same subset of reflections were chosen for R_free_. For Fucα1-2Galß1-4Glc/3, an anomalous difference map indicated the presence of the principal Ca^2+^ (>25 σ) and a remote Ca^2+^ (∼8 σ) that was refined at 0.75 occupancy.

Fucα1-2Galß1-4Glc/2 and Fucα1-3GlcNAc/2 complexes were refined starting from using the refined structure Manα1-2Man/2. The initial model included the protein and the principal Ca^2+^. The same subset of reflections were chosen for R_free_.

Fucα1-2Galß1-4Glc/6, αMeGlcNAc/7, and Native/8 complexes were solved by molecular replacement using the refined structure of the αMeMan/3 complex. The initial model included the protein and the principal Ca^2+^. Molecular replacement for the Fucα1-2Galß1-4Glc/6 and αMeGlcNAc/7 complexes showed one monomer per asymmetric unit, and the solution of the Native/8 structure showed two copies of the protein per asymmetric unit. In data set Native/8, the sugar that was present in the crystallization and freezing conditions, Man_9_GlcNAc_2_, is not visible for either copy. Instead of the sugar, there are two water molecules binding the calcium ion. The density indicated that the N-terminal of the protein, which was missing in the model used for molecular replacement, could be added. Monomers A and B show different conformations in this region.

Model building and refinement were performed with Coot and PHENIX (58, 59). Refinement included individual positional, isotropic temperature factor refinement of the water molecules and anisotropic temperature factor for the protein, Ca^2+^, and sugar except for the structure of Fucα1-2Galß1-4Glc/6, αMeGlcNAc/7, and Native/8, for which Translation-Libration-Screw refinement was employed instead of using anisotropic temperature factors. Refinement statistics are shown in Table S5.

### Electrostatics calculations

Electrostatics calculations were performed with APBS (60) and PDB2PQR (61).

## Data availability

The atomic coordinates and structure factors, with codes 7JUB, 7JUC, 7JUD, 7JUE, 7JUF, 7JUG, 7JUH, 7L61, 7L62, 7L63, 7L64, 7L65, 7L66, 7L67, 7L68 as indicated in Table S5, have been deposited in the Protein Data Bank (http://wwpdb.org/). All other data are contained within the article and Supporting Information.

## Supporting information

This article contains supporting information.

## Conflict of interest

The authors declare that they have no conflicts of interest with the contents of this article.
