# Supplementary material for: Structural analysis of carbohydrate binding by the macrophage mannose receptor CD206
Source: J Biol Chem. 2021 Feb 2;296:100368. doi: 10.1016/j.jbc.2021.100368 (PMC7949135; doi:10.1016/j.jbc.2021.100368)
Supplement: Figures and Tables [file mmc1.pdf]

Supporting Information

Structural analysis of carbohydrate binding by the macrophage mannose receptor CD206

Hadar Feinberg, Sabine A. F. Jégouzo, Yi Lasanajak, David F. Smith, Kurt Drickamer, William I. Weis, and Maureen E.Taylor

Table S1. Glycan array analysis of mannose receptor CRD4 binding on Consortium for Functional Glycomics array, version 6.2 screened at ~2.5 mM $CaCl_2$.

Table S2. Glycan array analysis of mannose receptor CRD4 binding on Consortium for Functional Glycomics array, version 6.2 screened at ~7.5 mM $CaCl_2$.

Table S3. Crystallization conditions.

Table S4. Crystallographic data statistics.

Table S5. Crystallographic refinement statistics.

Figure S1. Modification of the ends of CRD4 expression vector.

Figure S2. Comparison of mannose-binding sites in C-type CRDs.

Figure S3. Electrostatic potential surface of CRD4.

MIRAGE form. Glycan array screening information.

Table S1. Glycan array analysis of mannose receptor CRD4 binding on Consortium for Functional Glycomics array version 6.2 screened at ~2.5 mM $CaCl_2$.

Rank	Glycan Number	Structure	Average RFU	Standard deviation
1	119	Galα1-4(Fucα1-2)Galβ1-4GlcNAcβ-Sp8	49648	2540
2	78	Fucα1-2Galβ-Sp8	48051	1258
3	207	Manα1-2Manα1-2Manα1-3Manα-Sp9	40445	9639
4	79	Fucα1-3GlcNAcβ-Sp8	28819	7297
5	214	Manα1-2Manα1-2Manα1-6(Manα1-3)Manα-Sp9	22765	1073
6	129	Galβ1-3(Fucα1-4)GlcNAc-Sp8	15887	1492
7	93	GalNAcα1-4(Fucα1-2)Galβ1-4GlcNAcβ-Sp8	14980	2464
8	130	Fucα1-4(Galβ1-3)GlcNAcβ-Sp8	14965	1098
9	80	Fucα1-4GlcNAcβ-Sp8	14957	358
10	313	Manα1-2Manα1-6(Manα1-3)Manα1-6(Manα1-2Manα1-2Manα1-3)Manα-Sp9	11792	342
11	128	Galβ1-3(Fucα1-4)GlcNAc-Sp0	11375	3517
12	126	Galβ1-3(Fucα1-4)GlcNAcβ1-3Galβ1-4(Fucα1-3)GlcNAcβ-Sp0	10604	249
13	314	Manα1-2Manα1-6(Manα1-2Manα1-3)Manα1-6(Manα1-2Manα1-2Manα1-3)Manα-Sp9	10503	2585
14	7	Fucα-Sp9	9493	177
15	215	Manα1-6(Manα1-3)Manα1-6(Manα1-2Manα1-3)Manβ1-4GlcNAcβ1-4GlcNAcβ-Sp12	9388	225
16	202	GlcAβ-Sp8	8999	242
17	208	Manα1-2Manα1-6(Manα1-2Manα1-3)Manα-Sp9	8510	407
18	60	Fucα1-2Galβ1-3(Fucα1-4)GlcNAcβ-Sp8	8065	1944
19	537	Fucα1-2Galβ1-4GlcNAcβ1-3Galβ1-4GlcNAcβ1-2Manα1-6(Fucα1-2Galβ1-4GlcNAcβ1-3Galβ1-4GlcNAcβ1-2Manα1-3)Manβ1-4GlcNAcβ1-4GlcNAcβ-Sp24	7692	421
20	28	(3S)Galβ1-3(Fucα1-4)GlcNAcβ-Sp8	7235	333
21	488	(3S)Galβ1-3(Fucα1-4)GlcNAcβ-Sp0	6925	526
22	90	GalNAcα1-3(Fucα1-2)Galβ-Sp18	6618	272
23	471	GlcNAcβ1-6(GlcNAcβ1-2)Manα1-6(GlcNAcβ1-2Manα1-3)Manβ1-4GlcNAcβ1-4(Fucα1-6)GlcNAcβ-Sp24	5020	156
24	470	Fucα1-2Galβ1-3(Fucα1-4)GlcNAcβ1-2Manα1-6(Fucα1-2Galβ1-3(Fucα1-4)GlcNAcβ1-2Manα1-3)Manβ1-4GlcNAcβ1-4(Fucα1-6)GlcNAcβ1-4(Fucα1-6)GlcNAcβ-Sp19	4838	300
25	76	Fucα1-2Galβ1-4GlcNAcβ-Sp8	4689	183
26	597	Neu5Acα2-6Galβ1-4GlcNAcβ1-3Galβ1-4GlcNAcβ1-3Galβ1-4GlcNAcβ1-2Manα1-6(Neu5Acα2-6Galβ1-4GlcNAcβ1-3Galβ1-4GlcNAcβ1-3Galβ1-4GlcNAcβ1-2Manα1-3)Manβ1-4GlcNAcβ1-4GlcNAcβ-Sp12	4542	167
27	598	Neu5Acα2-3Galβ1-4GlcNAcβ1-3Galβ1-4GlcNAcβ1-3Galβ1-4GlcNAcβ1-2Manα1-6(Neu5Acα2-3Galβ1-4GlcNAcβ1-3Galβ1-4GlcNAcβ1-3Galβ1-4GlcNAcβ1-2Manα1-3)Manβ1-4GlcNAcβ1-4GlcNAcβ-Sp12	4350	277
28	540	Galβ1-4GlcNAcβ1-3Galβ1-4GlcNAcβ1-3Galβ1-4GlcNAcβ1-2Manα1-6(Galβ1-4GlcNAcβ1-3Galβ1-4GlcNAcβ1-3Galβ1-4GlcNAcβ1-2Manα1-3)Manβ1-4GlcNAcβ1-4GlcNAcβ-Sp12	4328	241
29	217	Manβ1-4GlcNAcβ-Sp0	3892	247
30	188	GlcNAcβ1-4Galβ1-4GlcNAcβ-Sp8	3874	447
31	275	Galβ1-3(Fucα1-4)GlcNAcβ1-3Galβ1-3(Fucα1-4)GlcNAcβ-Sp0	3748	662
32	503	GlcNAcβ1-6(GlcNAcβ1-2)Manα1-6(GlcNAcβ1-4)(GlcNAcβ1-4(GlcNAcβ1-2)Manα1-3)Manβ1-4GlcNAcβ1-4(Fucα1-6)GlcNAc-Sp21	3629	125
33	535	GlcNAcβ1-3Galβ1-4GlcNAcβ1-2Manα1-6(GlcNAcβ1-3Galβ1-4GlcNAcβ1-2Manα1-3)Manβ1-4GlcNAcβ1-4GlcNAcβ-Sp12	3606	474
34	418	GlcNAcβ1-2(GlcNAcβ1-6)Manα1-6(GlcNAcβ1-2Manα1-3)Manβ1-4GlcNAcβ1-4GlcNAcβ-Sp19	3598	72
35	358	Fucα1-2Galβ1-4GlcNAcβ1-2Manα1-6(Fucα1-2Galβ1-4GlcNAcβ1-2Manα1-3)Manβ1-4GlcNAcβ1-4GlcNAcβ-Sp20	3544	88
36	534	Galβ1-3GalNAcβ1-3Gal-Sp21	3200	876
37	213	Manα1-6(Manα1-3)Manα-Sp9	3186	37
38	538	GlcNAcβ1-3Galβ1-4GlcNAcβ1-3Galβ1-4GlcNAcβ1-2Manα1-6(GlcNAcβ1-3Galβ1-4GlcNAcβ1-3Galβ1-4GlcNAcβ1-2Manα1-3)Manβ1-4GlcNAcβ1-4GlcNAcβ-Sp12	2994	111
39	209	Manα1-2Manα1-3Manα-Sp9	2979	263
40	382	Galβ1-4(Fucα1-3)GlcNAcβ1-6(Fucα1-4(Fucα1-2Galβ1-3)GlcNAcβ1-3)Galβ1-4Glc-Sp21	2923	259
41	536	GlcNAcβ1-3Galβ1-4GlcNAcβ1-2Manα1-6(GlcNAcβ1-3Galβ1-4GlcNAcβ1-2Manα1-3)Manβ1-4GlcNAcβ1-4GlcNAcβ-Sp25	2784	1223
42	81	Fucβ1-3GlcNAcβ-Sp8	2684	133
43	297	Neu5Acα2-3Galβ1-4(Fucα1-3)GlcNAcβ1-6(Galβ1-3)GalNAcα-Sp14	2674	113
44	73	Fucα1-2Galβ1-4GlcNAcβ1-3Galβ1-4GlcNAcβ-Sp0	2648	247
45	75	Fucα1-2Galβ1-4GlcNAcβ-Sp0	2608	203
46	599	Neu5Acα2-6Galβ1-4GlcNAcβ1-3Galβ1-4GlcNAcβ1-2Manα1-6(Neu5Acα2-6Galβ1-4GlcNAcβ1-3Galβ1-4GlcNAcβ1-2Manα1-3)Manβ1-4GlcNAcβ1-4GlcNAcβ-Sp12	2501	775
47	102	Galα1-3(Fucα1-2)Galβ1-3GlcNAcβ-Sp8	2458	156
48	247	Fucα1-2(6S)Galβ1-4Glcβ-Sp0	2403	55
49	51	Manα1-6(Manα1-3)Manβ1-4GlcNAcβ1-4GlcNAcβ-Sp13	2400	136

50	95	GalNAcβ1-3(Fucα1-2)Galβ-Sp8	2395	215
51	187	GlcNAcβ1-6(GlcNAcβ1-4)GalNAcα-Sp8	2390	99
52	21	GlcNAcβ1-6(GlcNAcβ1-4)(GlcNAcβ1-3)GlcNAc-Sp8	2278	415
53	600	GlcNAcβ1-3Fucα-Sp21	2252	220
54	107	Galα1-3(Fucα1-2)Galβ-Sp8	2214	47
55	248	Neu5Acα2-3Galβ1-3GlcNAcβ-Sp0	2131	128
56	346	Galβ1-4GlcNAcβ1-2Manα1-3Manβ1-4GlcNAcβ1-4GlcNAc-Sp12	2127	115
57	173	GlcNAcα1-3Galβ1-4GlcNAcβ-Sp8	2116	131
58	89	GalNAcα1-3(Fucα1-2)Galβ-Sp8	2061	254
59	53	GlcNAcβ1-2Manα1-6(GlcNAcβ1-2Manα1-3)Manβ1-4GlcNAcβ1-4GlcNAcβ-Sp13	2041	269
60	111	Galα1-3GalNAcα-Sp16	2007	402
61	178	GlcNAcβ1-6(GlcNAcβ1-3)Galβ1-4GlcNAcβ-Sp8	1988	82
62	469	Fucα1-2Galβ1-4(Fucα1-3)GlcNAcβ1-2Manα1-6(Fucα1-2Galβ1-4(Fucα1-3)GlcNAcβ1-2Manα1-3)Manβ1-4GlcNAcβ1-4(Fucα1-6)GlcNAcβ-Sp24	1930	93
63	480	Manα1-6(Manα1-3)Manβ1-4GlcNAcβ1-4(Fucα1-6)GlcNAcβ-Sp19	1897	93
64	594	Neu5Acα2-3Galβ1-4GlcNAcβ1-3Galβ1-4GlcNAcβ1-2Manα1-6(Neu5Acα2-3Galβ1-4GlcNAcβ1-3Galβ1-4GlcNAcβ1-2Manα1-3)Manβ1-4GlcNAcβ1-4GlcNAcβ-Sp12	1895	96
65	77	Fucα1-2Galβ1-4Glcβ-Sp0	1832	593
66	50	Manα1-6(Manα1-3)Manβ1-4GlcNAcβ1-4GlcNAcβ-Sp12	1762	619
67	567	GlcNAcβ1-3Galβ1-4GlcNAcβ1-3Galβ1-4GlcNAcβ1-2Manα1-6(GlcNAcβ1-3Galβ1-4GlcNAcβ1-3Galβ1-4GlcNAcβ1-2Manα1-3)Manβ1-4GlcNAcβ1-4(Fucα1-6)GlcNAcβ-Sp24	1736	73
68	321	Galβ1-3GlcNAcβ1-2Manα1-6(Galβ1-3GlcNAcβ1-2Manα1-3)Manβ1-4GlcNAcβ1-4GlcNAcβ-Sp19	1715	110
69	83	GalNAcα1-3(Fucα1-2)Galβ1-4(Fucα1-3)GlcNAcβ-Sp0	1695	40
70	74	Fucα1-2Galβ1-4GlcNAcβ1-3Galβ1-4GlcNAcβ1-3Galβ1-4GlcNAcβ-Sp0	1659	475
71	446	Fucα1-2Galβ1-4GlcNAcβ1-6(Fucα1-2Galβ1-4GlcNAcβ1-3)GalNAc-Sp14	1631	34
72	216	Manα1-6(Manα1-3)Manα1-6(Manα1-3)Manβ1-4GlcNAcβ1-4 GlcNAcβ-Sp12	1618	28
73	108	Galα1-3(Fucα1-2)Galβ-Sp18	1609	51
74	176	GlcNAcβ1-6(GlcNAcβ1-3)GalNAcα-Sp8	1608	28
75	385	GlcNAcβ1-2Manα1-6(GlcNAcβ1-4(GlcNAcβ1-2)Manα1-3)Manβ1-4GlcNAcβ1-4GlcNAc-Sp21	1597	257
76	312	Manα1-6(Manα1-3)Manα1-6(Manα1-3)Manβ-Sp10	1595	184
77	237	Neu5Acα2-3Galβ1-3(6S)GlcNAc-Sp8	1574	89
78	182	GlcNAcβ1-3Galβ1-4GlcNAcβ-Sp0	1553	116
79	220	Fucα1-2(6S)Galβ1-4GlcNAcβ-Sp0	1508	382
80	531	GlcNAcβ1-3Galβ1-4GlcNAcβ1-6(GlcNAcβ1-3)Galβ1-4GlcNAc-Sp0	1464	279
81	308	GlcNAcβ1-4GlcNAcβ-Sp10	1457	44
82	367	GalNAcα1-3(Fucα1-2)Galβ1-4GlcNAcβ1-2Manα1-6(GalNAcα1-3(Fucα1-2)Galβ1-4GlcNAcβ1-2Manα1-3)Manβ1-4GlcNAcβ1-4GlcNAcβ-Sp20	1447	32
83	52	GlcNAcβ1-2Manα1-6(GlcNAcβ1-2Manα1-3)Manβ1-4GlcNAcβ1-4GlcNAcβ-Sp12	1422	488
84	183	GlcNAcβ1-3Galβ1-4GlcNAcβ-Sp8	1419	303
85	555	GlcNAcβ1-3Galβ1-4GlcNAcβ1-3Galβ1-4GlcNAcβ1-3Galβ1-4GlcNAcβ1-3Galβ1-4GlcNAcβ1-2Manα1-6(GlcNAcβ1-3Galβ1-4GlcNAcβ1-3Galβ1-4GlcNAcβ1-3Galβ1-4GlcNAcβ1-3Galβ1-4GlcNAcβ1-2Manα1-3)Manβ1-4GlcNAcβ1-4GlcNAcβ-Sp25	1398	268
86	104	Galα1-3(Fucα1-2)Galβ1-4(Fucα1-3)GlcNAcβ-Sp8	1371	63
87	17	GlcNAcβ-Sp8	1366	33
88	290	Galβ1-4(Fucα1-3)GlcNAcβ1-3Galβ1-3(Fucα1-4)GlcNAcβ-Sp0	1356	78
89	360	Galα1-3Galβ1-4GlcNAcβ1-2Manα1-6(Galα1-3Galβ1-4GlcNAcβ1-2Manα1-3)Manβ1-4GlcNAcβ1-4GlcNAcβ-Sp20	1327	12
90	84	(3S)Galβ1-4(Fucα1-3)Glcβ-Sp0	1296	332
91	191	GlcNAcβ1-4GlcNAcβ1-4GlcNAcβ-Sp8	1267	79
92	349	GlcNAcβ1-2Manα1-6(GlcNAcβ1-2Manα1-3)Manβ1-4GlcNAcβ1-4(Fucα1-6)GlcNAcβ-Sp22	1245	102
93	400	Galα1-4Galβ1-3GlcNAcβ1-2Manα1-6(Galα1-4Galβ1-3GlcNAcβ1-2Manα1-3)Manβ1-4GlcNAcβ1-4GlcNAcβ-Sp19	1241	21
94	549	GlcNAcβ1-3Galβ1-4GlcNAcβ1-6(GlcNAcβ1-3Galβ1-4GlcNAcβ1-2)Manα1-6(GlcNAcβ1-3Galβ1-4GlcNAcβ1-2Man a1-3)Manβ1-4GlcNAcβ1-4GlcNAc-Sp24	1225	67
95	368	Galα1-3(Fucα1-2)Galβ1-4GlcNAcβ1-2Manα1-6(Galα1-3(Fucα1-2)Galβ1-4GlcNAcβ1-2Manα1-3)Manβ1-4GlcNAcβ1-4GlcNAcβ-Sp20	1220	54
96	348	Manα1-6(Galβ1-4GlcNAcβ1-2Manα1-3)Manβ1-4GlcNAcβ1-4GlcNAcβ-Sp12	1219	48
97	486	Galβ1-3(Fucα1-4)GlcNAcβ1-6GalNAcα-Sp14	1206	512
98	88	GlcNAcβ1-3Galβ1-3GalNAcα-Sp8	1179	66
99	194	GlcNAcβ1-6Galβ1-4GlcNAcβ-Sp8	1173	34
100	467	Glcα1-4Glcα1-4Glcα1-4Glcβ-Sp10	1172	51
101	47	(6S)GlcNAcβ-Sp8	1168	73
102	61	Fucα1-2Galβ1-3GalNAcα-Sp8	1165	88
103	71	Fucα1-2Galβ1-4(Fucα1-3)GlcNAcβ-Sp0	1139	14
104	451	GalNAcα1-3(Fucα1-2)Galβ1-4GlcNAcβ1-2Manα1-6(GalNAcα1-3(Fucα1-2)Galβ1-4GlcNAcβ1-2Manα1-3)Manβ1-4GlcNAcβ1-4(Fucα1-6)GlcNAcβ-Sp22	1135	64
105	347	Galβ1-4GlcNAcβ1-2Manα1-6Manβ1-4GlcNAcβ1-4GlcNAc-Sp12	1124	139
106	466	Glcα1-6Glcα1-6Glcα1-6Glcβ-Sp10	1110	110
107	389	GalNAcβ1-4(Neu5Acα2-3)Galβ1-4GlcNAcβ1-3GalNAcα-Sp14	1088	32

108	556	Galβ1-4GlcNAcβ1-3Galβ1-4GlcNAcβ1-3Galβ1-4GlcNAcβ1-3Galβ1-4GlcNAcβ1-3Galβ1-4GlcNAcβ1-2Manα1-6(Galβ1-4GlcNAcβ1-3Galβ1-4GlcNAcβ1-3Galβ1-4GlcNAcβ1-3Galβ1-4GlcNAcβ1-3Galβ1-4GlcNAcβ1-2Manα1-3)Manβ1-4GlcNAcβ1-4GlcNAcβ-Sp25	1086	889
109	70	Fucα1-2Galβ1-4(Fucα1-3)GlcNAcβ1-3Galβ1-4(Fucα1-3)GlcNAcβ1-3Galβ1-4(Fucα1-3)GlcNAcβ-Sp0	1078	43
110	417	Fucα1-2Galβ1-4GlcNAcβ1-2Manα1-6(Fucα1-2Galβ1-4GlcNAcβ1-2Manα1-3)Manβ1-4GlcNAcβ1-4(Fucα1-6)GlcNAcβ-Sp22	1076	78
111	307	GlcNAcβ1-3Man-Sp10	1075	53
112	580	Galβ1-4GlcNAcβ1-3Galβ1-4GlcNAcβ1-3GalNAcα-Sp14	1074	126
113	582	Galβ1-4GlcNAcβ1-3Galβ1-4GlcNAcβ1-6(Galβ1-4GlcNAcβ1-3Galβ1-4GlcNAcβ1-3)GalNAcα-Sp14	1034	217
114	345	Neu5Acα2-6Galβ1-4GlcNAcβ1-2Manα1-3Manβ1-4GlcNAcβ1-4GlcNAc-Sp12	1008	49
115	192	GlcNAcβ1-6GalNAcα-Sp8	994	27
116	181	GlcNAcβ1-3Galβ-Sp8	985	50
117	552	GlcNAcβ1-3Galβ1-4GlcNAcβ1-6(GlcNAcβ1-3Galβ1-3)GalNAcα-Sp14	977	22
118	483	Neu5Acα2-6Galβ1-4GlcNAcβ1-6(Fucα1-2Galβ1-4(Fucα1-3)GlcNAcβ1-3)Galβ1-4Glc-Sp21	975	43
119	18	GlcN(Gc)β-Sp8	967	128
120	427	Fucα1-3GlcNAcβ1-6(Galβ1-4GlcNAcβ1-3)Galβ1-4Glc-Sp21	957	32
121	222	Fucα1-2(6S)Galβ1-4(6S)Glcβ-Sp0	953	25
122	185	GlcNAcβ1-3Galβ1-4Glcβ-Sp0	936	242
123	309	GlcNAcβ1-4GlcNAcβ-Sp12	922	121
124	310	MurNAcβ1-4GlcNAcβ-Sp10	921	18
125	381	Galβ1-4GlcNAcβ1-6(Fucα1-4(Fucα1-2Galβ1-3)GlcNAcβ1-3)Galβ1-4Glc-Sp21	920	152
126	69	Fucα1-2Galβ1-4(Fucα1-3)GlcNAcβ1-3Galβ1-4(Fucα1-3)GlcNAcβ-Sp0	919	109
127	196	Glcα1-4Glcα-Sp8	912	71
128	72	Fucα1-2Galβ1-4(Fucα1-3)GlcNAcβ-Sp8	904	122
129	278	Neu5Gcα2-3Galβ1-3(Fucα1-4)GlcNAcβ-Sp0	893	23
130	177	GlcNAcβ1-6(GlcNAcβ1-3)GalNAcα-Sp14	883	20
131	526	GlcNAcβ1-2 Manα1-6(GlcNAcβ1-4)(GlcNAcβ1-2Manα1-3)Manβ1-4GlcNAcβ1-4(Fucα1-6)GlcNAc-Sp21	865	179
132	16	GlcNAcβ-Sp0	863	148
133	431	GlcNAcβ1-6(GlcNAcβ1-2)Manα1-6(GlcNAcβ1-4)(GlcNAcβ1-4(GlcNAcβ1-2)Manα1-3)Manβ1-4GlcNAcβ1-4GlcNAc-Sp21	845	51
134	424	Galα1-3(Fucα1-2)Galβ1-4GlcNAcβ1-2Manα1-6(Galα1-3(Fucα1-2)Galβ1-4GlcNAcβ1-2Manα1-3)Manβ1-4GlcNAcβ1-4(Fucα1-6)GlcNAcβ-Sp22	840	29
135	190	GlcNAcβ1-4GlcNAcβ1-4GlcNAcβ1-4GlcNAcβ1-4GlcNAcβ1-Sp8	823	16
136	429	GlcNAcβ1-2Manα1-6(GlcNAcβ1-4)(GlcNAcβ1-4(GlcNAcβ1-2)Manα1-3)Manβ1-4GlcNAcβ1-4GlcNAc-Sp21	819	79
137	289	Galβ1-4(Fucα1-3)(6S)Glcβ-Sp0	818	19
138	301	GlcNAcβ1-6(Galβ1-4GlcNAcβ1-3)Galβ1-4GlcNAc-Sp0	818	34
139	189	GlcNAcβ1-4GlcNAcβ1-4GlcNAcβ1-4GlcNAcβ1-4GlcNAcβ1-4GlcNAcβ1-Sp8	812	118
140	311	Manα1-6Manβ-Sp10	809	170
141	454	GalNAcα1-3(Fucα1-2)Galβ1-3GlcNAcβ1-2Manα1-6(GalNAcα1-3(Fucα1-2)Galβ1-3GlcNAcβ1-2Manα1-3)Manβ1-4GlcNAcβ1-4(Fucα1-6)GlcNAcβ-Sp22	800	37
142	32	(3S)Galβ1-4(Fucα1-3)GlcNAc-Sp0	798	58
143	58	Fucα1-2Galβ1-3GalNAcβ1-3Galα-Sp9	798	79
144	430	GlcNAcβ1-6(GlcNAcβ1-2)Manα1-6(GlcNAcβ1-4)(GlcNAcβ1-2Manα1-3)Manβ1-4GlcNAcβ1-4GlcNAc-Sp21	795	78
145	328	Neu5Acα2-3Galβ1-3(Fucα1-4)GlcNAcβ1-3Galβ1-3(Fucα1-4)GlcNAcβ-Sp0	790	9
146	455	Galβ1-4GlcNAcβ1-6(Galβ1-4GlcNAcβ1-2)Manα1-6(Galβ1-4GlcNAcβ1-2Manα1-3)Manβ1-4GlcNAcβ1-4GlcNAcβ-Sp19	790	106
147	361	Galβ1-4GlcNAcβ1-2Manα1-6(Manα1-3)Manβ1-4GlcNAcβ1-4GlcNAcβ-Sp12	755	13
148	197	Glcα1-6Glcα1-6Glcβ-Sp8	726	75
149	150	Galβ1-3GlcNAcβ-Sp8	716	66
150	557	GlcNAβ1-3Galβ1-3GalNAc-Sp14	710	77
151	362	Fucα1-4(Galβ1-3)GlcNAcβ1-2Manα1-6(Fucα1-4(Galβ1-3)GlcNAcβ1-2Manα1-3)Manβ1-4GlcNAcβ1-4(Fucα1-6)GlcNAcβ-Sp22	708	28
152	344	Neu5Acα2-6Galβ1-4GlcNAcβ1-2Manα1-6Manβ1-4GlcNAcβ1-4GlcNAc-Sp12	705	21
153	333	GalNAcα1-3(Fucα1-2)Galβ1-4GlcNAcβ1-3Galβ1-4GlcNAcβ1-3Galβ1-4GlcNAcβ-Sp0	704	54
154	490	Fucα1-2Galβ1-4GlcNAcβ1-6GalNAcα-Sp14	698	56
155	581	Galβ1-4GlcNAcβ1-3Galβ1-4GlcNAcβ1-6(Galβ1-3)GalNAcα-Sp14	696	38
156	179	GlcNAcβ1-3GalNAcα-Sp8	683	16
157	251	Neu5Acα2-3Galβ1-4(Fucα1-3)GlcNAcβ1-3Galβ1-4(Fucα1-3)GlcNAcβ1-3Galβ1-4(Fucα1-3)GlcNAcβ-Sp0	680	112
158	452	Galα1-3(Fucα1-2)Galβ1-3GlcNAcβ1-2Manα1-6(Galα1-3(Fucα1-2)Galβ1-3GlcNAcβ1-2Manα1-3)Manβ1-4GlcNAcβ1-4(Fucα1-6)GlcNAcβ-Sp22	680	44
159	351	Galβ1-3GlcNAcβ1-2Manα1-6(Galβ1-3GlcNAcβ1-2Manα1-3)Manβ1-4GlcNAcβ1-4(Fucα1-6)GlcNAcβ-Sp22	673	82
160	221	Fucα1-2Galβ1-4(6S)GlcNAcβ-Sp8	668	119
161	442	Fucα1-2Galβ1-4 GlcNAcβ1-2Manα1-6(Fucα1-2Galβ1-4GlcNAcβ1-2(Fucα1-2Galβ1-4GlcNAcβ1-4)Manα1-3)Manβ1-4GlcNAcβ1-4GlcNAcβ-Sp12	655	55

162	562	Galβ1-3GlcNAcβ1-3Galβ1-4GlcNAcβ1-3Galβ1-4GlcNAcβ1-6(Galβ1-3GlcNAcβ1-3Galβ1-4GlcNAcβ1-3Galβ1-4GlcNAβ1-2)Manα1-6(Galβ1-3GlcNAcβ1-3Galβ1-4GlcNAcβ1-3Galβ1-4GlcNAcβ1-2Manα1-3)Manβ1-4GlcNAcβ1-4(Fucα1-6)GlcNAcβ-Sp24	643	61
163	193	GlcNAcβ1-6GalNAcα-Sp14	631	23
164	395	Galβ1-4GlcNAcβ1-2Manα1-6(GlcNAcβ1-2Manα1-3)Manβ1-4GlcNAcβ1-4GlcNAc-Sp12	628	184
165	355	KDNα2-3Galβ1-4Glc-Sp0	626	557
166	306	Neu5Acα2-6Galβ1-4GlcNAcβ1-2Manα1-6(GlcNAcβ1-2Manα1-3)Manβ1-4GlcNAcβ1-4GlcNAcβ-Sp12	622	9
167	94	GalNAcβ1-3GalNAcα-Sp8	621	9
168	350	Galβ1-4GlcNAcβ1-2Manα1-6(Galβ1-4GlcNAcβ1-2Manα1-3)Manβ1-4GlcNAcβ1-4(Fucα1-6)GlcNAcβ-Sp22	617	173
169	372	Fucα1-4(Fucα1-2Galβ1-3)GlcNAcβ1-2Manα1-3(Fucα1-4(Fucα1-2Galβ1-3)GlcNAcβ1-2Manα1-3)Manβ1-4GlcNAcβ1-4GlcNAcβ-Sp19	615	37
170	195	Glcα1-4Glcβ-Sp8	614	95
171	542	Galβ1-3GlcNAcβ1-3Galβ1-4GlcNAcβ1-2Manα1-6(Galβ1-3GlcNAcβ1-3Galβ1-4GlcNAcβ1-2Manα1-3)Manβ1-4GlcNAcβ1-4GlcNAc-Sp25	614	55
172	478	Neu5Acα2-6Galβ1-4GlcNAcβ1-2Manα1-6(Neu5Acα2-6Galβ1-4GlcNAcβ1-2Manα1-3)Manβ1-4GlcNAcβ1-4(Fucα1-6)GlcNAcβ-Sp24	613	149
173	127	Galβ1-3GlcNAcβ1-3Galβ1-4(Fucα1-3)GlcNAcβ-Sp0	612	46
174	184	GlcNAcβ1-3Galβ1-4GlcNAcβ1-3Galβ1-4GlcNAcβ-Sp0	612	25
175	332	GalNAcα1-3(Fucα1-2)Galβ1-4GlcNAcβ1-3Galβ1-4GlcNAcβ-Sp0	600	23
176	186	GlcNAcβ1-4-MDPLys	593	57
177	343	Manα1-6(Neu5Acα2-6Galβ1-4GlcNAcβ1-2Manα1-3)Manβ1-4GlcNAcβ1-4GlcNAc-Sp12	591	4
178	481	Galβ1-4GlcNAcβ1-6(Galβ1-4GlcNAcβ1-2)Manα1-6(Galβ1-4GlcNAcβ1-2Manα1-3)Manβ1-4GlcNAcβ1-4(Fucα1-6)GlcNAcβ-Sp24	588	44
179	161	Galβ1-4GlcNAcβ1-3Galβ1-4(Fucα1-3)GlcNAcβ1-3Galβ1-4(Fucα1-3)GlcNAcβ-Sp0	587	19
180	397	Fucα1-2Galβ1-4GlcNAcβ1-3GalNAcα-Sp14	587	34
181	566	Galβ1-4GlcNAcβ1-3Galβ1-4GlcNAcβ1-2Manα1-6(Galβ1-4GlcNAcβ1-3Galβ1-4GlcNAcβ1-2Manα1-3)Manβ1-4GlcNAcβ1-4(Fucα1-6)GlcNAcβ-Sp24	586	16
182	303	Galβ1-4GlcNAcβ1-6Galβ1-4GlcNAcβ-Sp0	573	46
183	226	GalNAcβ1-4(Neu5Acα2-8Neu5Acα2-8Neu5Acα2-3)Galβ1-4Glcβ-Sp0	570	28
184	54	Galβ1-4GlcNAcβ1-2Manα1-6(Galβ1-4GlcNAcβ1-2Manα1-3)Manβ1-4GlcNAcβ1-4GlcNAcβ-Sp12	569	95
185	322	Neu5Acα2-3Galβ1-4GlcNAcβ1-2Manα1-6(Neu5Acα2-3Galβ1-4GlcNAcβ1-2Manα1-3)Manβ1-4GlcNAcβ1-4GlcNAcβ-Sp12	569	109
186	245	Neu5Acα2-3Galβ1-3GalNAcβ1-3Galα1-4Galβ1-4Glcβ-Sp0	568	11
187	586	GlcNAcβ1-3Galβ1-4GlcNAcβ1-6(GlcNAcβ1-3Galβ1-4GlcNAcβ1-3)GalNAcα-Sp14	556	8
188	14	Manβ-Sp8	555	70
189	238	Neu5Acα2-3Galβ1-3(Fucα1-4)GlcNAcβ-Sp8	553	16
190	406	Galα1-3(Fucα1-2)Galβ1-4(Fucα1-3)Glcβ-Sp21	550	28
191	519	GalNAcα1-3(Fucα1-2)Galβ1-4 GlcNAcβ1-2Manα-Sp0	550	25
192	153	Galβ1-4(Fucα1-3)GlcNAcβ1-3Galβ1-4(Fucα1-3)GlcNAcβ-Sp0	539	36
193	394	GlcNAcβ1-2Manα1-6(Galβ1-4GlcNAcβ1-2Manα1-3)Manβ1-4GlcNAcβ1-4GlcNAc-Sp12	538	228
194	112	Galα1-3GalNAcβ-Sp8	537	53
195	66	Fucα1-2Galβ1-3GlcNAcβ1-3Galβ1-4Glcβ-Sp10	536	40
196	342	Neu5Acα2-6Galβ1-4GlcNAcβ1-2Manα1-6(Manα1-3)Manβ1-4GlcNAcβ1-4GlcNAc-Sp12	535	13
197	563	Galβ1-3GlcNAcβ1-3Galβ1-4GlcNAcβ1-6(Galβ1-3GlcNAcβ1-3Galβ1-4GlcNAβ1-2)Manα1-6(Galβ1-3GlcNAcβ1-3Galβ1-4GlcNAcβ1-2Manα1-3)Manβ1-4GlcNAcβ1-4(Fucα1-6)GlcNAcβ-Sp24	532	36
198	401	Galα1-4Galβ1-4GlcNAcβ1-2Manα1-6(Galα1-4Galβ1-4GlcNAcβ1-2Manα1-3)Manβ1-4GlcNAcβ1-4GlcNAcβ-Sp24	521	141
199	391	Galα1-3Galβ1-3GlcNAcβ1-2Manα1-6(Galα1-3Galβ1-3GlcNAcβ1-2Manα1-3)Manβ1-4GlcNAcβ1-4GlcNAc-Sp19	519	28
200	106	Galα1-3(Fucα1-2)Galβ1-4Glcβ-Sp0	507	76
201	425	Galβ1-3GlcNAcβ1-6(Galβ1-3GlcNAcβ1-2)Manα1-6(Galβ1-3GlcNAcβ1-2Manα1-3)Manβ1-4GlcNAcβ1-4GlcNAcβ-Sp19	497	38
202	261	Neu5Acα2-3Galβ1-4Glcβ-Sp0	488	30
203	180	GlcNAcβ1-3GalNAcα-Sp14	486	62
204	280	Neu5Gcα2-3Galβ1-4(Fucα1-3)GlcNAcβ-Sp0	485	523
205	3	Manα-Sp8	483	87
206	13	Glcβ-Sp8	481	47
207	239	Neu5Acα2-3Galβ1-3(Fucα1-4)GlcNAcβ1-3Galβ1-4(Fucα1-3)GlcNAcβ-Sp0	478	32
208	168	Galβ1-4GlcNAcβ-Sp0	477	40
209	497	Fucα1-2(6S)Galβ1-3(6S)GlcNAcβ-Sp0	475	108
210	24	(3S)Galβ1-4(Fucα1-3)(6S)Glc-Sp0	473	13
211	359	Fucα1-2Galβ1-4(Fucα1-3)GlcNAcβ1-2Manα1-6(Fucα1-2Galβ1-4(Fucα1-3)GlcNAcβ1-2Manα1-3)Manβ1-4GlcNAcβ1-4GlcNAβ-Sp20	469	17
212	369	Galα1-3Galβ1-4(Fucα1-3)GlcNAcβ1-2Manα1-6(Galα1-3Galβ1-4(Fucα1-3)GlcNAcβ1-2Manα1-3)Manβ1-4GlcNAcβ1-4GlcNAcβ-Sp20	469	33
213	20	Galβ1-4GlcNAcβ1-6(Galβ1-4GlcNAcβ1-3)GalNAc-Sp14	466	21
214	284	Neu5Gcα2-6Galβ1-4GlcNAcβ-Sp0	462	38
215	132	Galβ1-4GlcNAcβ1-6GalNAc-Sp14	454	24
216	133	GlcNAcβ1-6(Galβ1-3)GalNAcα-Sp8	451	13

217	103	Galα1-3(Fucα1-2)Galβ1-4(Fucα1-3)GlcNAcβ-Sp0	450	13
218	584	GlcNAcβ1-3Galβ1-4GlcNAcβ1-3GalNAcα-Sp14	450	23
219	585	GlcNAcβ1-3Galβ1-4GlcNAcβ1-6(Galβ1-3)GalNAcα-Sp14	450	49
220	324	Galβ1-4(Fucα1-3)GlcNAcβ1-2Manα1-6(Galβ1-4(Fucα1-3)GlcNAcβ1-2Manα1-3)Manβ1-4GlcNAcβ1-4GlcNAcβ-Sp20	449	55
221	152	Galβ1-4(Fucα1-3)GlcNAcβ-Sp8	446	55
222	304	GalNAcβ1-3Galβ-Sp8	431	27
223	573	Galβ1-4GlcNAcβ1-3Galβ1-4GlcNAcβ1-6(Galβ1-4GlcNAcβ1-3Galβ1-4GlcNAβ1-2)Manα1-6(Galβ1-4GlcNAcβ1-3Galβ1-4GlcNAcβ1-2Manα1-3)Manβ1-4GlcNAcβ1-4(Fucα1-6)GlcNAcβ-Sp24	426	18
224	376	GalNAcβ1-4GlcNAcβ1-2Manα1-6(GalNAcβ1-4GlcNAcβ1-2Manα1-3)Manβ1-4GlcNAcβ1-4GlcNAc-Sp12	423	51
225	57	Neu5Acα2-6Galβ1-4GlcNAcβ1-2Manα1-6(Neu5Acα2-6Galβ1-4GlcNAcβ1-2Manα1-3)Manβ1-4GlcNAcβ1-4GlcNAcβ-Sp24	421	32
226	472	Galβ1-3GlcNAcβ1-2Manα1-6(GlcNAcβ1-4)(Galβ1-3GlcNAcβ1-2Manα1-3)Manβ1-4GlcNAcβ1-4GlcNAcβ-Sp21	416	67
227	85	GalNAcα1-3(Fucα1-2)Galβ1-4GlcNAcβ-Sp0	415	51
228	120	Galα1-4Galβ1-4GlcNAcβ-Sp0	405	15
229	551	Galα1-3Galβ1-4GlcNAcβ1-2Manα1-6(Galα1-3Galβ1-4GlcNAcβ1-2Manα1-3)Manβ1-4GlcNAcβ1-4GlcNAc-Sp24	401	27
230	67	Fucα1-2Galβ1-3GlcNAcβ-Sp0	400	4
231	97	GalNAcβ1-4(Fucα1-3)GlcNAcβ-Sp0	399	61
232	68	Fucα1-2Galβ1-3GlcNAcβ-Sp8	389	25
233	296	(6P)Glcβ-Sp10	385	18
234	199	Glcβ1-6Glcβ-Sp8	381	86
235	356	KDNα2-3Galβ1-3GalNAcα-Sp14	372	324
236	65	Fucα1-2Galβ1-3GlcNAcβ1-3Galβ1-4Glcβ-Sp8	371	89
237	151	Galβ1-4(Fucα1-3)GlcNAcβ-Sp0	370	132
238	392	Galα1-3Galβ1-3(Fucα1-4)GlcNAcβ1-2Manα1-6(Galα1-3Galβ1-3(Fucα1-4)GlcNAcβ1-2Manα1-3)Manβ1-4GlcNAcβ1-4GlcNAc-Sp19	368	112
239	468	Neu5Acα2-3Galβ1-4GlcNAcβ1-6(Neu5Acα2-3Galβ1-4GlcNAcβ1-3)GalNAcα-Sp14	366	181
240	288	Galβ1-4(Fucα1-3)(6S)GlcNAcβ-Sp0	364	25
241	167	Galβ1-4GlcNAcβ1-6(Galβ1-3)GalNAc-Sp14	363	37
242	319	Neu5Acα2-8Neu5Acα2-8Neu5Acβ-Sp8	363	20
243	99	GalNAcβ1-4GlcNAcβ-Sp8	358	40
244	448	GalNAcα1-3(Fucα1-2)Galβ1-4GlcNAcβ1-6(GalNAcα1-3(Fucα1-2)Galβ1-4GlcNAcβ1-3)GalNAc-Sp14	358	117
245	292	Neu5Acα2-3Galβ1-3GlcNAcβ1-3Galβ1-3GlcNAcβ-Sp0	355	23
246	386	Fucα1-2Galβ1-3GalNAcα1-3(Fucα1-2)Galβ1-4Glcβ-Sp0	354	46
247	59	Fucα1-2Galβ1-3GalNAcβ1-3Galα1-4Galβ1-4Glcβ-Sp9	351	12
248	325	Neu5,9Ac2α2-3Galβ1-4GlcNAcβ-Sp0	347	79
249	154	Galβ1-4(Fucα1-3)GlcNAcβ1-3Galβ1-4(Fucα1-3)GlcNAcβ1-3Galβ1-4(Fucα1-3)GlcNAcβ-Sp0	346	28
250	122	Galα1-4Galβ1-4Glcβ-Sp0	345	40
251	160	Galβ1-4GlcNAcβ1-3GalNAc-Sp14	343	154
252	339	GlcNAcα1-4Galβ1-4GlcNAcβ1-3Galβ1-4(Fucα1-3)GlcNAcβ1-3Galβ1-4(Fucα1-3)GlcNAcβ-Sp0	343	35
253	143	Galβ1-3GalNAcβ1-3Galα1-4Galβ1-4Glcβ-Sp0	342	16
254	145	Galβ1-3GalNAcβ1-4Galβ1-4Glcβ-Sp8	342	21
255	198	Glcβ1-4Glcβ-Sp8	342	10
256	317	Galβ1-4GlcNAcβ1-2Manα1-6(Neu5Acα2-6Galβ1-4GlcNAcβ1-2Manα1-3)Manβ1-4GlcNAcβ1-4GlcNAcβ-Sp12	338	41
257	174	GlcNAcα1-6Galβ1-4GlcNAcβ-Sp8	334	28
258	86	GalNAcα1-3(Fucα1-2)Galβ1-4GlcNAcβ-Sp8	333	40
259	260	Fucα1-2Galβ1-4(6S)Glcβ-Sp0	333	22
260	175	GlcNAcβ1-2Galβ1-3GalNAcα-Sp8	331	33
261	34	(3S)Galβ1-4(6S)GlcNAcβ-Sp0	329	43
262	479	Neu5Acα2-3Galβ1-4GlcNAcβ1-2Manα1-6(Neu5Acα2-3Galβ1-4GlcNAcβ1-2Manα1-3)Manβ1-4GlcNAcβ1-4(Fucα1-6)GlcNAcβ-Sp24	329	124
263	212	Manα1-2Manα1-6(Manα1-2Manα1-3)Manα1-6(Manα1-2Manα1-2Manα1-3)Manβ1-4GlcNAcβ1-4GlcNAcβ-Sp12	322	37
264	592	Neu5Acα2-6Galβ1-4GlcNAcβ1-3Galβ1-4GlcNAcβ1-6(Galβ1-3)GalNAcα-Sp14	322	86
265	520	Galβ1-3GlcNAcβ1-2Manα-Sp0	315	77
266	164	Galβ1-4GlcNAcβ1-3Galβ1-4Glcβ-Sp0	312	22
267	299	Neu5Acα2-6Galβ1-4GlcNAcβ1-2Manα1-6(Galβ1-4GlcNAcβ1-2Manα1-3)Manβ1-4GlcNAcβ1-4GlcNAcβ-Sp12	311	16
268	268	Neu5Acα2-6Galβ1-4GlcNAcβ1-3Galβ1-4(Fucα1-3)GlcNAcβ1-3Galβ1-4(Fucα1-3)GlcNAcβ-Sp0	305	29
269	136	Neu5Acα2-6(Galβ1-3)GalNAcα-Sp14	301	63
270	587	Neu5Acα2-3Galβ1-4GlcNAcβ1-3Galβ1-4GlcNAcβ1-6(Neu5Acα2-3Galβ1-4GlcNAcβ1-3Galβ1-4GlcNAcβ1-3)GalNAcα-Sp14	301	34
271	365	Galβ1-4(Fucα1-3)GlcNAcβ1-6(Fucα1-2Galβ1-4GlcNAcβ1-3)Galβ1-4Glc-Sp21	293	18

272	569	GlcNAcβ1-3Galβ1-4GlcNAcβ1-3Galβ1-4GlcNAcβ1-3Galβ1-4GlcNAcβ1-2Manα1-6(GlcNAcβ1-3Galβ1-4GlcNAcβ1-3Galβ1-4GlcNAcβ1-3Galβ1-4GlcNAcβ1-2Manα1-3)Manβ1-4GlcNAcβ1-4(Fucα1-6)GlcNAcβ-Sp24	285	69
273	357	Fucα1-2Galβ1-3GlcNAcβ1-2Manα1-6(Fucα1-2Galβ1-3GlcNAcβ1-2Manα1-3)Manβ1-4GlcNAcβ1-4GlcNAcβ-Sp20	280	15
274	443	Fucα1-2Galβ1-4(Fucα1-3)GlcNAcβ1-2Manα1-6(Fucα1-2Galβ1-4(Fucα1-3)GlcNAcβ1-4(Fucα1-2Galβ1-4(Fucα1-3)GlcNAcβ1-2)Manα1-3)Manβ1-4GlcNAcβ1-4GlcNAcβ-Sp12	279	23
275	105	Galα1-3(Fucα1-2)Galβ1-4GlcNAc-Sp0	277	34
276	337	GlcNAcα1-4Galβ1-3GlcNAcβ-Sp0	275	146
277	353	KDNα2-3Galβ1-4(Fucα1-3)GlcNAc-Sp0	271	44
278	501	Fucα1-2Galβ1-3GlcNAcβ1-6(Fucα1-2Galβ1-3GlcNAcβ1-3)GalNAcα-Sp14	262	45
279	572	Galβ1-4GlcNAcβ1-3Galβ1-4GlcNAcβ1-3Galβ1-4GlcNAcβ1-3Galβ1-4GlcNAcβ1-3Galβ1-4GlcNAcβ1-2Manα1-6(Galβ1-4GlcNAcβ1-3Galβ1-4GlcNAcβ1-3Galβ1-4GlcNAcβ1-3Galβ1-4GlcNAcβ1-3Galβ1-4GlcNAcβ1-2Manα1-3)Manβ1-4GlcNAcβ1-4(Fucα1-6)GlcNAcβ-Sp19	262	92
280	118	Galα1-3Galβ-Sp8	260	11
281	579	Galβ1-4GlcNAcβ1-3Galβ1-4GlcNAcβ1-3Galβ1-4GlcNAcβ1-3Galβ1-4GlcNAcβ1-3Galβ1-4GlcNAcβ1-6(Galβ1-4GlcNAcβ1-3Galβ1-4GlcNAcβ1-3Galβ1-4GlcNAcβ1-3Galβ1-4GlcNAcβ1-3Galβ1-4GlcNAβ1-2)Manα1-6(Galβ1-4GlcNAcβ1-3Galβ1-4GlcNAcβ1-3Galβ1-4GlcNAcβ1-3Galβ1-4GlcNAcβ1-3Galβ1-4GlcNAcβ1-2Manα1-3)Manβ1-4GlcNAcβ1-4(Fucα1-6)GlcNAcβ-Sp24	259	65
282	8	Rhaα-Sp8	257	50
283	230	GalNAcβ1-4(Neu5Acα2-3)Galβ1-4GlcNAcβ-Sp0	256	13
284	2	Glcα-Sp8	255	116
285	204	GlcAβ1-6Galβ-Sp8	255	23
286	428	GlcNAcβ1-2Manα1-6(GlcNAcβ1-4)(GlcNAcβ1-2Manα1-3)Manβ1-4GlcNAcβ1-4GlcNAc-Sp21	246	62
287	527	Galβ1-4GlcNAcβ1-2 Manα1-6(GlcNAcβ1-4)(Galβ1-4GlcNAcβ1-2Manα1-3)Manβ1-4GlcNAcβ1-4(Fucα1-6)GlcNAc-Sp21	246	101
288	282	Neu5Gcα2-3Galβ1-4Glcβ-Sp0	243	12
289	100	Galα1-2Galβ-Sp8	241	28
290	341	GlcNAcα1-4Galβ1-3GalNAc-Sp14	240	86
291	423	Fucα1-2Galβ1-3GlcNAcβ1-2Manα1-6(Fucα1-2Galβ1-3GlcNAcβ1-2Manα1-3)Manβ1-4GlcNAcβ1-4(Fucα1-6)GlcNAcβ-Sp22	240	95
292	336	GlcNAcα1-4Galβ1-4GlcNAcβ-Sp0	239	23
293	596	Neu5Acα2-6Galβ1-4GlcNAcβ1-3Galβ1-4GlcNAcβ1-6(Neu5Acα2-6Galβ1-4GlcNAcβ1-3Galβ1-4GlcNAcβ1-3)GalNAcα-Sp14	239	78
294	55	Neu5Acα2-6Galβ1-4GlcNAcβ1-2Manα1-6(Neu5Acα2-6Galβ1-4GlcNAcβ1-2Manα1-3)Manβ1-4GlcNAcβ1-4GlcNAcβ-Sp12	237	35
295	141	Galβ1-3GalNAcα-Sp16	237	12
296	371	Galα1-3(Fucα1-2)Galβ1-3GlcNAcβ1-2Manα1-6(Galcα1-3(Fucα1-2)Galβ1-3GlcNAcβ1-2Manα1-3)Manβ1-4GlcNAcβ1-4GlcNAcβ-Sp20	235	12
297	266	Neu5Acα2-6Galβ1-4GlcNAcβ-Sp0	233	31
298	243	Neu5Acα2-6(Neu5Acα2-3Galβ1-3)GalNAcα-Sp14	231	14
299	117	Galα1-3Galβ1-4Glc-Sp10	227	24
300	37	(3S)Galβ1-4GlcNAcβ-Sp8	225	79
301	156	Galβ1-4(6S)Glcβ-Sp8	224	13
302	203	GlcAβ1-3Galβ-Sp8	219	16
303	44	(6S)Galβ1-4GlcNAcβ-Sp8	218	49
304	200	G-ol-Sp8	218	75
305	530	Neu5Acα2-3Galβ1-4(Fucα1-3)GlcNAcβ1-2Manα-Sp0	218	74
306	113	Galα1-3Galβ1-4(Fucα1-3)GlcNAcβ-Sp8	217	48
307	593	Neu5Acα2-6Galβ1-4GlcNAcβ1-6(Galβ1-3)GalNAcα-Sp14	213	69
308	116	Galα1-3Galβ1-4Glcβ-Sp0	210	43
309	82	GalNAcα1-3(Fucα1-2)Galβ1-3GlcNAcβ-Sp0	208	6
310	281	Neu5Gcα2-3Galβ1-4GlcNAcβ-Sp0	208	26
311	571	GlcNAcβ1-3Galβ1-4GlcNAcβ1-3Galβ1-4GlcNAcβ1-3Galβ1-4GlcNAcβ1-3Galβ1-4GlcNAcβ1-2Manα1-6(GlcNAcβ1-3Galβ1-4GlcNAcβ1-3Galβ1-4GlcNAcβ1-3Galβ1-4GlcNAcβ1-3Galβ1-4GlcNAcβ1-2Manα1-3)Manβ1-4GlcNAcβ1-4(Fucα1-6)GlcNAcβ-Sp19	208	49
312	271	Neu5Acα2-6Galβ1-4Glcβ-Sp8	206	23
313	33	(3S)Galβ1-4(Fucα1-3)GlcNAc-Sp8	205	19
314	352	(6S)GlcNAcβ1-3Galβ1-4GlcNAcβ-Sp0	205	4
315	4	GalNAcα-Sp8	204	21
316	158	Galβ1-4GalNAcβ1-3(Fucα1-2)Galβ1-4GlcNAcβ-Sp8	204	32
317	255	Neu5Acα2-3Galβ1-4(Fucα1-3)GlcNAcβ1-3Galβ1-4GlcNAcβ-Sp8	204	41
318	591	Neu5Acα2-3Galβ1-4GlcNAcβ1-3Galβ1-4GlcNAcβ1-6(Galβ1-3)GalNAcα-Sp14	204	64
319	205	KDNα2-3Galβ1-3GlcNAcβ-Sp0	203	9
320	5	GalNAcα-Sp15	202	59
321	285	Neu5Gcα-Sp8	200	22
322	464	Galα1-3(Fucα1-2)Galβ1-3GalNAcα-Sp8	199	7
323	426	Galβ1-4GlcNAcβ1-6(Fucα1-2Galβ1-3GlcNAcβ1-3)Galβ1-4Glc-Sp21	198	71
324	300	Galβ1-4GlcNAcβ1-6(Galβ1-4GlcNAcβ1-3)Galβ1-4GlcNAc-Sp0	197	13

325	316	Neu5Acα2-6Galβ1-4GlcNAcβ1-2Manα1-6(Neu5Acα2-3Galβ1-4GlcNAcβ1-2Manα1-3)Manβ1-4GlcNAcβ1-4GlcNAcβ-Sp12	196	14
326	518	Galα1-3(Fucα1-2)Galβ1-4GlcNAcβ1-2Manα-Sp0	196	18
327	109	Galα1-4(Galα1-3)Galβ1-4GlcNAcβ-Sp8	191	29
328	338	GlcNAcα1-4Galβ1-4GlcNAcβ1-3Galβ1-4Glcβ-Sp0	191	23
329	63	Fucα1-2Galβ1-3GalNAcβ1-4(Neu5Acα2-3)Galβ1-4Glcβ-Sp0	190	49
330	513	Galβ1-4(6P)GlcNAcβ-Sp0	190	30
331	131	Galβ1-4GlcNAcβ1-6GalNAcα-Sp8	189	15
332	110	Galα1-3GalNAcα-Sp8	185	18
333	323	Neu5Acα2-3Galβ1-4GlcNAcβ1-2Manα1-6(Neu5Acα2-6Galβ1-4GlcNAcβ1-2Manα1-3)Manβ1-4GlcNAcβ1-4GlcNAcβ-Sp12	185	48
334	396	Neu5Acα2-3Galβ1-3GlcNAcβ1-3GalNAcα-Sp14	185	8
335	262	Neu5Acα2-3Galβ1-4Glcβ-Sp8	183	25
336	291	Galβ1-4GlcNAcβ1-3Galβ1-3GlcNAcβ-Sp0	183	14
337	523	Galα1-3Galβ1-3GlcNAcβ1-2Manα-Sp0	183	53
338	206	KDNα2-3Galβ1-4GlcNAcβ-Sp0	181	18
339	474	Neu5Acα2-3Galβ1-4GlcNAcβ1-2Manα-Sp0	181	163
340	375	Neu5Acα2-3Galβ1-4(Fucα1-3)GlcNAcβ1-3GalNAcα-Sp14	180	61
341	496	Fucα1-2Galβ1-3(6S)GlcNAcβ-Sp0	179	36
342	366	Galβ1-4GlcNAcβ1-2Manα1-6(Galβ1-4GlcNAcβ1-4(Galβ1-4GlcNAcβ1-2)Manα1-3)Manβ1-4GlcNAcβ1-4GlcNAc-Sp21	178	4
343	453	Neu5Acα2-6Galβ1-4GlcNAcβ1-6(Fucα1-2Galβ1-3GlcNAcβ1-3)Galβ1-4Glc-Sp21	177	40
344	570	Galβ1-4GlcNAcβ1-3Galβ1-4GlcNAcβ1-3Galβ1-4GlcNAcβ1-3Galβ1-4GlcNAcβ1-2Manα1-6(Galβ1-4GlcNAcβ1-3Galβ1-4GlcNAcβ1-3Galβ1-4GlcNAcβ1-3Galβ1-4GlcNAcβ1-2Manα1-3)Manβ1-4GlcNAcβ1-4(Fucα1-6)GlcNAcβ-Sp24	177	25
345	56	Neu5Acα2-6Galβ1-4GlcNAcβ1-2Manα1-6(Neu5Acα2-6Galβ1-4GlcNAcβ1-2Man-a1-3)Manβ1-4GlcNAcβ1-4GlcNAcβ-Sp21	176	9
346	155	Galβ1-4(6S)Glcβ-Sp0	174	25
347	272	Neu5Acα2-6Galβ-Sp8	173	9
348	298	Galβ1-3Galβ1-4GlcNAcβ-Sp8	173	16
349	533	Galα1-3(Fucα1-2)Galβ1-3GalNAcβ1-3Galα1-4Galβ1-4Glc-Sp21	172	14
350	87	GalNAcα1-3(Fucα1-2)Galβ1-4Glcβ-Sp0	171	63
351	354	KDNα2-6Galβ1-4GlcNAc-Sp0	171	17
352	413	Fucα1-2Galβ1-4(Fucα1-3)GlcNAcβ1-3GalNAcα-Sp14	169	10
353	12	Galβ-Sp8	168	55
354	43	(6S)Galβ1-4Glcβ-Sp8	167	53
355	524	GalNAcβ1-4GlcNAcβ1-2Manα-Sp0	166	27
356	91	GalNAcα1-3GalNAcβ-Sp8	164	22
357	476	Neu5Acα2-6Galβ1-4GlcNAcβ1-6GalNAcα-Sp14	163	68
358	41	(6P)Manα-Sp8	162	82
359	1	Galα-Sp8	161	64
360	293	Neu5Acα2-3Galβ1-4GlcNAcβ1-3Galβ1-3GlcNAcβ-Sp0	161	10
361	444	Galβ1-4(Fucα1-3)GlcNAcβ1-6GalNAc-Sp14	161	19
362	9	Neu5Acα-Sp8	159	40
363	465	Galα1-3(Fucα1-2)Galβ1-3GalNAcβ-Sp8	159	13
364	98	GalNAcβ1-4GlcNAcβ-Sp0	154	20
365	62	Fucα1-2Galβ1-3GalNAcα-Sp14	152	23
366	219	(3S)Galβ1-4(Fucα1-3)(6S)GlcNAcβ-Sp8	151	34
367	257	Neu5Acα2-3Galβ1-4GlcNAcβ-Sp0	151	9
368	330	Galα1-4Galβ1-4GlcNAcβ1-3Galβ1-4Glcβ-Sp0	150	23
369	101	Galα1-3(Fucα1-2)Galβ1-3GlcNAcβ-Sp0	149	34
370	528	Galβ1-4GlcNAcβ1-2 Manα1-6(Galβ1-4GlcNAcβ1-4)(Galβ1-4GlcNAcβ1-2Manα1-3)Manβ1-4GlcNAcβ1-4(Fucα1-6)GlcNAc-Sp21	148	24
371	370	GalNAcα1-3(Fucα1-2)Galβ1-3GlcNAcβ1-2Manα1-6(GalNAcα1-3(Fucα1-2)Galβ1-3GlcNAcβ1-2Manα1-3)Manβ1-4GlcNAcβ1-4GlcNAcβ-Sp20	147	32
372	46	Neu5Acα2-3(6S)Galβ1-4GlcNAcβ-Sp8	146	13
373	287	Galβ1-3GlcNAcβ1-3Galβ1-3GlcNAcβ-Sp0	145	11
374	515	GalNAcα1-3(Fucα1-2)Galβ1-4GlcNAcβ1-6GalNAc-Sp14	145	18
375	270	Neu5Acα2-6Galβ1-4Glcβ-Sp0	144	35
376	166	Galβ1-4GlcNAcβ1-6(Galβ1-3)GalNAcα-Sp8	143	9
377	529	Fucα1-4(Galβ1-3)GlcNAcβ1-2 Manα-Sp0	143	59
378	140	Galβ1-3GalNAcα-Sp14	138	42
379	577	Galβ1-4GlcNAcβ1-3Galβ1-4GlcNAcβ1-3Galβ1-4GlcNAcβ1-3Galβ1-4GlcNAcβ1-6(Galβ1-4GlcNAcβ1-3Galβ1-4GlcNAcβ1-3Galβ1-4GlcNAcβ1-3Galβ1-4GlcNAβ1-2)Manα1-6(Galβ1-4GlcNAcβ1-3Galβ1-4GlcNAcβ1-3Galβ1-4GlcNAcβ1-3Galβ1-4GlcNAcβ1-2Manα1-3)Manβ1-4GlcNAcβ1-4(Fucα1-6)GlcNAcβ-Sp24	138	19
380	256	Neu5Acα2-3Galβ1-4GlcNAcβ1-3Galβ1-4GlcNAcβ1-3Galβ1-4GlcNAcβ-Sp0	136	23
381	15	GalNAcβ-Sp8	135	40
382	334	Neu5Acα2-3-Galβ1-4(Fucα1-3)GlcNAcβ1-6(Neu5Acα2-3Galβ1-3)GalNAc-Sp14	135	5

383	384	Galβ1-4GlcNAcβ1-6(Galβ1-4GlcNAcβ1-2)Manα1-6(Galβ1-4GlcNAcβ1-4(Galβ1-4GlcNAcβ1-2)Manα1-3)Manβ1-4GlcNAcβ1-4GlcNAcβ-Sp21	135	20
384	595	GlcNAcβ1-6(Neu5Acα2-3Galβ1-3)GalNAcα-Sp14	135	16
385	250	Neu5Acα2-3Galβ1-4(Fucα1-3)(6S)GlcNAcβ-Sp8	134	15
386	273	Neu5Acα2-8Neu5Acα-Sp8	134	11
387	578	GlcNAcβ1-3Galβ1-4GlcNAcβ1-3Galβ1-4GlcNAcβ1-3Galβ1-4GlcNAcβ1-3Galβ1-4GlcNAcβ1-6(GlcNAcβ1-3Galβ1-4GlcNAcβ1-3Galβ1-4GlcNAcβ1-3Galβ1-4GlcNAcβ1-3Galβ1-4GlcNAβ1-2)Manα1-6(GlcNAcβ1-3Galβ1-4GlcNAcβ1-3Galβ1-4GlcNAcβ1-3Galβ1-4GlcNAcβ1-3Galβ1-4GlcNAcβ1-2Manα1-3)Manβ1-4GlcNAcβ1-4(Fucα1-6)GlcNAcβ-Sp24	134	46
388	114	Galα1-3Galβ1-3GlcNAcβ-Sp0	132	43
389	42	(6S)Galβ1-4Glcβ-Sp0	131	42
390	19	Galβ1-4GlcNAcβ1-6(Galβ1-4GlcNAcβ1-3)GalNAcα-Sp8	128	19
391	387	Fucα1-2Galβ1-3GalNAcα1-3(Fucα1-2)Galβ1-4GlcNAcβ-Sp0	128	9
392	125	Galβ1-2Galβ-Sp8	127	36
393	482	Neu5Acα2-3Galβ1-3GlcNAcβ1-2Manα1-6(GlcNAcβ1-4)(Neu5Acα2-3Galβ1-3GlcNAcβ1-2Manα1-3)Manβ1-4GlcNAcβ1-4GlcNAc-Sp21	127	19
394	589	GlcNAcβ1-3Galβ1-4GlcNAcβ1-3Galβ1-4GlcNAcβ1-3GalNAcα-Sp14	127	15
395	225	GalNAcβ1-4(Neu5Acα2-8Neu5Acα2-8Neu5Acα2-8Neu5Acα2-3)Galβ1-4Glcβ-Sp0	126	28
396	419	Fucα1-2Galβ1-3GlcNAcβ1-3GalNAc-Sp14	125	8
397	512	(4S)GalNAcβ-Sp10	125	21
398	144	Galβ1-3GalNAcβ1-4(Neu5Acα2-3)Galβ1-4Glcβ-Sp0	124	6
399	163	Galβ1-4GlcNAcβ1-3Galβ1-4GlcNAcβ-Sp0	124	4
400	601	Galβ1-3GalNAcβ1-4(Neu5Acα2-8Neu5Acα2-8Neu5Acα2-3)Galβ1-4Glcβ-Sp21	124	20
401	233	Neu5Acα2-3Galβ1-3GalNAcβ1-4(Neu5Acα2-3)Galβ1-4Glcβ-Sp0	123	9
402	320	Neu5Gcβ2-6Galβ1-4GlcNAc-Sp8	122	8
403	492	Galβ1-4(Fucα1-3)GlcNAcβ1-2Manα-Sp0	120	23
404	115	Galα1-3Galβ1-4GlcNAcβ-Sp8	119	28
405	124	Galα1-6Glcβ-Sp8	119	10
406	149	Galβ1-3GlcNAcβ-Sp0	118	28
407	541	Galβ1-4GlcNAcβ1-3Galβ1-4GlcNAcβ1-3Galβ1-4GlcNAcβ1-2Manα1-6(Galβ1-4GlcNAcβ1-3Galβ1-4GlcNAcβ1-3Galβ1-4GlcNAcβ1-2Manα1-3)Manβ1-4GlcNAcβ1-4GlcNAcβ-Sp24	118	17
408	283	Neu5Gcα2-6GalNAcα-Sp0	116	3
409	36	(3S)Galβ1-4GlcNAcβ-Sp0	115	15
410	380	Galβ1-4(Fucα1-3)GlcNAcβ1-6(Galβ1-3GlcNAcβ1-3)Galβ1-4Glc-Sp21	115	27
411	445	Galβ1-4GlcNAcβ1-2Manα-Sp0	115	35
412	26	(3S)Galβ1-4(6S)Glcβ-Sp0	114	27
413	477	Neu5Acα2-6Galβ1-4 GlcNAcβ1-6(Neu5Acα2-6Galβ1-4GlcNAcβ1-3)GalNAcα-Sp14	114	21
414	172	Galβ1-4Glcβ-Sp8	113	14
415	421	GalNAcα1-3(Fucα1-2)Galβ1-3GlcNAcβ1-3GalNAc-Sp14	113	18
416	489	Galβ1-4(Fucα1-3)GlcNAcβ1-6(Neu5Acα2-6(Neu5Acα2-3Galβ1-3)GlcNAcβ1-3)Galβ1-4Glc-Sp21	113	10
417	64	Fucα1-2Galβ1-3GalNAcβ1-4(Neu5Acα2-3)Galβ1-4Glcβ-Sp9	112	23
418	121	Galα1-4Galβ1-4GlcNAcβ-Sp8	112	5
419	294	4S(3S)Galβ1-4GlcNAcβ-Sp0	112	13
420	331	GalNAcβ1-3Galα1-4Galβ1-4GlcNAcβ1-3Galβ1-4Glcβ-Sp0	112	18
421	45	(6S)Galβ1-4(6S)Glcβ-Sp8	110	7
422	162	Galβ1-4GlcNAcβ1-3Galβ1-4GlcNAcβ1-3Galβ1-4GlcNAcβ-Sp0	110	23
423	326	Neu5,9Ac2α2-3Galβ1-3GlcNAcβ-Sp0	108	10
424	415	GalNAcα1-3(Fucα1-2)Galβ1-4(Fucα1-3)GlcNAcβ1-3GalNAc-Sp14	108	26
425	521	Galα1-3(Fucα1-2)Galβ1-3GlcNAcβ1-6GalNAc-Sp14	108	18
426	335	GlcNAcα1-4Galβ1-4GlcNAcβ1-3Galβ1-4GlcNAcβ1-3Galβ1-4GlcNAcβ-Sp0	106	28
427	223	Neu5Acα2-3Galβ1-3GalNAcα-Sp8	105	26
428	305	GlcAβ1-3GlcNAcβ-Sp8	105	111
429	379	Galβ1-3GlcNAcβ1-3Galβ1-4GlcNAcβ1-6(Galβ1-3GlcNAcβ1-3)Galβ1-4Glcβ-Sp21	105	8
430	450	GalNAcβ1-4Galβ1-4Glcβ-Sp0	105	11
431	504	Galβ1-4GlcNAcβ1-6(Galβ1-4GlcNAcβ1-2)Manα1-6(GlcNAcβ1-4)Galβ1-4GlcNAcβ1-4(Gal β1-4GlcNAcβ1-2)Manα1-3)Manβ1-4GlcNAcβ1-4(Fucα1-6)GlcNAc-Sp21	105	11
432	403	Galβ1-3GlcNAcβ1-6Galβ1-4GlcNAcβ-Sp0	104	16
433	27	(3S)Galβ1-4(6S)Glcβ-Sp8	103	30
434	49	Neu5,9Ac2α2-6Galβ1-4GlcNAcβ-Sp8	103	17
435	123	Galα1-4GlcNAcβ-Sp8	103	7
436	495	Fucα1-2Galβ1-4GlcNAcβ1-2Manα-Sp0	103	1
437	254	Neu5Acα2-3Galβ1-4(Fucα1-3)GlcNAcβ1-3Galβ-Sp8	102	11
438	411	GalNAcα1-3(Fucα1-2)Galβ1-4GlcNAcβ1-3GalNAcα-Sp14	102	10
439	561	(3S)GlcAβ1-3Galβ1-4GlcNAcβ1-2Manα-Sp0	102	16
440	558	Galβ1-3GlcNAcβ1-6(Galβ1-3)GalNAc-Sp14	101	31
441	25	(3S)Galβ1-4Glcβ-Sp8	100	9
442	170	Galβ1-4GlcNAcβ-Sp23	100	7

443	433	Galβ1-4GlcNAcβ1-2Manα1-6(GlcNAcβ1-4)(Galβ1-4GlcNAcβ1-4(Galβ1-4GlcNAcβ1-2)Manα1-3)Manβ1-4GlcNAcβ1-4GlcNAc-Sp21	100	19
444	522	Neu5Acα2-3Galβ1-3GlcNAcβ1-2Manα-Sp0	100	13
445	279	Neu5Gcα2-3Galβ1-3GlcNAcβ-Sp0	99	14
446	315	Neu5Acα2-3Galβ1-4GlcNAcβ1-6(Neu5Acα2-3Galβ1-3)GalNAcα-Sp14	99	29
447	473	Neu5Acα2-6Galβ1-4GlcNAcβ1-6(Galβ1-3GlcNAcβ1-3)Galβ1-4Glcβ-Sp21	99	19
448	494	Galα1-3(Fucα1-2)Galβ1-4GlcNAcβ1-6GalNAcα-Sp14	99	15
449	30	(3S)Galβ1-3GlcNAcβ-Sp0	98	8
450	302	Galβ1-4GlcNAcα1-6Galβ1-4GlcNAcβ-Sp0	98	19
451	383	Galβ1-3GlcNAcβ1-3Galβ1-4(Fucα1-3)GlcNAcβ1-6(Galβ1-3GlcNAcβ1-3)Galβ1-4Glc-Sp21	98	8
452	405	GalNAcβ1-3Galα1-6Galβ1-4Glcβ-Sp8	98	2
453	35	(3S)Galβ1-4(6S)GlcNAcβ-Sp8	97	11
454	38	(3S)Galβ-Sp8	97	85
455	96	GalNAcβ1-3Galα1-4Galβ1-4GlcNAcβ-Sp0	96	30
456	171	Galβ1-4Glcβ-Sp0	96	6
457	235	Neu5Acα2-3GalNAcα-Sp8	96	52
458	253	Neu5Acα2-3Galβ1-4(Fucα1-3)GlcNAcβ-Sp8	96	6
459	364	Neu5Acα2-6GlcNAcβ1-4GlcNAcβ1-4GlcNAc-Sp21	96	5
460	432	Galβ1-4GlcNAcβ1-2Manα1-6(GlcNAcβ1-4)(Galβ1-4GlcNAcβ1-2Manα1-3)Manβ1-4GlcNAcβ1-4GlcNAc-Sp21	96	14
461	437	Galβ1-6Galβ-Sp10	96	19
462	554	GalNAcβ1-4GlcNAcβ1-3GalNAcβ1-4GlcNAcβ-Sp0	96	8
463	40	(4S)Galβ1-4GlcNAcβ-Sp8	95	14
464	590	Galβ1-4GlcNAcβ1-3Galβ1-3GalNAcα-Sp14	95	8
465	157	Galβ1-4GalNAcα1-3(Fucα1-2)Galβ1-4GlcNAcβ-Sp8	94	9
466	227	Neu5Acα2-8Neu5Acα2-8Neu5Acα2-3Galβ1-4Glcβ-Sp0	94	6
467	249	Neu5Acα2-3Galβ1-4(6S)GlcNAcβ-Sp8	94	11
468	286	Neu5Acα2-3Galβ1-4GlcNAcβ1-6(Galβ1-3)GalNAcα-Sp14	94	41
469	398	Galβ1-4(Fucα1-3)GlcNAcβ1-3GalNAcα-Sp14	94	10
470	420	Galα1-3(Fucα1-2)Galβ1-3GlcNAcβ1-3GalNAc-Sp14	94	17
471	435	Galβ1-4GlcNAcβ1-6(Galβ1-4GlcNAcβ1-2)Manα1-6(GlcNAcβ1-4)(Galβ1-4GlcNAcβ1-4(Galβ1-4GlcNAcβ1-2)Manα1-3)Manβ1-4GlcNAcβ1-4GlcNAc-Sp21	94	11
472	447	Galα1-3(Fucα1-2)Galβ1-4GlcNAcβ1-6(Galα1-3(Fucα1-2)Galβ1-4GlcNAcβ1-3)GalNAc-Sp14	94	19
473	493	Fucα1-2(6S)Galβ1-3GlcNAcβ-Sp0	94	5
474	532	GalNAcα1-3(Fucα1-2)Galβ1-3GalNAcβ1-3Galα1-4Galβ1-4Glc-Sp21	94	35
475	147	Galβ1-3GlcNAcβ1-3Galβ1-4GlcNAcβ-Sp0	93	5
476	409	Neu5Acα2-3Galβ1-3GalNAcβ1-4(Neu5Acα2-8Neu5Acα2-3)Galβ1-4Glcβ-Sp0	93	6
477	564	Neu5Acα2-8Neu5Acα2-3Galβ1-3GalNAcβ1-4(Neu5Acα2-3)Galβ1-4Glc-Sp21	93	6
478	252	Neu5Acα2-3Galβ1-4(Fucα1-3)GlcNAcβ-Sp0	92	14
479	22	6S(3S)Galβ1-4(6S)GlcNAcβ-Sp0	91	8
480	402	Galα1-3Galβ1-4GlcNAcβ1-3GalNAcα-Sp14	91	13
481	410	Galα1-3(Fucα1-2)Galβ1-4GlcNAcβ1-3GalNAcα-Sp14	91	11
482	517	Galα1-3Galβ1-4GlcNAcβ1-2Manα-Sp0	91	10
483	244	Neu5Acα2-3Galβ-Sp8	90	6
484	29	(3S)Galβ1-3GalNAcα-Sp8	89	6
485	39	(6S)(4S)Galβ1-4GlcNAcβ-Sp0	89	9
486	159	Galβ1-4GlcNAcβ1-3GalNAcα-Sp8	88	5
487	258	Neu5Acα2-3Galβ1-4GlcNAcβ-Sp8	88	6
488	434	Galβ1-4GlcNAcβ1-6(Galβ1-4GlcNAcβ1-2)Manα1-6(GlcNAcβ1-4)(Galβ1-4GlcNAcβ1-2Manα1-3)Manβ1-4GlcNAcβ1-4GlcNAc-Sp21	88	5
489	499	GalNAcβ1-4(Fucα1-3)(6S)GlcNAcβ-Sp8	88	8
490	134	GlcNAcβ1-6(Galβ1-3)GalNAcα-Sp14	87	28
491	241	Neu5Acα2-3Galβ1-3(6S)GalNAcα-Sp8	87	6
492	388	Galβ1-3GlcNAcβ1-3GalNAcα-Sp14	87	18
493	500	(3S)GalNAcβ1-4(Fucα1-3)GlcNAcβ-Sp8	87	14
494	516	Neu5Acα2-6Galβ1-4GlcNAcβ1-2Man-Sp0	87	7
495	264	Neu5Acα2-6GalNAcβ1-4GlcNAcβ-Sp0	86	7
496	407	Galβ1-4GlcNAcβ1-6(Neu5Acα2-6Galβ1-3GlcNAcβ1-3)Galβ1-4Glc-Sp21	86	7
497	460	Neu5Acα2-6Galβ1-4GlcNAcβ1-2Manα1-6(GlcNAcβ1-4)(Neu5Acα2-6Galβ1-4GlcNAcβ1-2Manα1-3)Manβ1-4GlcNAcβ1-4GlcNAcβ-Sp21	86	7
498	210	Manα1-6(Manα1-2Manα1-3)Manα1-6(Manα1-2Manα1-3)Manβ1-4GlcNAcβ1-4GlcNAcβ-Sp12	85	10
499	374	Neu5Acα2-6Galβ1-4GlcNAcβ1-3GalNAc-Sp14	85	14
500	399	GalNAcα1-3GalNAcβ1-3Galα1-4Galβ1-4GlcNAcβ-Sp0	85	11
501	408	Galβ1-3GalNAcβ1-4(Neu5Acα2-8Neu5Acα2-3)Galβ1-4Glcβ-Sp0	85	6
502	575	Galβ1-4GlcNAcβ1-3Galβ1-4GlcNAcβ1-3Galβ1-4GlcNAcβ1-6(Galβ1-4GlcNAcβ1-3Galβ1-4GlcNAcβ1-3Galβ1-4GlcNAβ1-2)Manα1-6(Galβ1-4GlcNAcβ1-3Galβ1-4GlcNAcβ1-3Galβ1-4GlcNAcβ1-2Manα1-3)Manβ1-4GlcNAcβ1-4(Fucα1-6)GlcNAcβ-Sp24	85	5
503	475	Neu5Acα2-3Galβ1-4GlcNAcβ1-6GalNAcα-Sp14	84	14

504	31	(3S)Galβ1-3GlcNAcβ-Sp8	83	6
505	146	Galβ1-3Galβ-Sp8	83	15
506	169	Galβ1-4GlcNAcβ-Sp8	83	8
507	231	GalNAcβ1-4(Neu5Acα2-3)Galβ1-4GlcNAcβ-Sp8	83	4
508	267	Neu5Acα2-6Galβ1-4GlcNAcβ-Sp8	83	9
509	553	GalNAcβ1-3GlcNAcβ-Sp0	83	9
510	588	Neu5Acα2-6Galβ1-4GlcNAcβ1-3Galβ1-4GlcNAcβ1-3GalNAcα-Sp14	83	6
511	10	Neu5Acα-Sp11	82	7
512	139	Galβ1-3GalNAcα-Sp8	82	9
513	228	GalNAcβ1-4(Neu5Acα2-8Neu5Acα2-3)Galβ1-4Glcβ-Sp0	82	3
514	242	Neu5Acα2-6(Neu5Acα2-3Galβ1-3)GalNAcα-Sp8	82	5
515	318	Neu5Acα2-8Neu5Acβ-Sp17	82	5
516	378	Galβ1-3GalNAcα1-3(Fucα1-2)Galβ1-4GlcNAc-Sp0	82	7
517	404	Galβ1-3GlcNAcα1-6Galβ1-4GlcNAcβ-Sp0	82	5
518	491	Galα1-3Galβ1-4GlcNAcβ1-6GalNAcα-Sp14	82	8
519	525	Neu5Acα2-3Galβ1-3GlcNAcβ1-4Galβ1-4Glcβ-Sp0	82	9
520	539	GlcNAcβ1-3Galβ1-4GlcNAcβ1-3Galβ1-4GlcNAcβ1-2Manα1-6(GlcNAcβ1-3Galβ1-4GlcNAcβ1-3Galβ1-4GlcNAcβ1-2Manα1-3)Manβ1-4GlcNAcβ1-4GlcNAcβ-Sp25	82	10
521	276	Neu5Acβ2-6GalNAcα-Sp8	81	9
522	436	Galβ1-4Galβ-Sp10	81	9
523	23	6S(3S)Galβ1-4GlcNAcβ-Sp0	80	9
524	92	GalNAcα1-3Galβ-Sp8	80	7
525	229	Neu5Acα2-8Neu5Acα2-8Neu5Acα-Sp8	80	5
526	265	Neu5Acα2-6Galβ1-4(6S)GlcNAcβ-Sp8	80	3
527	422	Galα1-3Galβ1-3GlcNAcβ1-3GalNAc-Sp14	80	9
528	576	GlcNAcβ1-3Galβ1-4GlcNAcβ1-3Galβ1-4GlcNAcβ1-3Galβ1-4GlcNAcβ1-6(GlcNAcβ1-3Galβ1-4GlcNAcβ1-3Galβ1-4GlcNAcβ1-3Galβ1-4GlcNAβ1-2)Manα1-6(GlcNAcβ1-3Galβ1-4GlcNAcβ1-3Galβ1-4GlcNAcβ1-3Galβ1-4GlcNAcβ1-2Manα1-3)Manβ1-4GlcNAcβ1-4(Fucα1-6)GlcNAcβ-Sp24	80	6
529	373	Neu5Acα2-3Galβ1-4GlcNAcβ1-3GalNAc-Sp14	79	2
530	439	GalNAcβ1-6GalNAcβ-Sp8	79	9
531	414	Galα1-3(Fucα1-2)Galβ1-4(Fucα1-3)GlcNAcβ1-3GalNAc-Sp14	78	6
532	456	Neu5Acα2-3Galβ1-4GlcNAcβ1-2Manα1-6(GlcNAcβ1-4)(Neu5Acα2-3Galβ1-4GlcNAcβ1-2Manα1-3)Manβ1-4GlcNAcβ1-4GlcNAcβ-Sp21	78	4
533	246	Neu5Acα2-3Galβ1-3GlcNAcβ1-3Galβ1-4GlcNAcβ-Sp0	77	4
534	269	Neu5Acα2-6Galβ1-4GlcNAcβ1-3Galβ1-4GlcNAcβ-Sp0	77	5
535	390	GalNAcα1-3(Fucα1-2)Galβ1-3GalNAcα1-3(Fucα1-2)Galβ1-4GlcNAcβ-Sp0	76	5
536	438	Neu5Acα2-3Galβ1-4GlcNAcβ1-3Galβ-Sp8	76	9
537	568	Galβ1-4GlcNAcβ1-3Galβ1-4GlcNAcβ1-3Galβ1-4GlcNAcβ1-2Manα1-6(Galβ1-4GlcNAcβ1-3Galβ1-4GlcNAcβ1-3Galβ1-4GlcNAcβ1-2Manα1-3)Manβ1-4GlcNAcβ1-4(Fucα1-6)GlcNAcβ-Sp24	75	11
538	232	GalNAcβ1-4(Neu5Acα2-3)Galβ1-4Glcβ-Sp0	74	6
539	412	GalNAcα1-3GalNAcβ1-3Galα1-4Galβ1-4Glcβ-Sp0	74	8
540	583	Neu5Acα2-3Galβ1-4GlcNAcβ1-3Galβ1-4GlcNAcβ1-3GalNAcα-Sp14	74	2
541	240	Neu5Acα2-3Galβ1-4(Neu5Acα2-3Galβ1-3)GlcNAcβ-Sp8	73	8
542	263	Neu5Acα2-6GalNAcα-Sp8	73	6
543	461	Neu5Acα2-6Galβ1-4GlcNAcβ1-4Manα1-6(GlcNAcβ1-4)(Neu5Acα2-6Galβ1-4GlcNAcβ1-4(Neu5Acα2-6Galβ1-4GlcNAcβ1-2)Manα1-3)Manβ1-4GlcNAcβ1-4GlcNAcβ-Sp21	73	13
544	138	Neu5Acα2-6(Galβ1-3)GlcNAcβ1-4Galβ1-4Glcβ-Sp10	72	11
545	457	Neu5Acα2-3Galβ1-4GlcNAcβ1-4Manα1-6(GlcNAcβ1-4)(Neu5Acα2-3Galβ1-4GlcNAcβ1-4(Neu5Acα2-3Galβ1-4GlcNAcβ1-2)Manα1-3)Manβ1-4GlcNAcβ1-4GlcNAcβ-Sp21	72	3
546	135	Neu5Acα2-6(Galβ1-3)GalNAcα-Sp8	71	7
547	218	Neu5Acα2-3Galβ1-4GlcNAcβ1-3Galβ1-4(Fucα1-3)GlcNAcβ-Sp0	71	19
548	277	Neu5Acβ2-6Galβ1-4GlcNAcβ-Sp8	71	2
549	295	(6S)Galβ1-4(6S)GlcNAcβ-Sp0	71	5
550	462	Neu5Acα2-6Galβ1-4GlcNAcβ1-6(Neu5Acα2-6Galβ1-4GlcNAcβ1-2)Manα1-6(GlcNAcβ1-4)(Neu5Acα2-6Galβ1-4GlcNAcβ1-2Manα1-3)Manβ1-4GlcNAcβ1-4GlcNAcβ-Sp21	71	1
551	463	Neu5Acα2-6Galβ1-4GlcNAcβ1-6(Neu5Acα2-6Galβ1-4GlcNAcβ1-2)Manα1-6(GlcNAcβ1-4)(Neu5Acα2-6Galβ1-4GlcNAcβ1-4(Neu5Acα2-6Galβ1-4GlcNAcβ1-2)Manα1-3)Manβ1-4GlcNAcβ1-4GlcNAcβ-Sp21	71	11
552	485	Galα1-3Galβ1-3GlcNAcβ1-6GalNAcα-Sp14	71	15
553	543	Neu5Gcα2-8Neu5Gcα2-3Galβ1-4GlcNAc-Sp0	71	6
554	484	Galβ1-3GlcNAcβ1-6GalNAcα-Sp14	70	5
555	148	Galβ1-3GlcNAcβ1-3Galβ1-4Glcβ-Sp10	69	6
556	6	Fucα-Sp8	67	7
557	234	Neu5Acα2-6(Neu5Acα2-3)GalNAcα-Sp8	67	20
558	498	Neu5Acα2-6GalNAcβ1-4(6S)GlcNAcβ-Sp8	67	7
559	506	Galβ1-3(6S)GlcNAcβ-Sp8	67	7
560	48	Neu5,9Ac2α-Sp8	66	9
561	329	Neu5Acα2-6Galβ1-4GlcNAcβ1-3Galβ1-4GlcNAcβ1-3Galβ1-4GlcNAcβ-Sp0	66	3
562	416	Galβ1-4(Fucα1-3)GlcNAcβ1-2Manα1-6(Galβ1-4(Fucα1-3)GlcNAcβ1-2Manα1-3)Manβ1-4GlcNAcβ1-4(Fucα1-6)GlcNAcβ-Sp22	66	23

563	459	Neu5Acα2-3Galβ1-4GlcNAcβ1-6(Neu5Acα2-3Galβ1-4GlcNAcβ1-2)Manα1-6(GlcNAcβ1-4)(Neu5Acα2-3Galβ1-4GlcNAcβ1-4(Neu5Acα2-3Galβ1-4GlcNAcβ1-2)Manα1-3)Manβ1-4GlcNAcβ1-4GlcNAcβ-Sp21	65	2
564	236	Neu5Acα2-3GalNAcβ1-4GlcNAcβ-Sp0	64	10
565	505	Galβ1-3GlcNAcα1-3Galβ1-4GlcNAcβ-Sp8	64	8
566	137	Neu5Acβ2-6(Galβ1-3)GalNAcα-Sp8	62	4
567	507	(6S)(4S)GalNAcβ1-4GlcNAc-Sp8	62	6
568	393	Neu5Acα2-3Galβ1-3GlcNAcβ1-2Manα1-6(Neu5Acα2-3Galβ1-3GlcNAcβ1-2Manα1-3)Manβ1-4GlcNAcβ1-4GlcNAc-Sp19	60	10
569	458	Neu5Acα2-3Galβ1-4GlcNAcβ1-6(Neu5Acα2-3Galβ1-4GlcNAcβ1-2)Manα1-6(GlcNAcβ1-4)(Neu5Acα2-3Galβ1-4GlcNAcβ1-2Manα1-3)Manβ1-4GlcNAcβ1-4GlcNAcβ-Sp21	60	5
570	11	Neu5Acβ-Sp8	59	11
571	502	GalNAcα1-3(Fucα1-2)Galβ1-3GlcNAcβ1-6GalNAcα-Sp14	59	33
572	508	(6S)GalNAcβ1-4GlcNAc-Sp8	59	8
573	544	Neu5Acα2-8Neu5Gcα2-3Galβ1-4GlcNAc-Sp0	59	11
574	545	Neu5Gcα2-8Neu5Acα2-3Galβ1-4GlcNAc-Sp0	59	7
575	546	Neu5Gcα2-8Neu5Gcα2-3Galβ1-4GlcNAcβ1-3Galβ1-4GlcNAc-Sp0	58	4
576	547	Neu5Gcα2-8Neu5Gcα2-6Galβ1-4GlcNAc-Sp0	58	7
577	511	(3S)GalNAcβ1-4GlcNAc-Sp8	57	9
578	514	(6P)Galβ1-4GlcNAcβ-SP0	56	5
579	548	Neu5Acα2-8Neu5Acα2-3Galβ1-4GlcNAc-Sp0	54	6
580	559	Galβ1-4GlcNAcβ1-3Galβ1-4GlcNAcβ1-3Galβ1-4GlcNAcβ1-3Galβ1-4GlcNAcβ1-3Galβ1-4GlcNAcβ1-3Galβ1-4GlcNAcβ1-2Manα1-6(Galβ1-4GlcNAcβ1-3Galβ1-4GlcNAcβ1-3Galβ1-4GlcNAcβ1-3Galβ1-4GlcNAcβ1-3Galβ1-4GlcNAcβ1-3Galβ1-4GlcNAcβ1-2Manα1-3)Manβ1-4GlcNAcβ1-4GlcNAcβ-Sp25	54	18
581	509	(3S)GalNAcβ1-4(3S)GlcNAc-Sp8	52	4
582	560	(3S)GlcAβ1-3Galβ1-4GlcNAcβ1-3Galβ1-4Glc-Sp0	52	5
583	440	(6S)Galβ1-3GlcNAcβ-Sp0	46	13
584	550	Galβ1-4GlcNAcβ1-3Galβ1-4GlcNAcβ1-6(Galβ1-4GlcNAcβ1-3Galβ1-4GlcNAcβ1-2)Manα1-6(Galβ1-4GlcNAcβ1-3Galβ1-4GlcNAcβ1-2Manα1-3)Manβ1-4GlcNAcβ1-4GlcNAc-Sp24	42	85
585	377	Galβ1-3GalNAcα1-3(Fucα1-2)Galβ1-4Glc-Sp0	41	5
586	574	GlcNAcβ1-3Galβ1-4GlcNAcβ1-3Galβ1-4GlcNAcβ1-6(GlcNAcβ1-3Galβ1-4GlcNAcβ1-3Galβ1-4GlcNAβ1-2)Manα1-6(GlcNAcβ1-3Galβ1-4GlcNAcβ1-3Galβ1-4GlcNAcβ1-2Manα1-3)Manβ1-4GlcNAcβ1-4(Fucα1-6)GlcNAcβ-Sp24	37	32
587	449	Neu5Acα2-8Neu5Acα2-3Galβ1-3GalNAcβ1-4(Neu5Acα2-8Neu5Acα2-3)Galβ1-4Glcβ-Sp0	28	33
588	487	Neu5Acα2-3Galβ1-3GlcNAcβ1-6GalNAcα-Sp14	24	11
589	165	Galβ1-4GlcNAcβ1-3Galβ1-4Glcβ-Sp8	15	49
590	224	Neu5Acα2-3Galβ1-3GalNAcα-Sp14	11	76
591	327	Neu5Acα2-6Galβ1-4GlcNAcβ1-3Galβ1-3GlcNAcβ-Sp0	8	68
592	441	(6S)Galβ1-3(6S)GlcNAc-Sp0	8	15
593	201	GlcAα-Sp8	3	134
594	340	GlcNAcα1-4Galβ1-4GlcNAcβ1-3Galβ1-4GlcNAcβ-Sp0	-1	71
595	510	GalNAcβ1-4(6S)GlcNAc-Sp8	-7	68
596	142	Galβ1-3GalNAcβ-Sp8	-15	23
597	274	Neu5Acα2-8Neu5Acα2-3Galβ1-4Glcβ-Sp0	-24	6
598	259	Neu5Acα2-3Galβ1-4GlcNAcβ1-3Galβ1-4GlcNAcβ-Sp0	-63	49
599	565	GlcNAcβ1-3Galβ1-4GlcNAcβ1-2Manα1-6(GlcNAcβ1-3Galβ1-4GlcNAcβ1-2Manα1-3)Manβ1-4GlcNAcβ1-4(Fucα1-6)GlcNAcβ-Sp24	-78	86
600	211	Manα1-2Manα1-6(Manα1-3)Manα1-6(Manα1-2Manα1-2Manα1-3)Manβ1-4GlcNAcβ1-4GlcNAcβ-Sp12	-133	17
601	363	Neu5Acα2-6GlcNAcβ1-4GlcNAc-Sp21	-197	84

Table S2. Glycan array analysis of mannose receptor CRD4 binding on Consortium for Functional Glycomics array version 6.2 screened at ~7.5 mM $CaCl_2$.

Rank	Glycan Number	Structure	Average RFU	Standard deviation
1	119	Galα1-4(Fucα1-2)Galβ1-4GlcNAcβ-Sp8	32602	430
2	207	Manα1-2Manα1-2Manα1-3Manα-Sp9	30095	2495
3	214	Manα1-2Manα1-2Manα1-6(Manα1-3)Manα-Sp9	26900	1201
4	78	Fucα1-2Galβ-Sp8	22666	776
5	93	GalNAcα1-4(Fucα1-2)Galβ1-4GlcNAcβ-Sp8	20626	2136
6	80	Fucα1-4GlcNAcβ-Sp8	19159	716
7	79	Fucα1-3GlcNAcβ-Sp8	19144	909
8	130	Fucα1-4(Galβ1-3)GlcNAcβ-Sp8	13915	2562
9	129	Galβ1-3(Fucα1-4)GlcNAc-Sp8	13280	2897
10	313	Manα1-2Manα1-6(Manα1-3)Manα1-6(Manα1-2Manα1-2Manα1-3)Manα-Sp9	13222	1077
11	314	Manα1-2Manα1-6(Manα1-2Manα1-3)Manα1-6(Manα1-2Manα1-2Manα1-3)Manα-Sp9	13213	1423
12	7	Fucα-Sp9	12321	1052
13	128	Galβ1-3(Fucα1-4)GlcNAc-Sp0	11387	1607
14	126	Galβ1-3(Fucα1-4)GlcNAcβ1-3Galβ1-4(Fucα1-3)GlcNAcβ-Sp0	10166	744
15	537	Fucα1-2Galβ1-4GlcNAcβ1-3Galβ1-4GlcNAcβ1-2Manα1-6(Fucα1-2Galβ1-4GlcNAcβ1-3Galβ1-4GlcNAcβ1-2Manα1-3)Manβ1-4GlcNAcβ1-4GlcNAcβ-Sp24	8747	826
16	60	Fucα1-2Galβ1-3(Fucα1-4)GlcNAcβ-Sp8	8341	1177
17	208	Manα1-2Manα1-6(Manα1-2Manα1-3)Manα-Sp9	7982	1957
18	488	(3S)Galβ1-3(Fucα1-4)GlcNAcβ-Sp0	7279	842
19	215	Manα1-6(Manα1-3)Manα1-6(Manα1-2Manα1-3)Manβ1-4GlcNAcβ1-4GlcNAcβ-Sp12	6442	369
20	90	GalNAcα1-3(Fucα1-2)Galβ-Sp18	5960	1179
21	471	GlcNAcβ1-6(GlcNAcβ1-2)Manα1-6(GlcNAcβ1-2Manα1-3)Manβ1-4GlcNAcβ1-4(Fucα1-6)GlcNAcβ-Sp24	5344	161
22	202	GlcAβ-Sp8	5325	172
23	28	(3S)Galβ1-3(Fucα1-4)GlcNAcβ-Sp8	4642	871
24	470	Fucα1-2Galβ1-3(Fucα1-4)GlcNAcβ1-2Manα1-6(Fucα1-2Galβ1-3(Fucα1-4)GlcNAcβ1-2Manα1-3)Manβ1-4GlcNAcβ1-4(Fucα1-6)GlcNAcβ1-4(Fucα1-6)GlcNAcβ-Sp19	4407	218
25	275	Galβ1-3(Fucα1-4)GlcNAcβ1-3Galβ1-3(Fucα1-4)GlcNAcβ-Sp0	4127	84
26	540	Galβ1-4GlcNAcβ1-3Galβ1-4GlcNAcβ1-3Galβ1-4GlcNAcβ1-2Manα1-6(Galβ1-4GlcNAcβ1-3Galβ1-4GlcNAcβ1-3Galβ1-4GlcNAcβ1-2Manα1-3)Manβ1-4GlcNAcβ1-4GlcNAcβ-Sp12	4037	425
27	503	GlcNAcβ1-6(GlcNAcβ1-2)Manα1-6(GlcNAcβ1-4)(GlcNAcβ1-4(GlcNAcβ1-2)Manα1-3)Manβ1-4GlcNAcβ1-4(Fucα1-6)GlcNAc-Sp21	4017	125
28	598	Neu5Acα2-3Galβ1-4GlcNAcβ1-3Galβ1-4GlcNAcβ1-3Galβ1-4GlcNAcβ1-2Manα1-6(Neu5Acα2-3Galβ1-4GlcNAcβ1-3Galβ1-4GlcNAcβ1-3Galβ1-4GlcNAcβ1-2Manα1-3)Manβ1-4GlcNAcβ1-4GlcNAcβ-Sp12	3955	364
29	217	Manβ1-4GlcNAcβ-Sp0	3858	544
30	76	Fucα1-2Galβ1-4GlcNAcβ-Sp8	3787	469
31	89	GalNAcα1-3(Fucα1-2)Galβ-Sp8	3688	127
32	209	Manα1-2Manα1-3Manα-Sp9	3635	388
33	597	Neu5Acα2-6Galβ1-4GlcNAcβ1-3Galβ1-4GlcNAcβ1-3Galβ1-4GlcNAcβ1-2Manα1-6(Neu5Acα2-6Galβ1-4GlcNAcβ1-3Galβ1-4GlcNAcβ1-3Galβ1-4GlcNAcβ1-2Manα1-3)Manβ1-4GlcNAcβ1-4GlcNAcβ-Sp12	3592	209
34	358	Fucα1-2Galβ1-4GlcNAcβ1-2Manα1-6(Fucα1-2Galβ1-4GlcNAcβ1-2Manα1-3)Manβ1-4GlcNAcβ1-4GlcNAcβ-Sp20	3495	255
35	188	GlcNAcβ1-4Galβ1-4GlcNAcβ-Sp8	3399	211
36	213	Manα1-6(Manα1-3)Manα-Sp9	3243	175
37	534	Galβ1-3GalNAcβ1-3Gal-Sp21	2991	471
38	382	Galβ1-4(Fucα1-3)GlcNAcβ1-6(Fucα1-4(Fucα1-2Galβ1-3)GlcNAcβ1-3)Galβ1-4Glc-Sp21	2939	127
39	418	GlcNAcβ1-2(GlcNAcβ1-6)Manα1-6(GlcNAcβ1-2Manα1-3)Manβ1-4GlcNAcβ1-4GlcNAcβ-Sp19	2926	99
40	73	Fucα1-2Galβ1-4GlcNAcβ1-3Galβ1-4GlcNAcβ-Sp0	2788	500
41	247	Fucα1-2(6S)Galβ1-4Glcβ-Sp0	2764	166
42	95	GalNAcβ1-3(Fucα1-2)Galβ-Sp8	2741	259
43	535	GlcNAcβ1-3Galβ1-4GlcNAcβ1-2Manα1-6(GlcNAcβ1-3Galβ1-4GlcNAcβ1-2Manα1-3)Manβ1-4GlcNAcβ1-4GlcNAcβ-Sp12	2693	323
44	75	Fucα1-2Galβ1-4GlcNAcβ-Sp0	2508	539
45	81	Fucβ1-3GlcNAcβ-Sp8	2455	23
46	599	Neu5Acα2-6Galβ1-4GlcNAcβ1-3Galβ1-4GlcNAcβ1-2Manα1-6(Neu5Acα2-6Galβ1-4GlcNAcβ1-3Galβ1-4GlcNAcβ1-2Manα1-3)Manβ1-4GlcNAcβ1-4GlcNAcβ-Sp12	2420	485
47	178	GlcNAcβ1-6(GlcNAcβ1-3)Galβ1-4GlcNAcβ-Sp8	2351	154
48	21	GlcNAcβ1-6(GlcNAcβ1-4)(GlcNAcβ1-3)GlcNAc-Sp8	2326	58
49	187	GlcNAcβ1-6(GlcNAcβ1-4)GalNAcα-Sp8	2319	295

50	531	GlcNAcβ1-3Galβ1-4GlcNAcβ1-6(GlcNAcβ1-3)Galβ1-4GlcNAc-Sp0	2296	317
51	367	GalNAcα1-3(Fucα1-2)Galβ1-4GlcNAcβ1-2Manα1-6(GalNAcα1-3(Fucα1-2)Galβ1-4GlcNAcβ1-2Manα1-3)Manβ1-4GlcNAcβ1-4GlcNAcβ-Sp20	2294	384
52	600	GlcNAcβ1-3Fucα-Sp21	2162	80
53	385	GlcNAcβ1-2Manα1-6(GlcNAcβ1-4(GlcNAcβ1-2)Manα1-3)Manβ1-4GlcNAcβ1-4GlcNAc-Sp21	2060	158
54	536	GlcNAcβ1-3Galβ1-4GlcNAcβ1-2Manα1-6(GlcNAcβ1-3Galβ1-4GlcNAcβ1-2Manα1-3)Manβ1-4GlcNAcβ1-4GlcNAcβ-Sp25	2057	672
55	50	Manα1-6(Manα1-3)Manβ1-4GlcNAcβ1-4GlcNAcβ-Sp12	1990	245
56	108	Galα1-3(Fucα1-2)Galβ-Sp18	1964	138
57	346	Galβ1-4GlcNAcβ1-2Manα1-3Manβ1-4GlcNAcβ1-4GlcNAc-Sp12	1953	275
58	77	Fucα1-2Galβ1-4Glcβ-Sp0	1944	269
59	107	Galα1-3(Fucα1-2)Galβ-Sp8	1895	125
60	167	Galβ1-4GlcNAcβ1-6(Galβ1-3)GalNAc-Sp14	1882	1828
61	538	GlcNAcβ1-3Galβ1-4GlcNAcβ1-3Galβ1-4GlcNAcβ1-2Manα1-6(GlcNAcβ1-3Galβ1-4GlcNAcβ1-3Galβ1-4GlcNAcβ1-2Manα1-3)Manβ1-4GlcNAcβ1-4GlcNAcβ-Sp12	1861	55
62	469	Fucα1-2Galβ1-4(Fucα1-3)GlcNAcβ1-2Manα1-6(Fucα1-2Galβ1-4(Fucα1-3)GlcNAcβ1-2Manα1-3)Manβ1-4GlcNAcβ1-4(Fucα1-6)GlcNAcβ-Sp24	1844	127
63	549	GlcNAcβ1-3Galβ1-4GlcNAcβ1-6(GlcNAcβ1-3Galβ1-4GlcNAcβ1-2)Manα1-6(GlcNAcβ1-3Galβ1-4GlcNAcβ1-2Man a1-3)Manβ1-4GlcNAcβ1-4GlcNAc-Sp24	1837	222
64	216	Manα1-6(Manα1-3)Manα1-6(Manα1-3)Manβ1-4GlcNAcβ1-4 GlcNAcβ-Sp12	1835	139
65	483	Neu5Acα2-6Galβ1-4GlcNAcβ1-6(Fucα1-2Galβ1-4(Fucα1-3)GlcNAcβ1-3)Galβ1-4Glc-Sp21	1819	309
66	567	GlcNAcβ1-3Galβ1-4GlcNAcβ1-3Galβ1-4GlcNAcβ1-2Manα1-6(GlcNAcβ1-3Galβ1-4GlcNAcβ1-3Galβ1-4GlcNAcβ1-2Manα1-3)Manβ1-4GlcNAcβ1-4(Fucα1-6)GlcNAcβ-Sp24	1786	209
67	486	Galβ1-3(Fucα1-4)GlcNAcβ1-6GalNAcα-Sp14	1782	174
68	248	Neu5Acα2-3Galβ1-3GlcNAcβ-Sp0	1752	141
69	310	MurNAcβ1-4GlcNAcβ-Sp10	1729	624
70	74	Fucα1-2Galβ1-4GlcNAcβ1-3Galβ1-4GlcNAcβ1-3Galβ1-4GlcNAcβ-Sp0	1720	221
71	297	Neu5Acα2-3Galβ1-4(Fucα1-3)GlcNAcβ1-6(Galβ1-3)GalNAcα-Sp14	1709	230
72	480	Manα1-6(Manα1-3)Manβ1-4GlcNAcβ1-4(Fucα1-6)GlcNAcβ-Sp19	1709	66
73	237	Neu5Acα2-3Galβ1-3(6S)GlcNAc-Sp8	1696	95
74	308	GlcNAcβ1-4GlcNAcβ-Sp10	1651	74
75	220	Fucα1-2(6S)Galβ1-4GlcNAcβ-Sp0	1620	155
76	51	Manα1-6(Manα1-3)Manβ1-4GlcNAcβ1-4GlcNAcβ-Sp13	1615	159
77	53	GlcNAcβ1-2Manα1-6(GlcNAcβ1-2Manα1-3)Manβ1-4GlcNAcβ1-4GlcNAcβ-Sp13	1603	139
78	312	Manα1-6(Manα1-3)Manα1-6(Manα1-3)Manβ-Sp10	1503	136
79	321	Galβ1-3GlcNAcβ1-2Manα1-6(Galβ1-3GlcNAcβ1-2Manα1-3)Manβ1-4GlcNAcβ1-4GlcNAcβ-Sp19	1499	358
80	102	Galα1-3(Fucα1-2)Galβ1-3GlcNAcβ-Sp8	1470	57
81	347	Galβ1-4GlcNAcβ1-2Manα1-6Manβ1-4GlcNAcβ1-4GlcNAc-Sp12	1470	140
82	446	Fucα1-2Galβ1-4GlcNAcβ1-6(Fucα1-2Galβ1-4GlcNAcβ1-3)GalNAc-Sp14	1464	126
83	52	GlcNAcβ1-2Manα1-6(GlcNAcβ1-2Manα1-3)Manβ1-4GlcNAcβ1-4GlcNAcβ-Sp12	1423	83
84	83	GalNAcα1-3(Fucα1-2)Galβ1-4(Fucα1-3)GlcNAcβ-Sp0	1412	56
85	183	GlcNAcβ1-3Galβ1-4GlcNAcβ-Sp8	1405	45
86	176	GlcNAcβ1-6(GlcNAcβ1-3)GalNAcα-Sp8	1391	54
87	401	Galα1-4Galβ1-4GlcNAcβ1-2Manα1-6(Galα1-4Galβ1-4GlcNAcβ1-2Manα1-3)Manβ1-4GlcNAcβ1-4GlcNAcβ-Sp24	1388	1641
88	84	(3S)Galβ1-4(Fucα1-3)Glcβ-Sp0	1375	92
89	173	GlcNAcα1-3Galβ1-4GlcNAcβ-Sp8	1368	142
90	71	Fucα1-2Galβ1-4(Fucα1-3)GlcNAcβ-Sp0	1356	211
91	594	Neu5Acα2-3Galβ1-4GlcNAcβ1-3Galβ1-4GlcNAcβ1-2Manα1-6(Neu5Acα2-3Galβ1-4GlcNAcβ1-3Galβ1-4GlcNAcβ1-2Manα1-3)Manβ1-4GlcNAcβ1-4GlcNAcβ-Sp12	1355	177
92	562	Galβ1-3GlcNAcβ1-3Galβ1-4GlcNAcβ1-3Galβ1-4GlcNAcβ1-6(Galβ1-3GlcNAcβ1-3Galβ1-4GlcNAcβ1-3Galβ1-4GlcNAβ1-2)Manα1-6(Galβ1-3GlcNAcβ1-3Galβ1-4GlcNAcβ1-3Galβ1-4GlcNAcβ1-2Manα1-3)Manβ1-4GlcNAcβ1-4(Fucα1-6)GlcNAcβ-Sp24	1349	539
93	345	Neu5Acα2-6Galβ1-4GlcNAcβ1-2Manα1-3Manβ1-4GlcNAcβ1-4GlcNAc-Sp12	1300	298
94	555	GlcNAcβ1-3Galβ1-4GlcNAcβ1-3Galβ1-4GlcNAcβ1-3Galβ1-4GlcNAcβ1-3Galβ1-4GlcNAcβ1-2Manα1-6(GlcNAcβ1-3Galβ1-4GlcNAcβ1-3Galβ1-4GlcNAcβ1-3Galβ1-4GlcNAcβ1-3Galβ1-4GlcNAcβ1-2Manα1-3)Manβ1-4GlcNAcβ1-4GlcNAcβ-Sp25	1281	130
95	368	Galα1-3(Fucα1-2)Galβ1-4GlcNAcβ1-2Manα1-6(Galα1-3(Fucα1-2)Galβ1-4GlcNAcβ1-2Manα1-3)Manβ1-4GlcNAcβ1-4GlcNAcβ-Sp20	1271	139
96	47	(6S)GlcNAcβ-Sp8	1249	166
97	177	GlcNAcβ1-6(GlcNAcβ1-3)GalNAcα-Sp14	1225	163
98	307	GlcNAcβ1-3Man-Sp10	1215	184
99	191	GlcNAcβ1-4GlcNAcβ1-4GlcNAcβ-Sp8	1211	106
100	290	Galβ1-4(Fucα1-3)GlcNAcβ1-3Galβ1-3(Fucα1-4)GlcNAcβ-Sp0	1177	94
101	111	Galα1-3GalNAcα-Sp16	1166	114
102	489	Galβ1-4(Fucα1-3)GlcNAcβ1-6(Neu5Acα2-6(Neu5Acα2-3Galβ1-3)GlcNAcβ1-3)Galβ1-4Glc-Sp21	1155	116
103	349	GlcNAcβ1-2Manα1-6(GlcNAcβ1-2Manα1-3)Manβ1-4GlcNAcβ1-4(Fucα1-6)GlcNAcβ-Sp22	1152	54

104	192	GlcNAcβ1-6GalNAcα-Sp8	1150	174
105	395	Galβ1-4GlcNAcβ1-2Manα1-6(GlcNAcβ1-2Manα1-3)Manβ1-4GlcNAcβ1-4GlcNAc-Sp12	1138	645
106	70	Fucα1-2Galβ1-4(Fucα1-3)GlcNAcβ1-3Galβ1-4(Fucα1-3)GlcNAcβ1-3Galβ1-4(Fucα1-3)GlcNAcβ-Sp0	1129	21
107	72	Fucα1-2Galβ1-4(Fucα1-3)GlcNAcβ-Sp8	1126	28
108	88	GlcNAcβ1-3Galβ1-3GalNAcα-Sp8	1114	47
109	309	GlcNAcβ1-4GlcNAcβ-Sp12	1110	212
110	182	GlcNAcβ1-3Galβ1-4GlcNAcβ-Sp0	1104	122
111	194	GlcNAcβ1-6Galβ1-4GlcNAcβ-Sp8	1094	98
112	415	GalNAcα1-3(Fucα1-2)Galβ1-4(Fucα1-3)GlcNAcβ1-3GalNAc-Sp14	1090	1387
113	348	Manα1-6(Galβ1-4GlcNAcβ1-2Manα1-3)Manβ1-4GlcNAcβ1-4GlcNAcβ-Sp12	1081	39
114	427	Fucα1-3GlcNAcβ1-6(Galβ1-4GlcNAcβ1-3)Galβ1-4Glc-Sp21	1081	164
115	69	Fucα1-2Galβ1-4(Fucα1-3)GlcNAcβ1-3Galβ1-4(Fucα1-3)GlcNAcβ-Sp0	1079	65
116	552	GlcNAcβ1-3Galβ1-4GlcNAcβ1-6(GlcNAcβ1-3Galβ1-3)GalNAcα-Sp14	1071	233
117	466	Glcα1-6Glcα1-6Glcα1-6Glcβ-Sp10	1054	205
118	17	GlcNAcβ-Sp8	1051	26
119	431	GlcNAcβ1-6(GlcNAcβ1-2)Manα1-6(GlcNAcβ1-4)(GlcNAcβ1-4(GlcNAcβ1-2)Manα1-3)Manβ1-4GlcNAcβ1-4GlcNAc-Sp21	1046	127
120	381	Galβ1-4GlcNAcβ1-6(Fucα1-4(Fucα1-2Galβ1-3)GlcNAcβ1-3)Galβ1-4Glc-Sp21	1040	167
121	451	GalNAcα1-3(Fucα1-2)Galβ1-4GlcNAcβ1-2Manα1-6(GalNAcα1-3(Fucα1-2)Galβ1-4GlcNAcβ1-2Manα1-3)Manβ1-4GlcNAcβ1-4(Fucα1-6)GlcNAcβ-Sp22	1037	113
122	360	Galα1-3Galβ1-4GlcNAcβ1-2Manα1-6(Galα1-3Galβ1-4GlcNAcβ1-2Manα1-3)Manβ1-4GlcNAcβ1-4GlcNAcβ-Sp20	1023	146
123	400	Galα1-4Galβ1-3GlcNAcβ1-2Manα1-6(Galα1-4Galβ1-3GlcNAcβ1-2Manα1-3)Manβ1-4GlcNAcβ1-4GlcNAcβ-Sp19	1017	51
124	181	GlcNAcβ1-3Galβ-Sp8	1007	32
125	344	Neu5Acα2-6Galβ1-4GlcNAcβ1-2Manα1-6Manβ1-4GlcNAcβ1-4GlcNAc-Sp12	1001	11
126	526	GlcNAcβ1-2 Manα1-6(GlcNAcβ1-4)(GlcNAcβ1-2Manα1-3)Manβ1-4GlcNAcβ1-4(Fucα1-6)GlcNAc-Sp21	985	181
127	61	Fucα1-2Galβ1-3GalNAcα-Sp8	975	217
128	582	Galβ1-4GlcNAcβ1-3Galβ1-4GlcNAcβ1-6(Galβ1-4GlcNAcβ1-3Galβ1-4GlcNAcβ1-3)GalNAcα-Sp14	971	296
129	197	Glcα1-6Glcα1-6Glcβ-Sp8	959	175
130	185	GlcNAcβ1-3Galβ1-4Glcβ-Sp0	938	118
131	490	Fucα1-2Galβ1-4GlcNAcβ1-6GalNAcα-Sp14	925	78
132	556	Galβ1-4GlcNAcβ1-3Galβ1-4GlcNAcβ1-3Galβ1-4GlcNAcβ1-3Galβ1-4GlcNAcβ1-3Galβ1-4GlcNAcβ1-2Manα1-6(Galβ1-4GlcNAcβ1-3Galβ1-4GlcNAcβ1-3Galβ1-4GlcNAcβ1-3Galβ1-4GlcNAcβ1-3Galβ1-4GlcNAcβ1-2Manα1-3)Manβ1-4GlcNAcβ1-4GlcNAcβ-Sp25	920	405
133	289	Galβ1-4(Fucα1-3)(6S)Glcβ-Sp0	900	172
134	311	Manα1-6Manβ-Sp10	896	137
135	417	Fucα1-2Galβ1-4GlcNAcβ1-2Manα1-6(Fucα1-2Galβ1-4GlcNAcβ1-2Manα1-3)Manβ1-4GlcNAcβ1-4(Fucα1-6)GlcNAcβ-Sp22	896	69
136	104	Galα1-3(Fucα1-2)Galβ1-4(Fucα1-3)GlcNAcβ-Sp8	882	65
137	278	Neu5Gcα2-3Galβ1-3(Fucα1-4)GlcNAcβ-Sp0	870	90
138	179	GlcNAcβ1-3GalNAcα-Sp8	862	65
139	580	Galβ1-4GlcNAcβ1-3Galβ1-4GlcNAcβ1-3GalNAcα-Sp14	858	113
140	563	Galβ1-3GlcNAcβ1-3Galβ1-4GlcNAcβ1-6(Galβ1-3GlcNAcβ1-3Galβ1-4GlcNAβ1-2)Manα1-6(Galβ1-3GlcNAcβ1-3Galβ1-4GlcNAcβ1-2Manα1-3)Manβ1-4GlcNAcβ1-4(Fucα1-6)GlcNAcβ-Sp24	851	202
141	222	Fucα1-2(6S)Galβ1-4(6S)Glcβ-Sp0	847	53
142	467	Glcα1-4Glcα1-4Glcα1-4Glcβ-Sp10	825	20
143	356	KDNα2-3Galβ1-3GalNAcα-Sp14	806	451
144	389	GalNAcβ1-4(Neu5Acα2-3)Galβ1-4GlcNAcβ1-3GalNAcα-Sp14	804	57
145	332	GalNAcα1-3(Fucα1-2)Galβ1-4GlcNAcβ1-3Galβ1-4GlcNAcβ-Sp0	800	151
146	343	Manα1-6(Neu5Acα2-6Galβ1-4GlcNAcβ1-2Manα1-3)Manβ1-4GlcNAcβ1-4GlcNAc-Sp12	791	144
147	362	Fucα1-4(Galβ1-3)GlcNAcβ1-2Manα1-6(Fucα1-4(Galβ1-3)GlcNAcβ1-2Manα1-3)Manβ1-4GlcNAcβ1-4(Fucα1-6)GlcNAcβ-Sp22	789	58
148	328	Neu5Acα2-3Galβ1-3(Fucα1-4)GlcNAcβ1-3Galβ1-3(Fucα1-4)GlcNAcβ-Sp0	787	34
149	190	GlcNAcβ1-4GlcNAcβ1-4GlcNAcβ1-4GlcNAcβ1-4GlcNAcβ1-Sp8	781	86
150	361	Galβ1-4GlcNAcβ1-2Manα1-6(Manα1-3)Manβ1-4GlcNAcβ1-4GlcNAcβ-Sp12	765	40
151	18	GlcN(Gc)β-Sp8	763	59
152	16	GlcNAcβ-Sp0	746	61
153	260	Fucα1-2Galβ1-4(6S)Glcβ-Sp0	744	824
154	133	GlcNAcβ1-6(Galβ1-3)GalNAcα-Sp8	738	137
155	429	GlcNAcβ1-2Manα1-6(GlcNAcβ1-4)(GlcNAcβ1-4(GlcNAcβ1-2)Manα1-3)Manβ1-4GlcNAcβ1-4GlcNAc-Sp21	737	47
156	196	Glcα1-4Glcα-Sp8	728	21
157	394	GlcNAcβ1-2Manα1-6(Galβ1-4GlcNAcβ1-2Manα1-3)Manβ1-4GlcNAcβ1-4GlcNAc-Sp12	719	77
158	301	GlcNAcβ1-6(Galβ1-4GlcNAcβ1-3)Galβ1-4GlcNAc-Sp0	717	74
159	430	GlcNAcβ1-6(GlcNAcβ1-2)Manα1-6(GlcNAcβ1-4)(GlcNAcβ1-2Manα1-3)Manβ1-4GlcNAcβ1-4GlcNAc-Sp21	716	54

160	186	GlcNAcβ1-4-MDPLys	713	119
161	397	Fucα1-2Galβ1-4GlcNAcβ1-3GalNAcα-Sp14	700	32
162	522	Neu5Acα2-3Galβ1-3GlcNAcβ1-2Manα-Sp0	693	961
163	406	Galα1-3(Fucα1-2)Galβ1-4(Fucα1-3)Glcβ-Sp21	682	364
164	542	Galβ1-3GlcNAcβ1-3Galβ1-4GlcNAcβ1-2Manα1-6(Galβ1-3GlcNAcβ1-3Galβ1-4GlcNAcβ1-2Manα1-3)Manβ1-4GlcNAcβ1-4GlcNAc-Sp25	679	139
165	221	Fucα1-2Galβ1-4(6S)GlcNAcβ-Sp8	677	106
166	94	GalNAcβ1-3GalNAcα-Sp8	673	103
167	127	Galβ1-3GlcNAcβ1-3Galβ1-4(Fucα1-3)GlcNAcβ-Sp0	672	12
168	193	GlcNAcβ1-6GalNAcα-Sp14	671	123
169	238	Neu5Acα2-3Galβ1-3(Fucα1-4)GlcNAcβ-Sp8	669	119
170	54	Galβ1-4GlcNAcβ1-2Manα1-6(Galβ1-4GlcNAcβ1-2Manα1-3)Manβ1-4GlcNAcβ1-4GlcNAcβ-Sp12	663	65
171	245	Neu5Acα2-3Galβ1-3GalNAcβ1-3Galα1-4Galβ1-4Glcβ-Sp0	639	61
172	324	Galβ1-4(Fucα1-3)GlcNAcβ1-2Manα1-6(Galβ1-4(Fucα1-3)GlcNAcβ1-2Manα1-3)Manβ1-4GlcNAcβ1-4GlcNAcβ-Sp20	634	78
173	424	Galα1-3(Fucα1-2)Galβ1-4GlcNAcβ1-2Manα1-6(Galα1-3(Fucα1-2)Galβ1-4GlcNAcβ1-2Manα1-3)Manβ1-4GlcNAcβ1-4(Fucα1-6)GlcNAcβ-Sp22	618	18
174	184	GlcNAcβ1-3Galβ1-4GlcNAcβ1-3Galβ1-4GlcNAcβ-Sp0	616	36
175	391	Galα1-3Galβ1-3GlcNAcβ1-2Manα1-6(Galα1-3Galβ1-3GlcNAcβ1-2Manα1-3)Manβ1-4GlcNAcβ1-4GlcNAc-Sp19	606	90
176	519	GalNAcα1-3(Fucα1-2)Galβ1-4 GlcNAcβ1-2Manα-Sp0	599	50
177	455	Galβ1-4GlcNAcβ1-6(Galβ1-4GlcNAcβ1-2)Manα1-6(Galβ1-4GlcNAcβ1-2Manα1-3)Manβ1-4GlcNAcβ1-4GlcNAcβ-Sp19	598	62
178	195	Glcα1-4Glcβ-Sp8	594	142
179	286	Neu5Acα2-3Galβ1-4GlcNAcβ1-6(Galβ1-3)GalNAcα-Sp14	592	496
180	189	GlcNAcβ1-4GlcNAcβ1-4GlcNAcβ1-4GlcNAcβ1-4GlcNAcβ1-4GlcNAcβ1-Sp8	589	38
181	478	Neu5Acα2-6Galβ1-4GlcNAcβ1-2Manα1-6(Neu5Acα2-6Galβ1-4GlcNAcβ1-2Manα1-3)Manβ1-4GlcNAcβ1-4(Fucα1-6)GlcNAcβ-Sp24	586	39
182	11	Neu5Acβ-Sp8	584	271
183	161	Galβ1-4GlcNAcβ1-3Galβ1-4(Fucα1-3)GlcNAcβ1-3Galβ1-4(Fucα1-3)GlcNAcβ-Sp0	582	14
184	570	Galβ1-4GlcNAcβ1-3Galβ1-4GlcNAcβ1-3Galβ1-4GlcNAcβ1-3Galβ1-4GlcNAcβ1-2Manα1-6(Galβ1-4GlcNAcβ1-3Galβ1-4GlcNAcβ1-3Galβ1-4GlcNAcβ1-3Galβ1-4GlcNAcβ1-2Manα1-3)Manβ1-4GlcNAcβ1-4(Fucα1-6)GlcNAcβ-Sp24	582	455
185	66	Fucα1-2Galβ1-3GlcNAcβ1-3Galβ1-4Glcβ-Sp10	581	131
186	58	Fucα1-2Galβ1-3GalNAcβ1-3Galα-Sp9	576	83
187	342	Neu5Acα2-6Galβ1-4GlcNAcβ1-2Manα1-6(Manα1-3)Manβ1-4GlcNAcβ1-4GlcNAc-Sp12	561	67
188	481	Galβ1-4GlcNAcβ1-6(Galβ1-4GlcNAcβ1-2)Manα1-6(Galβ1-4GlcNAcβ1-2Manα1-3)Manβ1-4GlcNAcβ1-4(Fucα1-6)GlcNAcβ-Sp24	561	28
189	306	Neu5Acα2-6Galβ1-4GlcNAcβ1-2Manα1-6(GlcNAcβ1-2Manα1-3)Manβ1-4GlcNAcβ1-4GlcNAcβ-Sp12	557	13
190	24	(3S)Galβ1-4(Fucα1-3)(6S)Glc-Sp0	552	87
191	226	GalNAcβ1-4(Neu5Acα2-8Neu5Acα2-8Neu5Acα2-3)Galβ1-4Glcβ-Sp0	551	70
192	584	GlcNAcβ1-3Galβ1-4GlcNAcβ1-3GalNAcα-Sp14	550	100
193	333	GalNAcα1-3(Fucα1-2)Galβ1-4GlcNAcβ1-3Galβ1-4GlcNAcβ1-3Galβ1-4GlcNAcβ-Sp0	549	75
194	472	Galβ1-3GlcNAcβ1-2Manα1-6(GlcNAcβ1-4)(Galβ1-3GlcNAcβ1-2Manα1-3)Manβ1-4GlcNAcβ1-4GlcNAcβ-Sp21	548	60
195	351	Galβ1-3GlcNAcβ1-2Manα1-6(Galβ1-3GlcNAcβ1-2Manα1-3)Manβ1-4GlcNAcβ1-4(Fucα1-6)GlcNAcβ-Sp22	543	27
196	386	Fucα1-2Galβ1-3GalNAcα1-3(Fucα1-2)Galβ1-4Glcβ-Sp0	543	53
197	557	GlcNAβ1-3Galβ1-3GalNAc-Sp14	541	72
198	566	Galβ1-4GlcNAcβ1-3Galβ1-4GlcNAcβ1-2Manα1-6(Galβ1-4GlcNAcβ1-3Galβ1-4GlcNAcβ1-2Manα1-3)Manβ1-4GlcNAcβ1-4(Fucα1-6)GlcNAcβ-Sp24	540	23
199	454	GalNAcα1-3(Fucα1-2)Galβ1-3GlcNAcβ1-2Manα1-6(GalNAcα1-3(Fucα1-2)Galβ1-3GlcNAcβ1-2Manα1-3)Manβ1-4GlcNAcβ1-4(Fucα1-6)GlcNAcβ-Sp22	528	34
200	359	Fucα1-2Galβ1-4(Fucα1-3)GlcNAcβ1-2Manα1-6(Fucα1-2Galβ1-4(Fucα1-3)GlcNAcβ1-2Manα1-3)Manβ1-4GlcNAcβ1-4GlcNAβ-Sp20	527	10
201	372	Fucα1-4(Fucα1-2Galβ1-3)GlcNAcβ1-2Manα1-3(Fucα1-4(Fucα1-2Galβ1-3)GlcNAcβ1-2Manα1-3)Manβ1-4GlcNAcβ1-4GlcNAcβ-Sp19	523	62
202	32	(3S)Galβ1-4(Fucα1-3)GlcNAc-Sp0	522	28
203	581	Galβ1-4GlcNAcβ1-3Galβ1-4GlcNAcβ1-6(Galβ1-3)GalNAcα-Sp14	521	28
204	168	Galβ1-4GlcNAcβ-Sp0	517	67
205	239	Neu5Acα2-3Galβ1-3(Fucα1-4)GlcNAcβ1-3Galβ1-4(Fucα1-3)GlcNAcβ-Sp0	512	50
206	97	GalNAcβ1-4(Fucα1-3)GlcNAcβ-Sp0	505	115
207	106	Galα1-3(Fucα1-2)Galβ1-4Glcβ-Sp0	497	55
208	153	Galβ1-4(Fucα1-3)GlcNAcβ1-3Galβ1-4(Fucα1-3)GlcNAcβ-Sp0	488	19
209	152	Galβ1-4(Fucα1-3)GlcNAcβ-Sp8	487	30
210	180	GlcNAcβ1-3GalNAcα-Sp14	484	62
211	586	GlcNAcβ1-3Galβ1-4GlcNAcβ1-6(GlcNAcβ1-3Galβ1-4GlcNAcβ1-3)GalNAcα-Sp14	484	18

212	86	GalNAcα1-3(Fucα1-2)Galβ1-4GlcNAcβ-Sp8	481	55
213	442	Fucα1-2Galβ1-4 GlcNAcβ1-2Manα1-6(Fucα1-2Galβ1-4GlcNAcβ1-2(Fucα1-2Galβ1-4GlcNAcβ1-4)Manα1-3)Manβ1-4GlcNAcβ1-4GlcNAcβ-Sp12	481	12
214	428	GlcNAcβ1-2Manα1-6(GlcNAcβ1-4)(GlcNAcβ1-2Manα1-3)Manβ1-4GlcNAcβ1-4GlcNAc-Sp21	478	45
215	452	Galα1-3(Fucα1-2)Galβ1-3GlcNAcβ1-2Manα1-6(Galα1-3(Fucα1-2)Galβ1-3GlcNAcβ1-2Manα1-3)Manβ1-4GlcNAcβ1-4(Fucα1-6)GlcNAcβ-Sp22	478	56
216	20	Galβ1-4GlcNAcβ1-6(Galβ1-4GlcNAcβ1-3)GalNAc-Sp14	475	50
217	251	Neu5Acα2-3Galβ1-4(Fucα1-3)GlcNAcβ1-3Galβ1-4(Fucα1-3)GlcNAcβ1-3Galβ1-4(Fucα1-3)GlcNAcβ-Sp0	473	87
218	132	Galβ1-4GlcNAcβ1-6GalNAc-Sp14	465	29
219	85	GalNAcα1-3(Fucα1-2)Galβ1-4GlcNAcβ-Sp0	461	56
220	99	GalNAcβ1-4GlcNAcβ-Sp8	461	126
221	3	Manα-Sp8	455	33
222	199	Glcβ1-6Glcβ-Sp8	453	78
223	392	Galα1-3Galβ1-3(Fucα1-4)GlcNAcβ1-2Manα1-6(Galα1-3Galβ1-3(Fucα1-4)GlcNAcβ1-2Manα1-3)Manβ1-4GlcNAcβ1-4GlcNAc-Sp19	453	35
224	497	Fucα1-2(6S)Galβ1-3(6S)GlcNAcβ-Sp0	451	111
225	569	GlcNAcβ1-3Galβ1-4GlcNAcβ1-3Galβ1-4GlcNAcβ1-3Galβ1-4GlcNAcβ1-2Manα1-6(GlcNAcβ1-3Galβ1-4GlcNAcβ1-3Galβ1-4GlcNAcβ1-3Galβ1-4GlcNAcβ1-2Manα1-3)Manβ1-4GlcNAcβ1-4(Fucα1-6)GlcNAcβ-Sp24	448	287
226	425	Galβ1-3GlcNAcβ1-6(Galβ1-3GlcNAcβ1-2)Manα1-6(Galβ1-3GlcNAcβ1-2Manα1-3)Manβ1-4GlcNAcβ1-4GlcNAcβ-Sp19	443	152
227	154	Galβ1-4(Fucα1-3)GlcNAcβ1-3Galβ1-4(Fucα1-3)GlcNAcβ1-3Galβ1-4(Fucα1-3)GlcNAcβ-Sp0	442	50
228	448	GalNAcα1-3(Fucα1-2)Galβ1-4GlcNAcβ1-6(GalNAcα1-3(Fucα1-2)Galβ1-4GlcNAcβ1-3)GalNAc-Sp14	439	69
229	288	Galβ1-4(Fucα1-3)(6S)GlcNAcβ-Sp0	438	203
230	292	Neu5Acα2-3Galβ1-3GlcNAcβ1-3Galβ1-3GlcNAcβ-Sp0	437	125
231	369	Galα1-3Galβ1-4(Fucα1-3)GlcNAcβ1-2Manα1-6(Galα1-3Galβ1-4(Fucα1-3)GlcNAcβ1-2Manα1-3)Manβ1-4GlcNAcβ1-4GlcNAcβ-Sp20	431	16
232	573	Galβ1-4GlcNAcβ1-3Galβ1-4GlcNAcβ1-6(Galβ1-4GlcNAcβ1-3Galβ1-4GlcNAβ1-2)Manα1-6(Galβ1-4GlcNAcβ1-3Galβ1-4GlcNAcβ1-2Manα1-3)Manβ1-4GlcNAcβ1-4(Fucα1-6)GlcNAcβ-Sp24	428	32
233	479	Neu5Acα2-3Galβ1-4GlcNAcβ1-2Manα1-6(Neu5Acα2-3Galβ1-4GlcNAcβ1-2Manα1-3)Manβ1-4GlcNAcβ1-4(Fucα1-6)GlcNAcβ-Sp24	424	59
234	303	Galβ1-4GlcNAcβ1-6Galβ1-4GlcNAcβ-Sp0	419	40
235	350	Galβ1-4GlcNAcβ1-2Manα1-6(Galβ1-4GlcNAcβ1-2Manα1-3)Manβ1-4GlcNAcβ1-4(Fucα1-6)GlcNAcβ-Sp22	418	71
236	198	Glcβ1-4Glcβ-Sp8	414	36
237	287	Galβ1-3GlcNAcβ1-3Galβ1-3GlcNAcβ-Sp0	410	292
238	268	Neu5Acα2-6Galβ1-4GlcNAcβ1-3Galβ1-4(Fucα1-3)GlcNAcβ1-3Galβ1-4(Fucα1-3)GlcNAcβ-Sp0	408	56
239	65	Fucα1-2Galβ1-3GlcNAcβ1-3Galβ1-4Glcβ-Sp8	407	67
240	14	Manβ-Sp8	402	56
241	204	GlcAβ1-6Galβ-Sp8	402	64
242	112	Galα1-3GalNAcβ-Sp8	400	24
243	527	Galβ1-4GlcNAcβ1-2 Manα1-6(GlcNAcβ1-4)(Galβ1-4GlcNAcβ1-2Manα1-3)Manβ1-4GlcNAcβ1-4(Fucα1-6)GlcNAc-Sp21	396	4
244	426	Galβ1-4GlcNAcβ1-6(Fucα1-2Galβ1-3GlcNAcβ1-3)Galβ1-4Glc-Sp21	395	64
245	592	Neu5Acα2-6Galβ1-4GlcNAcβ1-3Galβ1-4GlcNAcβ1-6(Galβ1-3)GalNAcα-Sp14	390	145
246	143	Galβ1-3GalNAcβ1-3Galα1-4Galβ1-4Glcβ-Sp0	389	49
247	150	Galβ1-3GlcNAcβ-Sp8	386	7
248	296	(6P)Glcβ-Sp10	386	70
249	578	GlcNAcβ1-3Galβ1-4GlcNAcβ1-3Galβ1-4GlcNAcβ1-3Galβ1-4GlcNAcβ1-3Galβ1-4GlcNAcβ1-6(GlcNAcβ1-3Galβ1-4GlcNAcβ1-3Galβ1-4GlcNAcβ1-3Galβ1-4GlcNAcβ1-3Galβ1-4GlcNAβ1-2)Manα1-6(GlcNAcβ1-3Galβ1-4GlcNAcβ1-3Galβ1-4GlcNAcβ1-3Galβ1-4GlcNAcβ1-3Galβ1-4GlcNAcβ1-2Manα1-3)Manβ1-4GlcNAcβ1-4(Fucα1-6)GlcNAcβ-Sp24	385	505
250	284	Neu5Gcα2-6Galβ1-4GlcNAcβ-Sp0	384	59
251	423	Fucα1-2Galβ1-3GlcNAcβ1-2Manα1-6(Fucα1-2Galβ1-3GlcNAcβ1-2Manα1-3)Manβ1-4GlcNAcβ1-4(Fucα1-6)GlcNAcβ-Sp22	376	53
252	339	GlcNAcα1-4Galβ1-4GlcNAcβ1-3Galβ1-4(Fucα1-3)GlcNAcβ1-3Galβ1-4(Fucα1-3)GlcNAcβ-Sp0	375	57
253	299	Neu5Acα2-6Galβ1-4GlcNAcβ1-2Manα1-6(Galβ1-4GlcNAcβ1-2Manα1-3)Manβ1-4GlcNAcβ1-4GlcNAcβ-Sp12	372	27
254	59	Fucα1-2Galβ1-3GalNAcβ1-3Galα1-4Galβ1-4Glcβ-Sp9	366	64
255	585	GlcNAcβ1-3Galβ1-4GlcNAcβ1-6(Galβ1-3)GalNAcα-Sp14	365	53
256	317	Galβ1-4GlcNAcβ1-2Manα1-6(Neu5Acα2-6Galβ1-4GlcNAcβ1-2Manα1-3)Manβ1-4GlcNAcβ1-4GlcNAcβ-Sp12	361	30
257	67	Fucα1-2Galβ1-3GlcNAcβ-Sp0	358	39
258	551	Galα1-3Galβ1-4GlcNAcβ1-2Manα1-6(Galα1-3Galβ1-4GlcNAcβ1-2Manα1-3)Manβ1-4GlcNAcβ1-4GlcNAc-Sp24	353	18
259	323	Neu5Acα2-3Galβ1-4GlcNAcβ1-2Manα1-6(Neu5Acα2-6Galβ1-4GlcNAcβ1-2Manα1-3)Manβ1-4GlcNAcβ1-4GlcNAcβ-Sp12	352	113

260	87	GalNAcα1-3(Fucα1-2)Galβ1-4Glcβ-Sp0	351	141
261	103	Galα1-3(Fucα1-2)Galβ1-4(Fucα1-3)GlcNAcβ-Sp0	351	28
262	357	Fucα1-2Galβ1-3GlcNAcβ1-2Manα1-6(Fucα1-2Galβ1-3GlcNAcβ1-2Manα1-3)Manβ1-4GlcNAcβ1-4GlcNAcβ-Sp20	351	25
263	13	Glcβ-Sp8	348	108
264	337	GlcNAcα1-4Galβ1-3GlcNAcβ-Sp0	347	95
265	355	KDNα2-3Galβ1-4Glc-Sp0	347	73
266	57	Neu5Acα2-6Galβ1-4GlcNAcβ1-2Manα1-6(Neu5Acα2-6Galβ1-4GlcNAcβ1-2Manα1-3)Manβ1-4GlcNAcβ1-4GlcNAcβ-Sp24	345	88
267	365	Galβ1-4(Fucα1-3)GlcNAcβ1-6(Fucα1-2Galβ1-4GlcNAcβ1-3)Galβ1-4Glc-Sp21	336	66
268	496	Fucα1-2Galβ1-3(6S)GlcNAcβ-Sp0	333	10
269	375	Neu5Acα2-3Galβ1-4(Fucα1-3)GlcNAcβ1-3GalNAcα-Sp14	329	46
270	293	Neu5Acα2-3Galβ1-4GlcNAcβ1-3Galβ1-3GlcNAcβ-Sp0	323	139
271	322	Neu5Acα2-3Galβ1-4GlcNAcβ1-2Manα1-6(Neu5Acα2-3Galβ1-4GlcNAcβ1-2Manα1-3)Manβ1-4GlcNAcβ1-4GlcNAcβ-Sp12	323	51
272	175	GlcNAcβ1-2Galβ1-3GalNAcα-Sp8	309	28
273	572	Galβ1-4GlcNAcβ1-3Galβ1-4GlcNAcβ1-3Galβ1-4GlcNAcβ1-3Galβ1-4GlcNAcβ1-3Galβ1-4GlcNAcβ1-2Manα1-6(Galβ1-4GlcNAcβ1-3Galβ1-4GlcNAcβ1-3Galβ1-4GlcNAcβ1-3Galβ1-4GlcNAcβ1-3Galβ1-4GlcNAcβ1-2Manα1-3)Manβ1-4GlcNAcβ1-4(Fucα1-6)GlcNAcβ-Sp19	309	20
274	120	Galα1-4Galβ1-4GlcNAcβ-Sp0	307	59
275	62	Fucα1-2Galβ1-3GalNAcα-Sp14	306	61
276	68	Fucα1-2Galβ1-3GlcNAcβ-Sp8	306	67
277	151	Galβ1-4(Fucα1-3)GlcNAcβ-Sp0	306	20
278	101	Galα1-3(Fucα1-2)Galβ1-3GlcNAcβ-Sp0	304	36
279	533	Galα1-3(Fucα1-2)Galβ1-3GalNAcβ1-3Galα1-4Galβ1-4Glc-Sp21	303	114
280	136	Neu5Acα2-6(Galβ1-3)GalNAcα-Sp14	302	44
281	261	Neu5Acα2-3Galβ1-4Glcβ-Sp0	301	60
282	105	Galα1-3(Fucα1-2)Galβ1-4GlcNAc-Sp0	300	64
283	443	Fucα1-2Galβ1-4(Fucα1-3)GlcNAcβ1-2Manα1-6(Fucα1-2Galβ1-4(Fucα1-3)GlcNAcβ1-4(Fucα1-2Galβ1-4(Fucα1-3)GlcNAcβ1-2)Manα1-3)Manβ1-4GlcNAcβ1-4GlcNAcβ-Sp12	300	21
284	304	GalNAcβ1-3Galβ-Sp8	298	6
285	473	Neu5Acα2-6Galβ1-4GlcNAcβ1-6(Galβ1-3GlcNAcβ1-3)Galβ1-4Glcβ-Sp21	297	50
286	530	Neu5Acα2-3Galβ1-4(Fucα1-3)GlcNAcβ1-2Manα-Sp0	286	35
287	520	Galβ1-3GlcNAcβ1-2Manα-Sp0	281	62
288	145	Galβ1-3GalNAcβ1-4Galβ1-4Glcβ-Sp8	280	34
289	122	Galα1-4Galβ1-4Glcβ-Sp0	278	85
290	366	Galβ1-4GlcNAcβ1-2Manα1-6(Galβ1-4GlcNAcβ1-4(Galβ1-4GlcNAcβ1-2)Manα1-3)Manβ1-4GlcNAcβ1-4GlcNAc-Sp21	278	12
291	96	GalNAcβ1-3Galα1-4Galβ1-4GlcNAcβ-Sp0	271	230
292	325	Neu5,9Ac2α2-3Galβ1-4GlcNAcβ-Sp0	271	69
293	511	(3S)GalNAcβ1-4GlcNAc-Sp8	270	137
294	528	Galβ1-4GlcNAcβ1-2 Manα1-6(Galβ1-4GlcNAcβ1-4)(Galβ1-4GlcNAcβ1-2Manα1-3)Manβ1-4GlcNAcβ1-4(Fucα1-6)GlcNAc-Sp21	269	127
295	523	Galα1-3Galβ1-3GlcNAcβ1-2Manα-Sp0	268	141
296	174	GlcNAcα1-6Galβ1-4GlcNAcβ-Sp8	261	12
297	82	GalNAcα1-3(Fucα1-2)Galβ1-3GlcNAcβ-Sp0	260	54
298	271	Neu5Acα2-6Galβ1-4Glcβ-Sp8	258	41
299	131	Galβ1-4GlcNAcβ1-6GalNAcα-Sp8	257	149
300	200	G-ol-Sp8	256	88
301	35	(3S)Galβ1-4(6S)GlcNAcβ-Sp8	251	120
302	387	Fucα1-2Galβ1-3GalNAcα1-3(Fucα1-2)Galβ1-4GlcNAcβ-Sp0	250	52
303	205	KDNα2-3Galβ1-3GlcNAcβ-Sp0	248	32
304	589	GlcNAcβ1-3Galβ1-4GlcNAcβ1-3Galβ1-4GlcNAcβ1-3GalNAcα-Sp14	248	27
305	164	Galβ1-4GlcNAcβ1-3Galβ1-4Glcβ-Sp0	247	29
306	34	(3S)Galβ1-4(6S)GlcNAcβ-Sp0	244	43
307	336	GlcNAcα1-4Galβ1-4GlcNAcβ-Sp0	244	26
308	266	Neu5Acα2-6Galβ1-4GlcNAcβ-Sp0	242	91
309	376	GalNAcβ1-4GlcNAcβ1-2Manα1-6(GalNAcβ1-4GlcNAcβ1-2Manα1-3)Manβ1-4GlcNAcβ1-4GlcNAc-Sp12	240	104
310	444	Galβ1-4(Fucα1-3)GlcNAcβ1-6GalNAc-Sp14	240	29
311	447	Galα1-3(Fucα1-2)Galβ1-4GlcNAcβ1-6(Galα1-3(Fucα1-2)Galβ1-4GlcNAcβ1-3)GalNAc-Sp14	239	68
312	338	GlcNAcα1-4Galβ1-4GlcNAcβ1-3Galβ1-4Glcβ-Sp0	237	31
313	203	GlcAβ1-3Galβ-Sp8	236	33
314	380	Galβ1-4(Fucα1-3)GlcNAcβ1-6(Galβ1-3GlcNAcβ1-3)Galβ1-4Glc-Sp21	235	83
315	109	Galα1-4(Galα1-3)Galβ1-4GlcNAcβ-Sp8	234	52

316	571	GlcNAcβ1-3Galβ1-4GlcNAcβ1-3Galβ1-4GlcNAcβ1-3Galβ1-4GlcNAcβ1-3Galβ1-4GlcNAcβ1-2Manα1-6(GlcNAcβ1-3Galβ1-4GlcNAcβ1-3Galβ1-4GlcNAcβ1-3Galβ1-4GlcNAcβ1-3Galβ1-4GlcNAcβ1-2Manα1-3)Manβ1-4GlcNAcβ1-4(Fucα1-6)GlcNAcβ-Sp19	234	73
317	55	Neu5Acα2-6Galβ1-4GlcNAcβ1-2Manα1-6(Neu5Acα2-6Galβ1-4GlcNAcβ1-2Manα1-3)Manβ1-4GlcNAcβ1-4GlcNAcβ-Sp12	232	40
318	465	Galα1-3(Fucα1-2)Galβ1-3GalNAcβ-Sp8	232	92
319	319	Neu5Acα2-8Neu5Acα2-8Neu5Acβ-Sp8	230	64
320	272	Neu5Acα2-6Galβ-Sp8	229	39
321	371	Galα1-3(Fucα1-2)Galβ1-3GlcNAcβ1-2Manα1-6(Galcα1-3(Fucα1-2)Galβ1-3GlcNAcβ1-2Manα1-3)Manβ1-4GlcNAcβ1-4GlcNAcβ-Sp20	228	14
322	474	Neu5Acα2-3Galβ1-4GlcNAcβ1-2Manα-Sp0	225	57
323	468	Neu5Acα2-3Galβ1-4GlcNAcβ1-6(Neu5Acα2-3Galβ1-4GlcNAcβ1-3)GalNAcα-Sp14	221	64
324	212	Manα1-2Manα1-6(Manα1-2Manα1-3)Manα1-6(Manα1-2Manα1-2Manα1-3)Manβ1-4GlcNAcβ1-4GlcNAcβ-Sp12	219	21
325	476	Neu5Acα2-6Galβ1-4GlcNAcβ1-6GalNAcα-Sp14	217	138
326	501	Fucα1-2Galβ1-3GlcNAcβ1-6(Fucα1-2Galβ1-3GlcNAcβ1-3)GalNAcα-Sp14	216	54
327	33	(3S)Galβ1-4(Fucα1-3)GlcNAc-Sp8	215	19
328	315	Neu5Acα2-3Galβ1-4GlcNAcβ1-6(Neu5Acα2-3Galβ1-3)GalNAcα-Sp14	214	120
329	334	Neu5Acα2-3-Galβ1-4(Fucα1-3)GlcNAcβ1-6(Neu5Acα2-3Galβ1-3)GalNAc-Sp14	211	163
330	595	GlcNAcβ1-6(Neu5Acα2-3Galβ1-3)GalNAcα-Sp14	206	45
331	258	Neu5Acα2-3Galβ1-4GlcNAcβ-Sp8	205	95
332	281	Neu5Gcα2-3Galβ1-4GlcNAcβ-Sp0	203	36
333	411	GalNAcα1-3(Fucα1-2)Galβ1-4GlcNAcβ1-3GalNAcα-Sp14	200	77
334	114	Galα1-3Galβ1-3GlcNAcβ-Sp0	199	34
335	255	Neu5Acα2-3Galβ1-4(Fucα1-3)GlcNAcβ1-3Galβ1-4GlcNAcβ-Sp8	199	20
336	341	GlcNAcα1-4Galβ1-3GalNAc-Sp14	198	54
337	282	Neu5Gcα2-3Galβ1-4Glcβ-Sp0	196	26
338	44	(6S)Galβ1-4GlcNAcβ-Sp8	193	74
339	118	Galα1-3Galβ-Sp8	192	49
340	223	Neu5Acα2-3Galβ1-3GalNAcα-Sp8	192	56
341	250	Neu5Acα2-3Galβ1-4(Fucα1-3)(6S)GlcNAcβ-Sp8	192	60
342	141	Galβ1-3GalNAcα-Sp16	191	14
343	464	Galα1-3(Fucα1-2)Galβ1-3GalNAcα-Sp8	191	13
344	524	GalNAcβ1-4GlcNAcβ1-2Manα-Sp0	191	28
345	513	Galβ1-4(6P)GlcNAcβ-Sp0	190	68
346	113	Galα1-3Galβ1-4(Fucα1-3)GlcNAcβ-Sp8	189	75
347	225	GalNAcβ1-4(Neu5Acα2-8Neu5Acα2-8Neu5Acα2-8Neu5Acα2-3)Galβ1-4Glcβ-Sp0	189	46
348	19	Galβ1-4GlcNAcβ1-6(Galβ1-4GlcNAcβ1-3)GalNAcα-Sp8	188	40
349	243	Neu5Acα2-6(Neu5Acα2-3Galβ1-3)GalNAcα-Sp14	187	42
350	285	Neu5Gcα-Sp8	187	76
351	298	Galβ1-3Galβ1-4GlcNAcβ-Sp8	187	21
352	579	Galβ1-4GlcNAcβ1-3Galβ1-4GlcNAcβ1-3Galβ1-4GlcNAcβ1-3Galβ1-4GlcNAcβ1-3Galβ1-4GlcNAcβ1-6(Galβ1-4GlcNAcβ1-3Galβ1-4GlcNAcβ1-3Galβ1-4GlcNAcβ1-3Galβ1-4GlcNAcβ1-3Galβ1-4GlcNAβ1-2)Manα1-6(Galβ1-4GlcNAcβ1-3Galβ1-4GlcNAcβ1-3Galβ1-4GlcNAcβ1-3Galβ1-4GlcNAcβ1-3Galβ1-4GlcNAcβ1-2Manα1-3)Manβ1-4GlcNAcβ1-4(Fucα1-6)GlcNAcβ-Sp24	186	26
353	56	Neu5Acα2-6Galβ1-4GlcNAcβ1-2Manα1-6(Neu5Acα2-6Galβ1-4GlcNAcβ1-2Man-a1-3)Manβ1-4GlcNAcβ1-4GlcNAcβ-Sp21	185	16
354	453	Neu5Acα2-6Galβ1-4GlcNAcβ1-6(Fucα1-2Galβ1-3GlcNAcβ1-3)Galβ1-4Glc-Sp21	185	50
355	515	GalNAcα1-3(Fucα1-2)Galβ1-4GlcNAcβ1-6GalNAc-Sp14	185	27
356	601	Galβ1-3GalNAcβ1-4(Neu5Acα2-8Neu5Acα2-8Neu5Acα2-3)Galβ1-4Glcβ-Sp21	185	133
357	22	6S(3S)Galβ1-4(6S)GlcNAcβ-Sp0	184	155
358	354	KDNα2-6Galβ1-4GlcNAc-Sp0	184	88
359	419	Fucα1-2Galβ1-3GlcNAcβ1-3GalNAc-Sp14	184	54
360	487	Neu5Acα2-3Galβ1-3GlcNAcβ1-6GalNAcα-Sp14	183	132
361	518	Galα1-3(Fucα1-2)Galβ1-4GlcNAcβ1-2Manα-Sp0	183	12
362	158	Galβ1-4GalNAcβ1-3(Fucα1-2)Galβ1-4GlcNAcβ-Sp8	181	14
363	352	(6S)GlcNAcβ1-3Galβ1-4GlcNAcβ-Sp0	181	28
364	413	Fucα1-2Galβ1-4(Fucα1-3)GlcNAcβ1-3GalNAcα-Sp14	180	24
365	593	Neu5Acα2-6Galβ1-4GlcNAcβ1-6(Galβ1-3)GalNAcα-Sp14	179	80
366	156	Galβ1-4(6S)Glcβ-Sp8	173	32
367	37	(3S)Galβ1-4GlcNAcβ-Sp8	171	52
368	46	Neu5Acα2-3(6S)Galβ1-4GlcNAcβ-Sp8	171	86
369	110	Galα1-3GalNAcα-Sp8	170	50
370	280	Neu5Gcα2-3Galβ1-4(Fucα1-3)GlcNAcβ-Sp0	170	38
371	5	GalNAcα-Sp15	169	51
372	63	Fucα1-2Galβ1-3GalNAcβ1-4(Neu5Acα2-3)Galβ1-4Glcβ-Sp0	169	29
373	230	GalNAcβ1-4(Neu5Acα2-3)Galβ1-4GlcNAcβ-Sp0	166	15

374	294	4S(3S)Galβ1-4GlcNAcβ-Sp0	163	42
375	300	Galβ1-4GlcNAcβ1-6(Galβ1-4GlcNAcβ1-3)Galβ1-4GlcNAc-Sp0	163	41
376	331	GalNAcβ1-3Galα1-4Galβ1-4GlcNAcβ1-3Galβ1-4Glcβ-Sp0	163	95
377	270	Neu5Acα2-6Galβ1-4Glcβ-Sp0	162	20
378	577	Galβ1-4GlcNAcβ1-3Galβ1-4GlcNAcβ1-3Galβ1-4GlcNAcβ1-3Galβ1-4GlcNAcβ1-6(Galβ1-4GlcNAcβ1-3Galβ1-4GlcNAcβ1-3Galβ1-4GlcNAcβ1-3Galβ1-4GlcNAβ1-2)Manα1-6(Galβ1-4GlcNAcβ1-3Galβ1-4GlcNAcβ1-3Galβ1-4GlcNAcβ1-3Galβ1-4GlcNAcβ1-2Manα1-3)Manβ1-4GlcNAcβ1-4(Fucα1-6)GlcNAcβ-Sp24	162	32
379	554	GalNAcβ1-4GlcNAcβ1-3GalNAcβ1-4GlcNAcβ-Sp0	161	126
380	576	GlcNAcβ1-3Galβ1-4GlcNAcβ1-3Galβ1-4GlcNAcβ1-3Galβ1-4GlcNAcβ1-6(GlcNAcβ1-3Galβ1-4GlcNAcβ1-3Galβ1-4GlcNAcβ1-3Galβ1-4GlcNAβ1-2)Manα1-6(GlcNAcβ1-3Galβ1-4GlcNAcβ1-3Galβ1-4GlcNAcβ1-3Galβ1-4GlcNAcβ1-2Manα1-3)Manβ1-4GlcNAcβ1-4(Fucα1-6)GlcNAcβ-Sp24	161	59
381	291	Galβ1-4GlcNAcβ1-3Galβ1-3GlcNAcβ-Sp0	160	60
382	353	KDNα2-3Galβ1-4(Fucα1-3)GlcNAc-Sp0	160	40
383	140	Galβ1-3GalNAcα-Sp14	157	81
384	330	Galα1-4Galβ1-4GlcNAcβ1-3Galβ1-4Glcβ-Sp0	156	26
385	383	Galβ1-3GlcNAcβ1-3Galβ1-4(Fucα1-3)GlcNAcβ1-6(Galβ1-3GlcNAcβ1-3)Galβ1-4Glc-Sp21	153	66
386	510	GalNAcβ1-4(6S)GlcNAc-Sp8	153	181
387	12	Galβ-Sp8	152	49
388	596	Neu5Acα2-6Galβ1-4GlcNAcβ1-3Galβ1-4GlcNAcβ1-6(Neu5Acα2-6Galβ1-4GlcNAcβ1-3Galβ1-4GlcNAcβ1-3)GalNAcα-Sp14	152	65
389	91	GalNAcα1-3GalNAcβ-Sp8	151	4
390	320	Neu5Gcβ2-6Galβ1-4GlcNAc-Sp8	151	17
391	219	(3S)Galβ1-4(Fucα1-3)(6S)GlcNAcβ-Sp8	150	11
392	4	GalNAcα-Sp8	149	46
393	117	Galα1-3Galβ1-4Glc-Sp10	149	30
394	36	(3S)Galβ1-4GlcNAcβ-Sp0	148	60
395	43	(6S)Galβ1-4Glcβ-Sp8	147	76
396	206	KDNα2-3Galβ1-4GlcNAcβ-Sp0	147	27
397	8	Rhaα-Sp8	146	29
398	257	Neu5Acα2-3Galβ1-4GlcNAcβ-Sp0	146	13
399	100	Galα1-2Galβ-Sp8	144	63
400	227	Neu5Acα2-8Neu5Acα2-8Neu5Acα2-3Galβ1-4Glcβ-Sp0	144	41
401	1	Galα-Sp8	142	46
402	41	(6P)Manα-Sp8	142	58
403	160	Galβ1-4GlcNAcβ1-3GalNAc-Sp14	142	56
404	316	Neu5Acα2-6Galβ1-4GlcNAcβ1-2Manα1-6(Neu5Acα2-3Galβ1-4GlcNAcβ1-2Manα1-3)Manβ1-4GlcNAcβ1-4GlcNAcβ-Sp12	142	41
405	396	Neu5Acα2-3Galβ1-3GlcNAcβ1-3GalNAcα-Sp14	142	14
406	437	Galβ1-6Galβ-Sp10	141	38
407	144	Galβ1-3GalNAcβ1-4(Neu5Acα2-3)Galβ1-4Glcβ-Sp0	137	69
408	504	Galβ1-4GlcNAcβ1-6(Galβ1-4GlcNAcβ1-2)Manα1-6(GlcNAcβ1-4)Galβ1-4GlcNAcβ1-4(Gal β1-4GlcNAcβ1-2)Manα1-3)Manβ1-4GlcNAcβ1-4(Fucα1-6)GlcNAc-Sp21	135	22
409	587	Neu5Acα2-3Galβ1-4GlcNAcβ1-3Galβ1-4GlcNAcβ1-6(Neu5Acα2-3Galβ1-4GlcNAcβ1-3Galβ1-4GlcNAcβ1-3)GalNAcα-Sp14	133	27
410	591	Neu5Acα2-3Galβ1-4GlcNAcβ1-3Galβ1-4GlcNAcβ1-6(Galβ1-3)GalNAcα-Sp14	133	36
411	163	Galβ1-4GlcNAcβ1-3Galβ1-4GlcNAcβ-Sp0	131	45
412	235	Neu5Acα2-3GalNAcα-Sp8	129	66
413	364	Neu5Acα2-6GlcNAcβ1-4GlcNAcβ1-4GlcNAc-Sp21	129	39
414	2	Glcα-Sp8	125	35
415	149	Galβ1-3GlcNAcβ-Sp0	125	34
416	42	(6S)Galβ1-4Glcβ-Sp0	124	57
417	155	Galβ1-4(6S)Glcβ-Sp0	124	16
418	500	(3S)GalNAcβ1-4(Fucα1-3)GlcNAcβ-Sp8	124	59
419	98	GalNAcβ1-4GlcNAcβ-Sp0	123	17
420	477	Neu5Acα2-6Galβ1-4 GlcNAcβ1-6(Neu5Acα2-6Galβ1-4GlcNAcβ1-3)GalNAcα-Sp14	123	31
421	166	Galβ1-4GlcNAcβ1-6(Galβ1-3)GalNAcα-Sp8	122	7
422	233	Neu5Acα2-3Galβ1-3GalNAcβ1-4(Neu5Acα2-3)Galβ1-4Glcβ-Sp0	122	20
423	499	GalNAcβ1-4(Fucα1-3)(6S)GlcNAcβ-Sp8	122	44
424	9	Neu5Acα-Sp8	121	35
425	15	GalNAcβ-Sp8	121	59
426	30	(3S)Galβ1-3GlcNAcβ-Sp0	121	59
427	273	Neu5Acα2-8Neu5Acα-Sp8	121	26
428	262	Neu5Acα2-3Galβ1-4Glcβ-Sp8	120	41
429	384	Galβ1-4GlcNAcβ1-6(Galβ1-4GlcNAcβ1-2)Manα1-6(Galβ1-4GlcNAcβ1-4(Galβ1-4GlcNAcβ1-2)Manα1-3)Manβ1-4GlcNAcβ1-4GlcNAcβ-Sp21	120	57
430	249	Neu5Acα2-3Galβ1-4(6S)GlcNAcβ-Sp8	119	43

431	23	6S(3S)Galβ1-4GlcNAcβ-Sp0	118	34
432	529	Fucα1-4(Galβ1-3)GlcNAcβ1-2 Manα-Sp0	117	18
433	550	Galβ1-4GlcNAcβ1-3Galβ1-4GlcNAcβ1-6(Galβ1-4GlcNAcβ1-3Galβ1-4GlcNAcβ1-2)Manα1-6(Galβ1-4GlcNAcβ1-3Galβ1-4GlcNAcβ1-2Manα1-3)Manβ1-4GlcNAcβ1-4GlcNAc-Sp24	117	30
434	370	GalNAcα1-3(Fucα1-2)Galβ1-3GlcNAcβ1-2Manα1-6(GalNAcα1-3(Fucα1-2)Galβ1-3GlcNAcβ1-2Manα1-3)Manβ1-4GlcNAcβ1-4GlcNAcβ-Sp20	116	21
435	588	Neu5Acα2-6Galβ1-4GlcNAcβ1-3Galβ1-4GlcNAcβ1-3GalNAcα-Sp14	115	20
436	218	Neu5Acα2-3Galβ1-4GlcNAcβ1-3Galβ1-4(Fucα1-3)GlcNAcβ-Sp0	114	30
437	236	Neu5Acα2-3GalNAcβ1-4GlcNAcβ-Sp0	114	49
438	147	Galβ1-3GlcNAcβ1-3Galβ1-4GlcNAcβ-Sp0	113	7
439	379	Galβ1-3GlcNAcβ1-3Galβ1-4GlcNAcβ1-6(Galβ1-3GlcNAcβ1-3)Galβ1-4Glcβ-Sp21	110	42
440	532	GalNAcα1-3(Fucα1-2)Galβ1-3GalNAcβ1-3Galα1-4Galβ1-4Glc-Sp21	110	17
441	590	Galβ1-4GlcNAcβ1-3Galβ1-3GalNAcα-Sp14	110	31
442	561	(3S)GlcAβ1-3Galβ1-4GlcNAcβ1-2Manα-Sp0	109	38
443	269	Neu5Acα2-6Galβ1-4GlcNAcβ1-3Galβ1-4GlcNAcβ-Sp0	108	37
444	493	Fucα1-2(6S)Galβ1-3GlcNAcβ-Sp0	108	9
445	494	Galα1-3(Fucα1-2)Galβ1-4GlcNAcβ1-6GalNAcα-Sp14	107	23
446	134	GlcNAcβ1-6(Galβ1-3)GalNAcα-Sp14	106	13
447	439	GalNAcβ1-6GalNAcβ-Sp8	106	28
448	254	Neu5Acα2-3Galβ1-4(Fucα1-3)GlcNAcβ1-3Galβ-Sp8	105	24
449	256	Neu5Acα2-3Galβ1-4GlcNAcβ1-3Galβ1-4GlcNAcβ1-3Galβ1-4GlcNAcβ-Sp0	105	9
450	512	(4S)GalNAcβ-Sp10	103	34
451	27	(3S)Galβ1-4(6S)Glcβ-Sp8	102	28
452	283	Neu5Gcα2-6GalNAcα-Sp0	102	9
453	403	Galβ1-3GlcNAcβ1-6Galβ1-4GlcNAcβ-Sp0	102	34
454	49	Neu5,9Ac2α2-6Galβ1-4GlcNAcβ-Sp8	101	32
455	124	Galα1-6Glcβ-Sp8	101	15
456	279	Neu5Gcα2-3Galβ1-3GlcNAcβ-Sp0	101	22
457	482	Neu5Acα2-3Galβ1-3GlcNAcβ1-2Manα1-6(GlcNAcβ1-4)(Neu5Acα2-3Galβ1-3GlcNAcβ1-2Manα1-3)Manβ1-4GlcNAcβ1-4GlcNAc-Sp21	100	7
458	409	Neu5Acα2-3Galβ1-3GalNAcβ1-4(Neu5Acα2-8Neu5Acα2-3)Galβ1-4Glcβ-Sp0	99	10
459	410	Galα1-3(Fucα1-2)Galβ1-4GlcNAcβ1-3GalNAcα-Sp14	99	12
460	398	Galβ1-4(Fucα1-3)GlcNAcβ1-3GalNAcα-Sp14	98	12
461	404	Galβ1-3GlcNAcα1-6Galβ1-4GlcNAcβ-Sp0	98	49
462	64	Fucα1-2Galβ1-3GalNAcβ1-4(Neu5Acα2-3)Galβ1-4Glcβ-Sp9	96	21
463	125	Galβ1-2Galβ-Sp8	96	11
464	305	GlcAβ1-3GlcNAcβ-Sp8	96	97
465	329	Neu5Acα2-6Galβ1-4GlcNAcβ1-3Galβ1-4GlcNAcβ1-3Galβ1-4GlcNAcβ-Sp0	96	21
466	92	GalNAcα1-3Galβ-Sp8	95	25
467	242	Neu5Acα2-6(Neu5Acα2-3Galβ1-3)GalNAcα-Sp8	94	16
468	432	Galβ1-4GlcNAcβ1-2Manα1-6(GlcNAcβ1-4)(Galβ1-4GlcNAcβ1-2Manα1-3)Manβ1-4GlcNAcβ1-4GlcNAc-Sp21	94	22
469	450	GalNAcβ1-4Galβ1-4Glcβ-Sp0	94	21
470	492	Galβ1-4(Fucα1-3)GlcNAcβ1-2Manα-Sp0	94	7
471	115	Galα1-3Galβ1-4GlcNAcβ-Sp8	92	31
472	295	(6S)Galβ1-4(6S)GlcNAcβ-Sp0	92	30
473	495	Fucα1-2Galβ1-4GlcNAcβ1-2Manα-Sp0	92	19
474	568	Galβ1-4GlcNAcβ1-3Galβ1-4GlcNAcβ1-3Galβ1-4GlcNAcβ1-2Manα1-6(Galβ1-4GlcNAcβ1-3Galβ1-4GlcNAcβ1-3Galβ1-4GlcNAcβ1-2Manα1-3)Manβ1-4GlcNAcβ1-4(Fucα1-6)GlcNAcβ-Sp24	92	83
475	229	Neu5Acα2-8Neu5Acα2-8Neu5Acα-Sp8	91	40
476	318	Neu5Acα2-8Neu5Acβ-Sp17	91	15
477	407	Galβ1-4GlcNAcβ1-6(Neu5Acα2-6Galβ1-3GlcNAcβ1-3)Galβ1-4Glc-Sp21	90	8
478	412	GalNAcα1-3GalNAcβ1-3Galα1-4Galβ1-4Glcβ-Sp0	90	96
479	564	Neu5Acα2-8Neu5Acα2-3Galβ1-3GalNAcβ1-4(Neu5Acα2-3)Galβ1-4Glc-Sp21	89	4
480	172	Galβ1-4Glcβ-Sp8	88	27
481	252	Neu5Acα2-3Galβ1-4(Fucα1-3)GlcNAcβ-Sp0	88	34
482	463	Neu5Acα2-6Galβ1-4GlcNAcβ1-6(Neu5Acα2-6Galβ1-4GlcNAcβ1-2)Manα1-6(GlcNAcβ1-4)(Neu5Acα2-6Galβ1-4GlcNAcβ1-4(Neu5Acα2-6Galβ1-4GlcNAcβ1-2)Manα1-3)Manβ1-4GlcNAcβ1-4GlcNAcβ-Sp21	88	9
483	25	(3S)Galβ1-4Glcβ-Sp8	87	30
484	414	Galα1-3(Fucα1-2)Galβ1-4(Fucα1-3)GlcNAcβ1-3GalNAc-Sp14	87	21
485	456	Neu5Acα2-3Galβ1-4GlcNAcβ1-2Manα1-6(GlcNAcβ1-4)(Neu5Acα2-3Galβ1-4GlcNAcβ1-2Manα1-3)Manβ1-4GlcNAcβ1-4GlcNAcβ-Sp21	87	13
486	558	Galβ1-3GlcNAcβ1-6(Galβ1-3)GalNAc-Sp14	87	15
487	45	(6S)Galβ1-4(6S)Glcβ-Sp8	86	10
488	408	Galβ1-3GalNAcβ1-4(Neu5Acα2-8Neu5Acα2-3)Galβ1-4Glcβ-Sp0	86	15
489	433	Galβ1-4GlcNAcβ1-2Manα1-6(GlcNAcβ1-4)(Galβ1-4GlcNAcβ1-4(Galβ1-4GlcNAcβ1-2)Manα1-3)Manβ1-4GlcNAcβ1-4GlcNAc-Sp21	86	19

490	583	Neu5Acα2-3Galβ1-4GlcNAcβ1-3Galβ1-4GlcNAcβ1-3GalNAcα-Sp14	86	13
491	232	GalNAcβ1-4(Neu5Acα2-3)Galβ1-4Glcβ-Sp0	85	25
492	517	Galα1-3Galβ1-4GlcNAcβ1-2Manα-Sp0	85	12
493	40	(4S)Galβ1-4GlcNAcβ-Sp8	84	6
494	246	Neu5Acα2-3Galβ1-3GlcNAcβ1-3Galβ1-4GlcNAcβ-Sp0	83	21
495	399	GalNAcα1-3GalNAcβ1-3Galα1-4Galβ1-4GlcNAcβ-Sp0	83	44
496	121	Galα1-4Galβ1-4GlcNAcβ-Sp8	82	46
497	253	Neu5Acα2-3Galβ1-4(Fucα1-3)GlcNAcβ-Sp8	82	10
498	162	Galβ1-4GlcNAcβ1-3Galβ1-4GlcNAcβ1-3Galβ1-4GlcNAcβ-Sp0	81	22
499	326	Neu5,9Ac2α2-3Galβ1-3GlcNAcβ-Sp0	81	18
500	390	GalNAcα1-3(Fucα1-2)Galβ1-3GalNAcα1-3(Fucα1-2)Galβ1-4GlcNAcβ-Sp0	81	31
501	438	Neu5Acα2-3Galβ1-4GlcNAcβ1-3Galβ-Sp8	81	27
502	525	Neu5Acα2-3Galβ1-3GlcNAcβ1-4Galβ1-4Glcβ-Sp0	80	3
503	48	Neu5,9Ac2α-Sp8	79	61
504	244	Neu5Acα2-3Galβ-Sp8	79	5
505	378	Galβ1-3GalNAcα1-3(Fucα1-2)Galβ1-4GlcNAc-Sp0	79	13
506	539	GlcNAcβ1-3Galβ1-4GlcNAcβ1-3Galβ1-4GlcNAcβ1-2Manα1-6(GlcNAcβ1-3Galβ1-4GlcNAcβ1-3Galβ1-4GlcNAcβ1-2Manα1-3)Manβ1-4GlcNAcβ1-4GlcNAcβ-Sp25	79	12
507	541	Galβ1-4GlcNAcβ1-3Galβ1-4GlcNAcβ1-3Galβ1-4GlcNAcβ1-2Manα1-6(Galβ1-4GlcNAcβ1-3Galβ1-4GlcNAcβ1-3Galβ1-4GlcNAcβ1-2Manα1-3)Manβ1-4GlcNAcβ1-4GlcNAcβ-Sp24	79	15
508	165	Galβ1-4GlcNAcβ1-3Galβ1-4Glcβ-Sp8	78	117
509	475	Neu5Acα2-3Galβ1-4GlcNAcβ1-6GalNAcα-Sp14	78	15
510	228	GalNAcβ1-4(Neu5Acα2-8Neu5Acα2-3)Galβ1-4Glcβ-Sp0	77	51
511	374	Neu5Acα2-6Galβ1-4GlcNAcβ1-3GalNAc-Sp14	77	5
512	405	GalNAcβ1-3Galα1-6Galβ1-4Glcβ-Sp8	77	7
513	421	GalNAcα1-3(Fucα1-2)Galβ1-3GlcNAcβ1-3GalNAc-Sp14	77	13
514	434	Galβ1-4GlcNAcβ1-6(Galβ1-4GlcNAcβ1-2)Manα1-6(GlcNAcβ1-4)(Galβ1-4GlcNAcβ1-2Manα1-3)Manβ1-4GlcNAcβ1-4GlcNAc-Sp21	77	6
515	445	Galβ1-4GlcNAcβ1-2Manα-Sp0	77	16
516	210	Manα1-6(Manα1-2Manα1-3)Manα1-6(Manα1-2Manα1-3)Manβ1-4GlcNAcβ1-4GlcNAcβ-Sp12	76	5
517	231	GalNAcβ1-4(Neu5Acα2-3)Galβ1-4GlcNAcβ-Sp8	76	13
518	420	Galα1-3(Fucα1-2)Galβ1-3GlcNAcβ1-3GalNAc-Sp14	76	15
519	422	Galα1-3Galβ1-3GlcNAcβ1-3GalNAc-Sp14	76	8
520	508	(6S)GalNAcβ1-4GlcNAc-Sp8	76	44
521	6	Fucα-Sp8	75	16
522	575	Galβ1-4GlcNAcβ1-3Galβ1-4GlcNAcβ1-3Galβ1-4GlcNAcβ1-6(Galβ1-4GlcNAcβ1-3Galβ1-4GlcNAcβ1-3Galβ1-4GlcNAβ1-2)Manα1-6(Galβ1-4GlcNAcβ1-3Galβ1-4GlcNAcβ1-3Galβ1-4GlcNAcβ1-2Manα1-3)Manβ1-4GlcNAcβ1-4(Fucα1-6)GlcNAcβ-Sp24	75	13
523	116	Galα1-3Galβ1-4Glcβ-Sp0	74	19
524	402	Galα1-3Galβ1-4GlcNAcβ1-3GalNAcα-Sp14	74	11
525	39	(6S)(4S)Galβ1-4GlcNAcβ-Sp0	73	13
526	135	Neu5Acα2-6(Galβ1-3)GalNAcα-Sp8	73	30
527	553	GalNAcβ1-3GlcNAcβ-Sp0	73	38
528	449	Neu5Acα2-8Neu5Acα2-3Galβ1-3GalNAcβ1-4(Neu5Acα2-8Neu5Acα2-3)Galβ1-4Glcβ-Sp0	72	44
529	123	Galα1-4GlcNAcβ-Sp8	71	11
530	169	Galβ1-4GlcNAcβ-Sp8	71	5
531	171	Galβ1-4Glcβ-Sp0	71	7
532	335	GlcNAcα1-4Galβ1-4GlcNAcβ1-3Galβ1-4GlcNAcβ1-3Galβ1-4GlcNAcβ-Sp0	70	4
533	546	Neu5Gcα2-8Neu5Gcα2-3Galβ1-4GlcNAcβ1-3Galβ1-4GlcNAc-Sp0	70	29
534	264	Neu5Acα2-6GalNAcβ1-4GlcNAcβ-Sp0	69	17
535	436	Galβ1-4Galβ-Sp10	69	12
536	506	Galβ1-3(6S)GlcNAcβ-Sp8	68	18
537	543	Neu5Gcα2-8Neu5Gcα2-3Galβ1-4GlcNAc-Sp0	68	8
538	240	Neu5Acα2-3Galβ1-4(Neu5Acα2-3Galβ1-3)GlcNAcβ-Sp8	67	72
539	302	Galβ1-4GlcNAcα1-6Galβ1-4GlcNAcβ-Sp0	67	14
540	485	Galα1-3Galβ1-3GlcNAcβ1-6GalNAcα-Sp14	66	58
541	373	Neu5Acα2-3Galβ1-4GlcNAcβ1-3GalNAc-Sp14	64	10
542	491	Galα1-3Galβ1-4GlcNAcβ1-6GalNAcα-Sp14	64	6
543	10	Neu5Acα-Sp11	63	12
544	157	Galβ1-4GalNAcα1-3(Fucα1-2)Galβ1-4GlcNAcβ-Sp8	63	3
545	462	Neu5Acα2-6Galβ1-4GlcNAcβ1-6(Neu5Acα2-6Galβ1-4GlcNAcβ1-2)Manα1-6(GlcNAcβ1-4)(Neu5Acα2-6Galβ1-4GlcNAcβ1-2Manα1-3)Manβ1-4GlcNAcβ1-4GlcNAcβ-Sp21	63	12
546	498	Neu5Acα2-6GalNAcβ1-4(6S)GlcNAcβ-Sp8	63	12
547	502	GalNAcα1-3(Fucα1-2)Galβ1-3GlcNAcβ1-6GalNAcα-Sp14	63	12
548	516	Neu5Acα2-6Galβ1-4GlcNAcβ1-2Man-Sp0	63	14
549	29	(3S)Galβ1-3GalNAcα-Sp8	62	11
550	234	Neu5Acα2-6(Neu5Acα2-3)GalNAcα-Sp8	62	16

551	560	(3S)GlcAβ1-3Galβ1-4GlcNAcβ1-3Galβ1-4Glc-Sp0	62	37
552	146	Galβ1-3Galβ-Sp8	61	12
553	457	Neu5Acα2-3Galβ1-4GlcNAcβ1-4Manα1-6(GlcNAcβ1-4)(Neu5Acα2-3Galβ1-4GlcNAcβ1-4(Neu5Acα2-3Galβ1-4GlcNAcβ1-2)Manα1-3)Manβ1-4GlcNAcβ1-4GlcNAcβ-Sp21	61	9
554	393	Neu5Acα2-3Galβ1-3GlcNAcβ1-2Manα1-6(Neu5Acα2-3Galβ1-3GlcNAcβ1-2Manα1-3)Manβ1-4GlcNAcβ1-4GlcNAc-Sp19	59	11
555	460	Neu5Acα2-6Galβ1-4GlcNAcβ1-2Manα1-6(GlcNAcβ1-4)(Neu5Acα2-6Galβ1-4GlcNAcβ1-2Manα1-3)Manβ1-4GlcNAcβ1-4GlcNAcβ-Sp21	59	13
556	241	Neu5Acα2-3Galβ1-3(6S)GalNAcα-Sp8	58	12
557	435	Galβ1-4GlcNAcβ1-6(Galβ1-4GlcNAcβ1-2)Manα1-6(GlcNAcβ1-4)(Galβ1-4GlcNAcβ1-4(Galβ1-4GlcNAcβ1-2)Manα1-3)Manβ1-4GlcNAcβ1-4GlcNAc-Sp21	58	31
558	276	Neu5Acβ2-6GalNAcα-Sp8	57	6
559	521	Galα1-3(Fucα1-2)Galβ1-3GlcNAcβ1-6GalNAc-Sp14	57	21
560	26	(3S)Galβ1-4(6S)Glcβ-Sp0	56	23
561	138	Neu5Acα2-6(Galβ1-3)GlcNAcβ1-4Galβ1-4Glcβ-Sp10	56	15
562	170	Galβ1-4GlcNAcβ-Sp23	56	15
563	159	Galβ1-4GlcNAcβ1-3GalNAcα-Sp8	55	8
564	263	Neu5Acα2-6GalNAcα-Sp8	55	7
565	461	Neu5Acα2-6Galβ1-4GlcNAcβ1-4Manα1-6(GlcNAcβ1-4)(Neu5Acα2-6Galβ1-4GlcNAcβ1-4(Neu5Acα2-6Galβ1-4GlcNAcβ1-2)Manα1-3)Manβ1-4GlcNAcβ1-4GlcNAcβ-Sp21	55	4
566	277	Neu5Acβ2-6Galβ1-4GlcNAcβ-Sp8	53	16
567	547	Neu5Gcα2-8Neu5Gcα2-6Galβ1-4GlcNAc-Sp0	53	8
568	574	GlcNAcβ1-3Galβ1-4GlcNAcβ1-3Galβ1-4GlcNAcβ1-6(GlcNAcβ1-3Galβ1-4GlcNAcβ1-3Galβ1-4GlcNAβ1-2)Manα1-6(GlcNAcβ1-3Galβ1-4GlcNAcβ1-3Galβ1-4GlcNAcβ1-2Manα1-3)Manβ1-4GlcNAcβ1-4(Fucα1-6)GlcNAcβ-Sp24	53	39
569	148	Galβ1-3GlcNAcβ1-3Galβ1-4Glcβ-Sp10	52	7
570	267	Neu5Acα2-6Galβ1-4GlcNAcβ-Sp8	52	5
571	265	Neu5Acα2-6Galβ1-4(6S)GlcNAcβ-Sp8	51	4
572	505	Galβ1-3GlcNAcα1-3Galβ1-4GlcNAcβ-Sp8	50	13
573	548	Neu5Acα2-8Neu5Acα2-3Galβ1-4GlcNAc-Sp0	50	20
574	31	(3S)Galβ1-3GlcNAcβ-Sp8	49	9
575	137	Neu5Acβ2-6(Galβ1-3)GalNAcα-Sp8	49	9
576	139	Galβ1-3GalNAcα-Sp8	49	18
577	38	(3S)Galβ-Sp8	48	8
578	514	(6P)Galβ1-4GlcNAcβ-SP0	46	7
579	458	Neu5Acα2-3Galβ1-4GlcNAcβ1-6(Neu5Acα2-3Galβ1-4GlcNAcβ1-2)Manα1-6(GlcNAcβ1-4)(Neu5Acα2-3Galβ1-4GlcNAcβ1-2Manα1-3)Manβ1-4GlcNAcβ1-4GlcNAcβ-Sp21	45	3
580	459	Neu5Acα2-3Galβ1-4GlcNAcβ1-6(Neu5Acα2-3Galβ1-4GlcNAcβ1-2)Manα1-6(GlcNAcβ1-4)(Neu5Acα2-3Galβ1-4GlcNAcβ1-4(Neu5Acα2-3Galβ1-4GlcNAcβ1-2)Manα1-3)Manβ1-4GlcNAcβ1-4GlcNAcβ-Sp21	41	16
581	545	Neu5Gcα2-8Neu5Acα2-3Galβ1-4GlcNAc-Sp0	41	5
582	544	Neu5Acα2-8Neu5Gcα2-3Galβ1-4GlcNAc-Sp0	40	8
583	440	(6S)Galβ1-3GlcNAcβ-Sp0	39	34
584	507	(6S)(4S)GalNAcβ1-4GlcNAc-Sp8	37	8
585	416	Galβ1-4(Fucα1-3)GlcNAcβ1-2Manα1-6(Galβ1-4(Fucα1-3)GlcNAcβ1-2Manα1-3)Manβ1-4GlcNAcβ1-4(Fucα1-6)GlcNAcβ-Sp22	33	35
586	484	Galβ1-3GlcNAcβ1-6GalNAcα-Sp14	33	19
587	509	(3S)GalNAcβ1-4(3S)GlcNAc-Sp8	30	13
588	559	Galβ1-4GlcNAcβ1-3Galβ1-4GlcNAcβ1-3Galβ1-4GlcNAcβ1-3Galβ1-4GlcNAcβ1-3Galβ1-4GlcNAcβ1-3Galβ1-4GlcNAcβ1-2Manα1-6(Galβ1-4GlcNAcβ1-3Galβ1-4GlcNAcβ1-3Galβ1-4GlcNAcβ1-3Galβ1-4GlcNAcβ1-3Galβ1-4GlcNAcβ1-3Galβ1-4GlcNAcβ1-2Manα1-3)Manβ1-4GlcNAcβ1-4GlcNAcβ-Sp25	28	16
589	377	Galβ1-3GalNAcα1-3(Fucα1-2)Galβ1-4Glc-Sp0	26	4
590	340	GlcNAcα1-4Galβ1-4GlcNAcβ1-3Galβ1-4GlcNAcβ-Sp0	21	46
591	142	Galβ1-3GalNAcβ-Sp8	19	23
592	224	Neu5Acα2-3Galβ1-3GalNAcα-Sp14	19	53
593	441	(6S)Galβ1-3(6S)GlcNAc-Sp0	16	14
594	388	Galβ1-3GlcNAcβ1-3GalNAcα-Sp14	15	22
595	201	GlcAα-Sp8	-5	72
596	274	Neu5Acα2-8Neu5Acα2-3Galβ1-4Glcβ-Sp0	-16	11
597	565	GlcNAcβ1-3Galβ1-4GlcNAcβ1-2Manα1-6(GlcNAcβ1-3Galβ1-4GlcNAcβ1-2Manα1-3)Manβ1-4GlcNAcβ1-4(Fucα1-6)GlcNAcβ-Sp24	-37	51
598	259	Neu5Acα2-3Galβ1-4GlcNAcβ1-3Galβ1-4GlcNAcβ-Sp0	-38	20
599	211	Manα1-2Manα1-6(Manα1-3)Manα1-6(Manα1-2Manα1-2Manα1-3)Manβ1-4GlcNAcβ1-4GlcNAcβ-Sp12	-58	25
600	327	Neu5Acα2-6Galβ1-4GlcNAcβ1-3Galβ1-3GlcNAcβ-Sp0	-131	30
601	363	Neu5Acα2-6GlcNAcβ1-4GlcNAc-Sp21	-149	77

Table S3. Crystallization conditions

Abbreviation in the text: Carbohydrate/crystal form	**αMeMan/1**	**αMeMan/2**	**αMeMan/3**	**Manα1-2Man/2**	**Manα1-2Man/4**	**Manα1-6Man/2**	**Manα1-6Man/5**
Protein solution	6 mg/ml protein in 5 mM $CaCl_2$, 10 mM Tris, pH 8.0, 25 mM NaCl, and 50 mM α-methyl mannose	6 mg/ml protein in 5 mM $CaCl_2$, 10 mM Tris, pH 8.0, 25 mM NaCl, and 50 mM α-methyl mannose	7.5 mg/ml protein in 5 mM $CaCl_2$, 10 mM Tris, pH 8.0, 25 mM NaCl, and 50 mM α-methyl mannose	5 mg/ml protein in 5 mM $CaCl_2$, 10 mM Tris, pH 8.0, 25 mM NaCl, and 30 mM Manα1-2Man	6 mg/ml protein in 5 mM $CaCl_2$, 10 mM Tris, pH 8.0, 25 mM NaCl, and 30 mM Manα1-2Man	6 mg/ml protein in 5 mM $CaCl_2$, 10 mM Tris, pH 8.0, 25 mM NaCl, and 30 mM Manα1-6Man	6 mg/ml protein in 5 mM $CaCl_2$, 10 mM Tris, pH 8.0, 25 mM NaCl, and 30 mM Manα1-6Man
Reservoir solution	2.5% Peg 3.35K or 10% Peg 400, 0.1 M MES, pH 6.0	10% Peg 400, 0.1 M MES, pH 6.0	15% Peg 8K, 0.1M MES, pH 6.0	15% Peg 400, 0.1 M MES, pH 6.0	15% Peg 400, 0.1 M MES, pH 6.0	5% Peg 8K, 0.1 M MES, pH 6.0	15% Peg 400, 0.1 M MES, pH 6.0
Hanging drop protein: reservoir ratio (µl)	0.9:0.9 or 1.8:0.9	0.9:1.8	0.9:0.9	1.8:0.9	0.9:0.9	0.9:0.9	0.9:0.9
Freezing solution	25% Peg 3.5K or 400, 5 mM $CaCl_2$, 25 mM NaCl, 0.1 M HEPES, pH 7.0, 50 mM α-methyl mannose	25% Peg 400, 5 mM $CaCl_2$, 25 mM NaCl, 0.1 M MES, pH 6.0, 50 mM α-methyl mannose	25% Peg 8K, 5 mM $CaCl_2$, 25 mM NaCl, 0.1 M MES, pH 6.5, 50 mM α-methyl mannose	25% Peg 400, 5 mM $CaCl_2$, 25 mM NaCl, 0.1 M MES, pH 6.0, 30 mM Manα1-2Man	25% Peg 400, 5 mM $CaCl_2$, 25 mM NaCl, 0.1 M MES, pH 6.0, 30 mM Manα1-2Man	25% Peg 8K, 5 mM $CaCl_2$, 25 mM NaCl, 0.1 M MES, pH 6.5, 30 mM Manα1-6Man	25% Peg 400, 5 mM $CaCl_2$, 25mM NaCl, 0.1 M MES, pH 6.5, 30 mM Manα1-6Man

Abbreviation in the text: Carbohydrate/crystal form	**Fucα1-2Galß1-4Glc/3**	**Fucα1-2Galß1-4Glc/2**	**Fucα1-2Galß1-4Glc/6**	**Lewis-a/3**	**αMeGlcNAc/3**	**αMeGlcNAc/7**	**Fucα1-3GlcNAc/2**	**Native/8**
Protein solution	6 mg/ml protein in 5 mM $CaCl_2$, 10 mM Tris, pH 8.0, 25 mM NaCl, and 15 mM Fucα1-2Galß1-4Glc	6 mg/ml protein in 5 mM $CaCl_2$, 10 mM Tris, pH 8.0, 25 mM NaCl, and 15 mM Fucα1-2Galß1-4Glc	6 mg/ml protein in 5 mM $CaCl_2$, 10 mM Tris, pH 8.0, 25 mM NaCl, and 15 mM Fucα1-2Galß1-4Glc	6 mg/ml protein in 5 mM $CaCl_2$, 10 mM Tris, pH 8.0, 25 mM NaCl, and 20 mM Lewis[a]	6 mg/ml protein in 5 mM $CaCl_2$, 10 mM Tris, pH 8.0, 25 mM NaCl, and 50 mM αMeGlcNAc	3 mg/ml protein in 5 mM $CaCl_2$, 10 mM Tris, pH 8.0, 25 mM NaCl, and 50 mM αMeGlcNAc	6 mg/ml protein in 5 mM $CaCl_2$, 10 mM Tris, pH 8.0, 25 mM NaCl, and 15 mM Fucα1-3GlcNAc	6 mg/ml protein in 5 mM $CaCl_2$, 10 mM Tris, pH 8.0, 25 mM NaCl, and 10 mM $Man_9GlcNAc_2$
Reservoir solution	12.5% Peg 8K, 0.1 M MES, pH 6.0	12.5% Peg 8K, 0.1 M MES, pH 6.0	12.5% Peg 8K, 0.1 M MES, pH 6.0	20% Peg 8K, 0.1 M MES, pH 6.0	15% Peg 8K, 0.1 M MES, pH 6.0	20% Peg 8K, 0.1 M MES, pH 6.0	12.5% Peg 8K, 0.1 M MES, pH 6.0	2.5% Peg 8K, 0.1 M MES, pH 6.0
Hanging drop protein: reservoir ratio (µl)	0.9:1.8	0.9:1.8	0.9:1.8	0.9:1.8	0.9: 0.9	0.9: 0.9	0.9:1.8	0.9: 0.9
Freezing solution	25% Peg 8K, 5 mM $CaCl_2$, 25 mM NaCl, 0.1 M MES, pH 6.0, 1 5 mM Fucα1-2Galß1-4Glc	25% Peg 8K, 5 mM $CaCl_2$, 25 mM NaCl, 0.1 M MES, pH 6.0, 15 mM Fucα1-2Galß1-4Glc	25% Peg 8K, 5 mM $CaCl_2$, 25 mM NaCl, 0.1 M MES, pH 6.5, 15 mM Fucα1-2Galß14-Glc	25% Peg 8K, 5 mM $CaCl_2$, 25 mM NaCl, 0.1 M MES, pH 6.0, 20 mM Lewis[a]	25% Peg 8K, 5 mM $CaCl_2$, 25 mM NaCl, 0.1 M MES, pH 6.0, 50 mM αMeGlcNAc	25% Peg 8K, 5 mM $CaCl_2$, 25 mM NaCl, 0.1M MES, pH 6.0, 50 mM αMeGlcNAc	25% Peg 8K, 5 mM $CaCl_2$, 25 mM NaCl, 0.1 M MES, pH 6.5, 15 mM Fuc α1-3GlcNAc	25% Peg 8K, 5 mM $CaCl_2$, 25 mM NaCl, 0.1 M MES, pH 6.0, 10 mM $Man_9GlcNAc_2$

Peg, polyethylene glycol

Table S4. Crystallographic data statistics

Abbreviation in the text: Carbohydrate/crystal form	**αMeMan/1**	**αMeMan/2**	**αMeMan/3**	**Manα1-2Man/2**	**Manα1-2Man/4**	**Manα1-6Man/2**	**Manα1-6Man/5**
Symmetry	P1	$P2_1$	$P2_1$	$P2_1$	$P2_1$	$P2_1$	$P2_1$
Number of monomers in AU transitional-NCS	2<br>t-NCS 0 0.5 0.5	1	1	1	2<br>t-NCS 0.5 0 0.5	1	1
Wavelength (Å)	0.97946	0.97946	0.97946	0.97946	0.97946	0.97946	0.97946
Unit cell lengths (Å)	a=30.14 b=37.84 c=55.07 α=94.08 β=99.63 γ=104.98	a=34.27 b=54.01 c=35.53 β= 95.86	a=32.97 b=56.36 c=34.22 β=112.01	a=34.23 b=54.25 c=35.71 β=95.37	a= 34.41 b= 56.38 c= 61.37 β= 100.92	a=34.19 b=54.25 c=35.94 β=95.01	a=31.92 b=59.04 c=34.85 β=114.49
Resolution Å (last shell)	1.40 (1.42)	1.40 (1.42)	1.20 (1.22)	1.40 (1.42)	1.40 (1.42)	1.40 (1.42)	1.40 (1.42)
R_{sym} (last shell)[a]	10.9 (23.4)	4.7 (8.2)	7.5 (7.8)	4.0(14.3)	4.2 (12.3)	4.0 (8.5)	4.6 (24.2)
Mn(I) half-set correlation CC(1/2) (last shell)	0.997 (0.983)	0.999 (0.995)	0.995 (0.993)	0.999 (0.990)	0.998 (0.981)	0.999 (0.995)	0.999 (0.979)
Mean((I)/s(I)) (last shell)	9.0 (3.6)	22.0 (10.3)	20.9 (15.2)	25.4 (8.3)	18.0 (7.9)	28.1 (13.6)	16.9 (3.4)
% completeness (last shell)	97.2 (85.2)	98.0 (94.6)	98.1 (95.9)	96.6 (93.6)	98.0 (95.8)	98.2 (95.7)	98.0 (96.5)
Number of unique reflections	44495	25015	35621	24845	44557	25400	22757
Average multiplicity (last shell)	6.9 (6.2)	6.9 (6.5)	6.7 (6.2)	7.0 (6.7)	3.6 (3.5)	6.8 (6.4)	6.8 (6.4)

[a]Rsym = $100x\sum_h\sum_I (| I_i(h) - \langle I(h)\rangle |) / \sum_h\sum_i I_i(h)$ where Ii(h) = observed intensity, and <I(h)> = mean intensity obtained from multiple measurements.

AU, asymmetric unit; NCS, non-crystallographic symmetry.

Abbreviation in the text: Carbohydrate/crystal form	**Fucα1-2Galß1-4Glc/3**	**Fucα1-2Galß1-4Glc/2**	**Fucα1-2Galß1-4Glc/6**	**Lewis-a/3**	**αMeGlcNAc/3**	**αMeGlcNAc/7**	**Fucα1-3GlcNAc/2**	**Native/8**
Symmetry	$P2_1$	$P2_1$	$P2_1$	$P2_1$	$P2_1$	$P2_12_12$	$P2_1$	$P2_1$
Number of monomers in AU	1	1	1	1	1	1	1	2
Wavelength (Å)	0.97946	0.97946	0.97946	0.97946	0.97946	0.97946	0.97946	0.97946
Unit cell lengths (Å)	a=33.06 b=56.53 c=34.54 β=112.94	a=34.08 b=54.06 c=35.88 β=95.00	a=34.51 b=54.48 c=37.17 β=101.31	a=33.05 b=57.18 c=34.38 β=112.81	a=32.81 b=56.33 c=34.33 β=112.08	a=54.48 b=66.63 c=34.44	a=34.12 b=54.24 c=35.81 β=94.99	a=31.62 b=58.72 c=62.38 β=97.66
Resolution Å (last shell)	1.35 (1.37)	1.55 (1.57)	1.65 (1.68)	1.35 (1.37)	1.35 (1.37)	1.75 (1.78)	1.20 (1.22)	1.40 (1.42)
R_{sym} (last shell)[a]	4.9 (8.7)	5.9 (12.8)	5.6 (30.3)	4.2 (14.7)	6.4 (11.6)	7.2 (38.4)	5.6 (15.1)	5.7 (11.1)
Mn(I) half-set correlation CC(1/2) (last shell)	0.995 (0.995)	0.998 (0.988)	0.999 (0.983)	0.997 (0.986)	0.997 (0.989)	0.998 (0.979)	0.997 (0.989)	0.997 (0.990)
Mean((I)/s(I)) (last shell)	28.8 (13.0)	17.5 (6.6)	17.9 (5.1)	26.8 (9.2)	18.8 (9.7)	20.2 (6.9)	20.2 (9.0)	23.6 (12.4)
% completeness (last shell)	98.9 (98.1)	98.9 (94.3)	98.4 (99.4)	99.0 (99.0)	99.0 (97.4)	99.7 (98.2)	99.0 (97.7)	94.4 (92.6)
Number of unique reflections	25483	18748	16058	25718	25247	13185	40273	42022
Average multiplicity (last shell)	6.9 (6.7)	6.9 (6.6)	7.1 (7.0)	6.9 (6.8)	6.9 (6.6)	13.2 (13.3)	6.8 (6.4)	7.4 (7.3)

[a]Rsym = $100x\sum_h\sum_I (| I_i(h) - \langle I(h)\rangle |) / \sum_h\sum_i I_i(h)$ where Ii(h) = observed intensity, and <I(h)> = mean intensity obtained from multiple measurements.

AU, asymmetric unit.

Table S5. Crystallographic refinement statistics

Abbreviation in the text: Carbohydrate/crystal form	**αMeMan/1**	**αMeMan/2**	**αMeMan/3**	**Manα1-2Man/2**	**Manα1-2Man/4**	**Manα1-6Man/2**	**Manα1-6Man/5**
Residues, ions and sugars included in model	A: 640-761<br>2 Ca^{2+} (remote at 0.5 occupancy)<br>α-methyl mannose<br>B: 627-647 651-761<br>1 Ca^{2+}<br>α-methyl mannose	627-635 638-761<br>(634-635 638-640 at occupancy 0.5 with alternate conformations)<br>1 Ca^{2+}<br>α-methyl mannose	640-761<br>1 Ca^{2+}<br>α-methyl mannose	627-761<br>1 Ca^{2+}<br>Manα1-2-Man	A: 637-761<br>2 Ca^{2+} (remote in multiple conformation)<br>Manα1-2-Man<br>B: 628-635 639-761<br>1 Ca^{2+}<br>Manα1-2-Man	627-761<br>1 Ca^{2+}<br>Mannose with alternate conformation<br>(0.75:0.25)<br>(second mannose not visible)	639-761<br>2 Ca^{2+} (remote at 0.75 occupancy)<br>Mannose with alternate conformation<br>(0.75:0.25)<br>(second mannose not visible)
Number of reflections used for refinement	42487	23748	33826	23608	42540	24111	21605
Reflections marked for R_{free}	2000	1247	1774	1230	2000	1272	1127
R_{free}[a]	18.0	16.0	15.7	16.7	17.0	16.8	18.6
R_{cryst}[a]	14.8	13.5	13.4	13.9	13.4	13.7	15.2
Average B factor (Å^2)	19.1	16.1	11.3	17.8	14.5	15.6	23.0
Bond length rmsd (Å)	0.004	0.005	0.005	0.005	0.005	0.005	0.005
Angle rmsd (˚)	0.831	0.862	0.937	0.862	0.889	0.836	0.798
Ramachandran plot:<br>(% in each region)[b] Preferred/ Allowed/ Outliers	92.9/5.9/1.2	93.0/7.0/0	92.7/7.3/0	93.0/7.0/0	94.2/5.8/0	93.1/6.9/0	93.1/6.9/0
PDB ID	7JUD	7JUC	7JUB	7JUE	7JUF	7JUG	7JUH

[a]R and Rfree = $100x\sum_h |F_o(h)-F_c(h)| / \sum_h F_o(h)$, where $F_o(h)$ = observed structure factor amplitude and $F_c(h)$ = calculated structure factor amplitude for the working and test sets, respectively.

[b]As defined in Coot.

Abbreviation in the text: Carbohydrate/crystal form	**Fucα1-2Galß1-4Glc/3**	**Fucα1-2Galß1-4Glc/2**	**Fucα1-2Galß1-4Glc/6**	**Lewis-a/3**	**αMeGlcNAc/3**	**αMeGlcNAc/7**	**Fucα1-3GlcNAc/2**	**Native/8**
Residues, ions and sugars included in model	640-761<br>1 Ca^{2+}<br>Fucα1-2Galß1-4Glc	627-761<br>1 Ca^{2+}<br>Fucα1-2Galß1-4Glc	627-761<br>1 Ca^{2+}<br>Fucα1-2Galß1-4Glc	640-761<br>2 Ca^{2+} (remote at 0.75 occupancy)<br>Lewis-a	640-761<br>1 Ca^{2+}<br>αMeGlcNAc	628-761<br>1 Ca^{2+}<br>αMeGlcNAc	627-761<br>1 Ca^{2+}<br>Fucα1-3GlcNAc	A 629-761<br>B 627-761<br>2 Ca^{2+}
Number of reflections used for refinement	24194	17795	15238	24413	23967	12498	38266	40004
Reflections marked for R_{free}	1269	936	813	1285	1260	658	1987	2000
R_{free} [a]	19.0	16.7	20.9	19.8	17.0	22.0	17.8	17.5
R_{cryst} [a]	16.0	13.6	17.6	16.5	13.5	18.9	14.8	15.5
Average B factor (Å^2)	23.2	16.7	28.0	23.7	15.5	31.5	15.8	12.8
Bond length rmsd (Å)	0.005	0.005	0.005	0.005	0.005	0.006	0.005	0.006
Angle rmsd (˚)	0.889	0.863	0.879	0.917	0.882	0.891	0.894	0.932
Ramachandran plot: (% in each region)[b] Preferred/ Allowed/ Outliers	89.3/8.9/1.8	92.2/6.2/1.6	90.5/7.9/1.6	90.1/9.0/0.9	90.0/8.2/1.8	89.7/9.4/0.9	92.6/6.6/0.8	92.2/6.9/0.8
PDB ID	7L61	7L62	7L63	7L64	7L65	7L66	7L67	7L68

[a]R and Rfree = $100x\sum_h |F_o(h)-F_c(h)| / \sum_h F_o(h)$, where $F_o(h)$ = observed structure factor amplitude and $F_c(h)$ = calculated structure factor amplitude for the working and test sets, respectively.

[b]As defined in Coot.

(A) CRD4 trimmed

```
                                                   ***MetAla
                           5'-gatccgatcttggaggatgattaaatggcc
                               3'-gctagaacctcctactaatttaccgg
                              BamH1

CysProGluAspTrpGlyAlaSerSerArgThrSerLeuCysPheLysLeuTyrAlaLys
                                aagcttgtgtttcaagctgtatgcaaaa
tgtccggaggattggggcgccagcagtagaaca-3'
acaggcctcctaaccccgcggtcgtcatcttgttcga-5'
                                Hind3

GlyLysHisGluLysLysThrTrpPheGluSerArgAspPheCysArgAlaLeuGlyGly
ggaaaacatgagaagaaaacgtggtttgaatctcgagatttttgtcgagctctgggtgga

AspLeuAlaSerIleAsnAsnLysGluGluGlnGlnThrIleTrpArgLeuIleThrAla
gacttagctagcatcaataacaaagaggaacagcaaacaatatggcgattaataacagct

SerGlySerTyrHisLysLeuPheTrpLeuGlyLeuThrTyrGlySerProSerGluGly
agtggaagctaccacaaactgttttggttgggattgacatatggaagcccttcagaaggt

PheThrTrpSerAspGlySerProValSerTyrGluAsnTrpAlaTyrGlyGluProAsn
tttacttggagtgatggttctcctgtttcatatgaaaactgggcttatggagaacctaat

AsnTyrGlnAsnValGluTyrCysGlyGluLeuLysGlyAspProThrMetSerTrpAsn
aattatcaaaatgttgaatactgtggtgagctgaaaggtgacc
                                  5'-gtgaccctactatgtcttggaat
                                       3'-ggatgatacagaacctta
                                    BstE2

AspIleAsnCysGluHisLeuAsnAsnTrpIleCysGlnIle***
gatattaattgtgaacaccttaacaactggatttgccagatatag-3'
ctataattaacacttgtggaattgttgacctaaacggtctatatcttaa-5'
                                            EcoR1
```

(B) CRD4 minimal

```
                           ***MetAlaLeuAlaPheLysLeuTyrAlaLys
   5'-gatccgatcttggaggatgattaaatggccttggctttcaagctgtatgcaaaa
       3'-gctagaacctcctactaatttaccggaaccgaaagttcgacatacgtttt
     BamH1

GlyLysHisGluLysLysThrTrpPheGluSerArgAspPheCysArgAlaLeuGlyGly
                               ctcgagatttttgtcgagctctgggtgga
ggaaaacatgagaagaaaacgtggtttgaatc-3'
ccttttgtactcttcttttgcaccaaacttagagct-5'
                               Xho1

AspLeuAlaSerIleAsnAsnLysGluGluGlnGlnThrIleTrpArgLeuIleThrAla
gacttagctagcatcaataacaaagaggaacagcaaacaatatggcgattaataacagct

SerGlySerTyrHisLysLeuPheTrpLeuGlyLeuThrTyrGlySerProSerGluGly
agtggaagctaccacaaactgttttggttgggattgacatatggaagcccttcagaaggt

PheThrTrpSerAspGlySerProValSerTyrGluAsnTrpAlaTyrGlyGluProAsn
tttacttggagtgatggttctcctgtttcatatgaaaactgggcttatggagaacctaat

AsnTyrGlnAsnValGluTyrCysGlyGluLeuLysGlyAspProThrMetSerTrpAsn
aattatcaaaatgttgaatactgtggtgagctgaaaggtgacc
                                  5'-gtgaccctactatgtcttggaat
                                       3'-ggatgatacagaacctta
                                    BstE2

AspIleAsnCysGluHisLeuAsnAsnTrpIleCysGlnIle***
gatattaattgtgaacaccttaacaactggatttgccagatatag-3'
ctataattaacacttgtggaattgttgacctaaacggtctatatcttaa-5'
                                            EcoR1
```

(C) CRD4 – biotin tag

```
                                                   ***MetAla
                           5'-gatccgatcttggaggatgattaaatggcc
                               3'-gctagaacctcctactaatttaccgg
                              BamH1

CysProGluAspTrpGlyAlaSerSerArgThrSerLeuCysPheLysLeuTyrAlaLys
                                aagcttgtgtttcaagctgtatgcaaaa
tgtccggaggattggggcgccagcagtagaaca-3'
acaggcctcctaaccccgcggtcgtcatcttgttcga-5'
                                Hind3

GlyLysHisGluLysLysThrTrpPheGluSerArgAspPheCysArgAlaLeuGlyGly
ggaaaacatgagaagaaaacgtggtttgaatctcgagattttttgtcgagctctgggtgga

AspLeuAlaSerIleAsnAsnLysGluGluGlnGlnThrIleTrpArgLeuIleThrAla
gacttagctagcatcaataacaaagaggaacagcaaacaatatggcgattaataacagct

SerGlySerTyrHisLysLeuPheTrpLeuGlyLeuThrTyrGlySerProSerGluGly
agtggaagctaccacaaactgttttggttgggattgacatatggaagcccttcagaaggt

PheThrTrpSerAspGlySerProValSerTyrGluAsnTrpAlaTyrGlyGluProAsn
tttacttggagtgatggttctcctgtttcatatgaaaactgggcttatggagaacctaat


AsnTyrGlnAsnValGluTyrCysGlyGluLeuLysGlyAspProThrMetSerTrpAsn
aattatcaaaatgttgaatactgtggtgagctgaaaggtgacc
                                  5'-gtgaccctactatgtcttggaat
                                       3'-ggatgatacagaacctta
                                    BstE2

AspIleAsnCysGluHisLeuAsnAsnTrpIleCysGlnIleLeuAsnAspIlePheGlu
gatattaattgtgaacaccttaacaactggatttgccagatactgaatgacatcttcgaa
ctataattaacacttgtggaattgttgacctaaacggtctatgacttactgtagaagctt

AlaGlnLysIleGluTrpHisGlu***
gcacagaaaatcgagtggcatgagtagctcgagg-3'
cgtgtcttttagctcaccgtactcatcgagctccttaa-5'
                                 EcoR1
```

Figure S1. Modification of the ends of CRD4 expression vector. Green sequence represent synthetic oligonucleotides used to replace restriction fragments at each end of the cDNA sequence for CRD4. Red sequence denotes changes from the sequence of the mannose receptor.

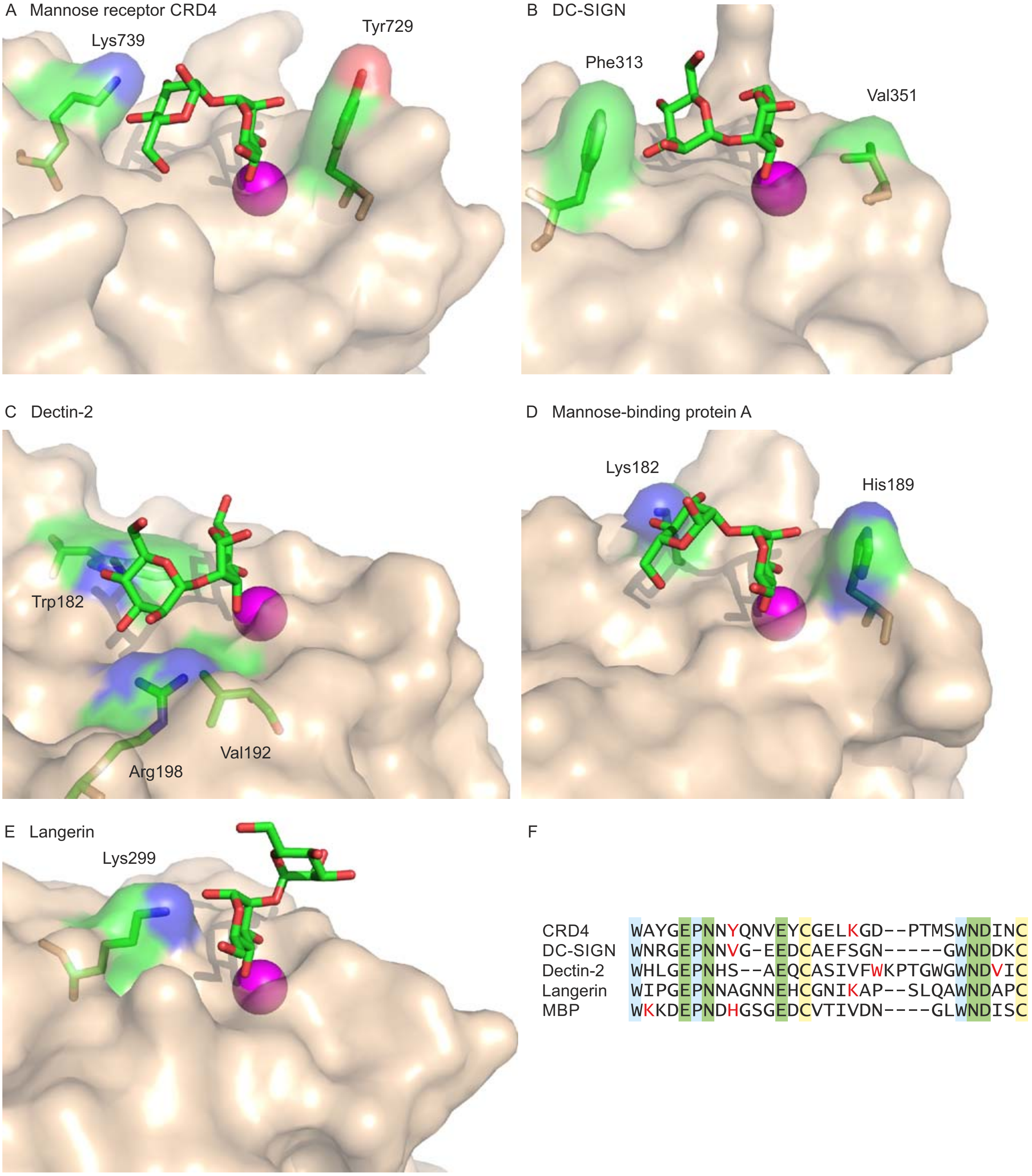

Figure S2. Comparison of mannose-binding sites in C-type CRDs. Structures of Manα1-2Man portions of glycan ligands, with the non-reducing mannose residue in the primary monosaccharide-binding site are shown. *A*, mannose receptor CRD4 from the B protomer of the Manα1-2Man/4 crystal form with bound disaccharide; *B*, DC-SIGN with bound disaccharide, PDB 2it6; *C*, dectin-2 with bound $Man_9GlcNAc_2$ oligosaccharide, PDB 5vyb; *D*, mannose-binding protein A (MBP-A) with bound $Man_6GlcNAc_2Asn$ glycopeptide, PDB 2msb; *E*, langerin with bound disaccharide, PDB 3p5f. All structures are for human proteins except for mannose-binding protein, which is from rat. *F*, comparison of sequences near the principal Ca^{2+}-binding site, with residues that make contact with the disaccharide ligand highlighted in *red*. Shading is as in Figure 2A.

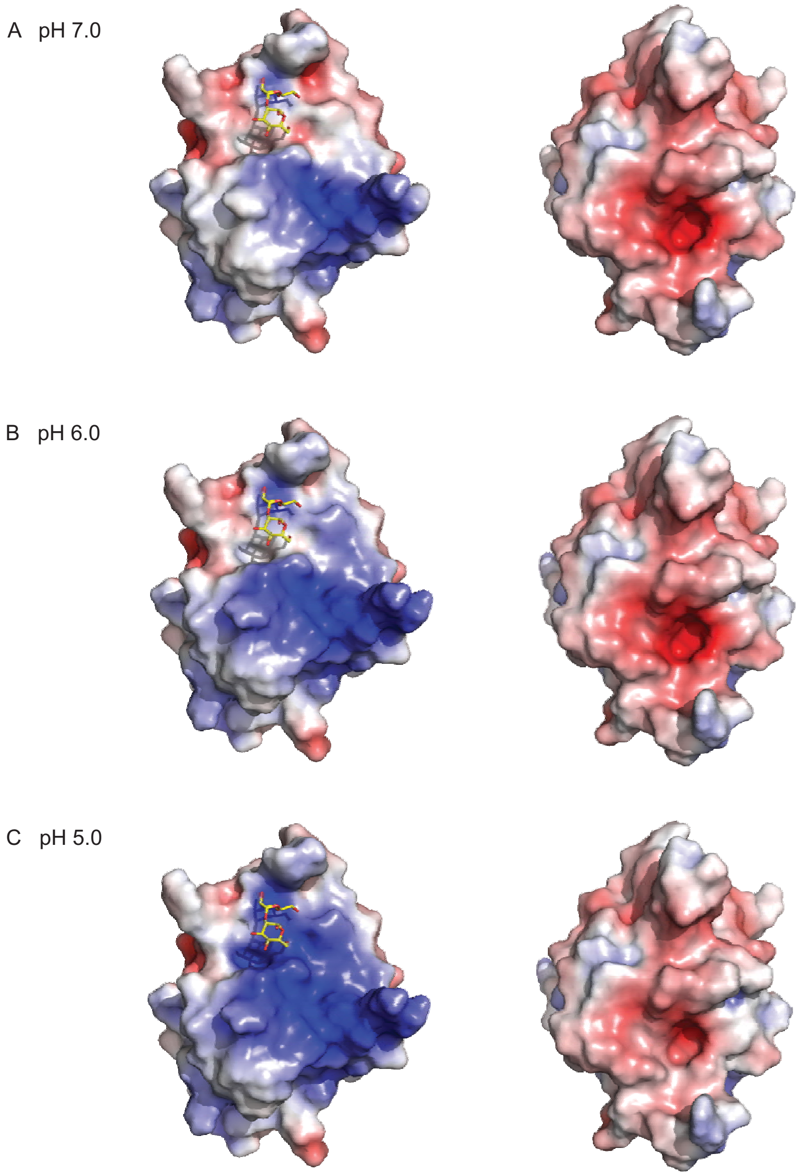

Figure S3. Electrostatic potential surface of CRD4. Partial charges and atom radii for model Manα1-2Man/2 were calculated with the program PDB2PQR at pH 5, 6 and 7. The N- and C-termini were set to be neutral. The calcium ion was added manually. For pH = 5, Glu725, which is a calcium ligand was manually changed to have a charge of -1. The electrostatic map and the molecular surface visualization were calculated using the APBS Electrostatics Plugin in PyMOL. The protein surface is colored according to the electrostatic potential, with negative and positive potentials shown in *red* and *blue* respectively. The scale shown is -5 kT/*e* to +5 kT/*e*.

Supplementary Glycan Microarray Information

based on MIRAGE Guidelines (doi:10.3762/mirage.3)

Array Name: Legacy CFG Microarray
Array Source: DNA Array Core, The Scripps Research Institute, La Jolla, CA

	Description
**1. Sample: Glycan Binding Sample**	
Description of Sample	Human mannose receptor (CD206) carbohydrate-recognition domain 4 (CRD4) expressed in *Escherichia coli* with C-terminal biotin tag and complexed with Alexa647-labelled streptavidin.
Sample modifications	Biotinylated
Assay protocol	CRD4 complexed with Alexa647-strepatividin at 0.4 mg/ml in 150 mM NaCl, 25 mM Tris-Cl, pH 7.8, 2.5 mM EDTA and ~5 mM $CaCl_2$ was diluted 1:2 with 150 mM NaCl, 20 mM Tris-Cl, pH 7.4, 0.05% Tween 20, 1% BSA and incubated with the slides for 1 hour inside a dark humidified chamber. In a second assay, the $CaCl_2$ concentration was increased by 5 mM. After binding, washes were performed with 150 mM NaCl, 20 mM Tris-Cl, pH 7.4, 2 mM $CaCl_2$, 2 mM $MgCl_2$.
**2. Glycan Library**	
Glycan description for defined glycans	Legacy CFG Microarray: synthetic mammalian-type glycans from the Consortium for Functional Glycomics (CFG). Also designated National Center for Functional Glycomics version 1 (NCFGv1). The full list of 601 glycans on version 6.2 of the synthetic mammalian glycan array from the Consortium for Functional Glycomics is provided in Tables S1 and S2. This array contains all glycans from version 5.1 of the array available on the CFG web site except that glycans 230, 252, 473, and 546-550 in the version 5.1 array are not present.
Glycan description for undefined glycans	None.
Glycan modifications	See www.functionalglycomics.org/glycomics/publicdata/primaryscreen.jsp
**3. Printing Surface; e.g., Microarray Slide**	
Description of surface	Scott Nexterion slide H-3D hydrogel coated glass microarray slides (NHS-functionalized hydrogel)
Manufacturer	Applied Microarrays Inc (Tempe, AZ, USA)
Custom preparation of surface	None.
Non-covalent Immobilization	None.

Covalent Immobilization	Hydroxyl or Amine (depending on reducing end) to NHS
**4. Arrayer (Printer)**	
Description of Arrayer	MicroGrid II (Digilab)
Dispensing mechanism	Telechem/ArrayIT SMP 3 contact microarray pins
Glycan deposition	Contact
Printing conditions	Samples of each glycan were diluted to 100 µM in 150 mM sodium phosphate buffer, pH 8.4. Aliquots of 10 µl in 384-well plates were imprinted as 6 replicates of each glycan. Remaining NHS esters were quenched by immersing slides in 50 mM ethanolamine in 50 mM borate buffer, pH 9.2, for 1 h. Blocked slides were washed with water, centrifuged dry, and stored at -20 °C until use.
**5. Glycan Microarray with "Map"**	
Array layout	Each sample was printed in 6 replicates.
Glycan identification and quality control	All glycans have been characterized by mass spectrometry and NMR.<br>Batches of arrays are checked for binding with plant lectins: concanavalin A, *Helix pomatia* agglutinin, *Maackia amurensis* lectin I, *Sambucus nigra* agglutinin, and wheat germ agglutinin.
**6. Detector and Data Processing**	
Scanning hardware	InnoScan 1100AL scanner (Innopsys, Chicago, IL, USA).
Scanner settings	Scanning resolution: 5 µm/pixel;<br>Laser channel: 635 nm<br>PMT gain: 80<br>Power: high
Image analysis software	Mapix 8.2.5 software (Innopsys, Chicago, IL, USA).
Data processing	The gpr files were processed with an in-house workflow using excel macro. No particular normalization method was used. The highest and lowest values were excluded from each set of six replicate spots before the average and standard deviation were calculated.
**7. Glycan Microarray Data Presentation**	
Data presentation	Complete data sets for two screenings of the array are presented as Tables S1 and S2 in Supporting Information. Selected data are presented in graphical format with structures as symbol nomenclature in Figure 3. Binding results are presented as fluorescence intensity of binding in average RFU and standard deviation.
**8. Interpretation and Conclusion from Microarray Data**	
Data interpretation	Data were curated and interpreted manually based on identification of common structural motifs in glycans which gave strong signals on the array.

Conclusions	All but 2 of the 23 glycans that give signals above 10% of the maximum signal contain reducing-end fucose residues or reducing end Manα1-2Man branches. The exceptions are one glycan with multiple reducing end GlcNAc residues and GlcA in α linkage directly to a spacer.
